# Ca^2+^ signals are essential for T-cell proliferation, while Zn^2+^ signals are necessary for T helper cell 1 differentiation

**DOI:** 10.1038/s41420-024-02104-1

**Published:** 2024-07-24

**Authors:** Jana Jakobs, Jens Bertram, Lothar Rink

**Affiliations:** 1 Institute of Immunology, Faculty of Medicine, RWTH Aachen University Hospital, Aachen, Germany; 2 Institute for Occupational, Social and Environmental Medicine, Faculty of Medicine, RWTH Aachen University, Aachen, Germany

**Keywords:** Interferons, Metals, Signal transduction

## Abstract

The regulation of T-cell fate is crucial for the balance between infection control and tolerance. Calcium (Ca^2+^) and zinc (Zn^2+^) signals are both induced after T-cell stimulation, but their specific roles in the fate of activation and differentiation remain to be elucidated. Are Zn^2+^- and Ca^2+^ signals responsible for different aspects in T-cell activation and differentiation and do they act in concert or in opposition? It is crucial to understand the interplay of the intracellular signals to influence the fate of T cells in diseases with undesirable T-cell activities or in Zn^2+^-deficient patients. Human peripheral blood mononuclear cells were stimulated with the Zn^2+^ ionophore pyrithione and thapsigargin, an inhibitor of the sarcoplasmic/endoplasmic reticulum Ca^2+^ ATPase (SERCA). Intracellular Zn^2+^ and Ca^2+^ signals were monitored by flow cytometry and ELISA, quantitative PCR and western blot were used to evaluate T-cell differentiation and the underlying molecular mechanism. We found that Zn^2+^ signals upregulated the early T-cell activation marker CD69, interferon regulatory factor 1 (IRF-1), and Krüppel-like factor 10 (KLF-10) expression, which are important for T helper cell (Th) 1 differentiation. Ca^2+^ signals, on the other hand, increased T-bet and Forkhead box P3 (FoxP3) expression and interleukin (IL)-2 release. Most interestingly, the combination of Zn^2+^ and Ca^2+^ signals was indispensable to induce interferon (IFN)-γ expression and increased the surface expression of CD69 by several-fold. These results highlight the importance of the parallel occurrence of Ca^2+^ and Zn^2+^ signals. Both signals act in concert and are required for the differentiation into Th1 cells, for the stabilization of regulatory T cells, and induces T-cell activation by several-fold. This provides further insight into the impaired immune functions of patients with zinc deficiency.

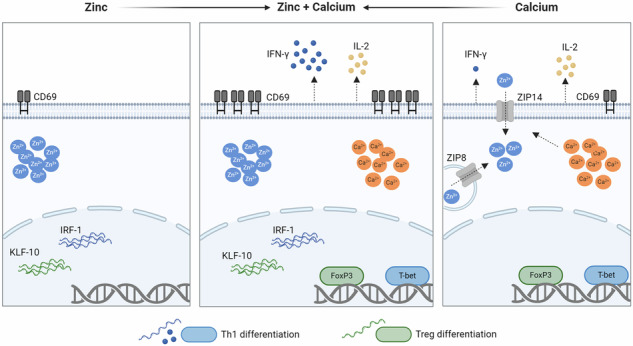

## Introduction

The activation threshold of T lymphocytes and the regulation of T-cell fate are critical for the balance between infection control and tolerance. Once activated, T cells proliferate and differentiate to mediate a highly specific immune response [1]. Stimulation of the T-cell receptor (TCR) causes the distribution of calcium (Ca^2+^) signals throughout the cell, depending on the potency of the antigen [2]. An initial Ca^2+^ signal is induced by inositol-1,4,5-trisphosphate, causing the release of Ca^2+^ from the endoplasmic reticulum (ER) into the cytosol. The depletion of Ca^2+^ from the ER stores is detected by sensors stromal interaction molecule 1 (STIM1) which interacts with ORAI1 in the plasma membrane and thus activating calcium release-activated calcium (CRAC) channels. This induces a store-operated calcium entry (SOCE) from the synaptic cleft. The potentiated Ca^2+^ signal then causes the translocation of the nuclear factor of activated T cells (NFAT) into the nucleus and thereby the expression of important genes for cell proliferation, such as interleukin (IL) 2 [3–5].

In addition to Ca^2+^ signals, zinc (Zn^2+^) signals have also become a focus of interest. Zn^2+^ is an essential trace element which is present in the serum with mean concentrations of 84.9 and 80.6 µg/dL in men and women, respectively [6]. Within cells, Zn^2+^ is mostly bound to proteins such as metallothionein [7], or to Zn^2+^-binding S100 proteins such as calprotectin and calgranulin [8]. Additionally, Zn^2+^ is also present freely or loosely bound in the picomolar to nanomolar range. Intracellular Zn^2+^ signals can occur within seconds after stimulation influencing signaling cascades (zinc flux). Therefore, Zn^2+^ is also regarded as a second messenger [9, 10]. Slower Zn^2+^ signals have also been described in mast cells, occurring a few minutes after receptor stimulation in a Ca^2+^-dependent manner and are referred to as “zinc wave” [11]. Stimulation of cells may additionally cause changes in Zn^2+^ homeostasis after a few days, thereby affecting gene expression [10].

In T cells especially, it has been shown that after stimulation of the TCR via dendritic cells, Zn^2+^ influx from the extracellular space occurs within 1 min, depending on the Zn^2+^ transporter Zip6. This signal occurred mostly in the subsynaptic region of the immunological synapse [9]. This is also seen in CRAC channel-mediated calcium influx where high concentrations of calcium are localized to the subsynaptic region [12, 13]. However, calcium signals were found to rapidly diffuse throughout the cell [14]. When Zn^2+^ influx via Zip6 is inhibited, the activation markers CD25 and CD69 are less expressed, indicating that T-cell activation is impaired [15]. In addition, the Zn^2+^ transporter Zip8 is upregulated after T-cell activation, causing the release of Zn^2+^ from lysosomes into the cytoplasm. However, when Zip8 expression is inhibited, the reduced cytoplasmic Zn^2+^ results in reduced interferon (IFN)-γ expression [16] and thus in T helper cell (Th) 1 differentiation.

Many studies have highlighted the importance of Zn^2+^ in maintaining the balance between Th1/Th2 [17, 18] in inducing regulatory T cells [19, 20]. The interplay between Zn^2+^ and Ca^2+^ signaling has also been partly investigated. Extracellular Zn^2+^ can sustain Ca^2+^ signaling after T-cell receptor stimulation [9], and in the human T-cell line Jurkat, it was seen that different stimulants used for T-cell activation induce either a Ca^2+^ or a Zn^2+^ signal [21]. However, the mechanism by which Zn^2+^ and Ca^2+^ act in parallel has yet to be examined. Therefore, we investigated whether Zn^2+^ and Ca^2+^ signals are responsible for different aspects in T-cell activation and differentiation. We focused on the single and combined effects of Zn^2+^ and Ca^2+^ signals independent of other activating stimulants.

## Results

### Induction of Zn^2+^ and Ca^2+^ signals

**Fig. 1:**
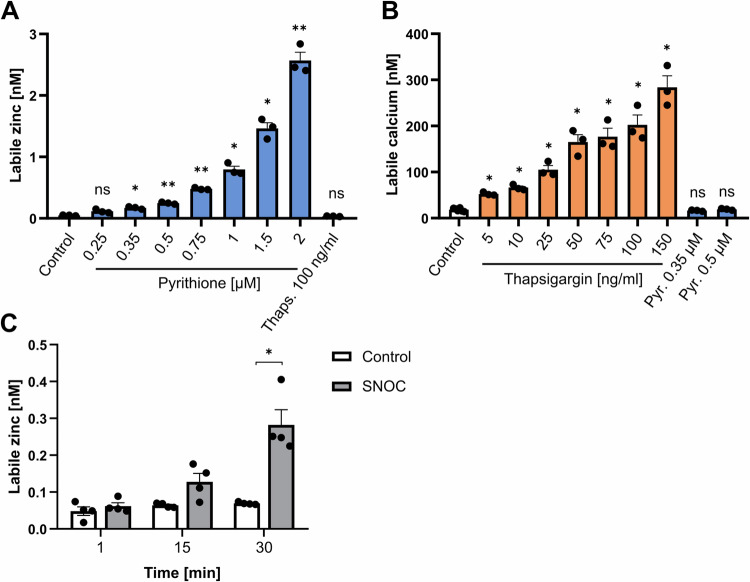
Induction of Zn^2+^ and Ca^2+^ signals in PBMC. PBMC were stained with FluoZin-3 AM for Zn^2+^ measurements or with Fluo-4 for Ca^2+^ measurements and then stimulated with pyrithione (Pyr.), thapsigargin (Thaps.) or *S*-nitrosocysteine (SNOC). **A** 10 min after stimulation the intracellular Zn^2+^ or **B** Ca^2+^ concentration was measured using flow cytometry. **C** In addition, cells were stimulated with 2 mM SNOC and Zn^2+^ concentration was measured at 15 min time intervals after stimulation. Data are presented as mean + SEM with *n* = 3 (**A**), *n* = 3–6 (**B**) and *n* = 4 (**C**) experiments. Statistical significance was determined by one-way ANOVA with Dunnett’s multiple comparisons test (**A**, **B**) or Friedman test with Dunn’s multiple comparisons test (**C**) (*p < 0.05; **p < 0.01).

To investigate the individual and the combined effects of Zn^2+^ and Ca^2+^ signals, we separately induced intracellular Zn^2+^ signals using the Zn^2+^ ionophore pyrithione (Fig. 1A) and Ca^2+^ signals using thapsigargin in peripheral blood mononuclear cells (PBMC) (Fig. 1B). Thapsigargin inhibits the sarcoplasmic/endoplasmic reticulum Ca^2+^ ATPase (SERCA) and thereby reduces the transport of Ca^2+^ into the endoplasmic reticulum, which results in an accumulation of Ca^2+^ in the cytosol [22]. PBMC were stimulated with increasing concentrations of pyrithione (0.25–2 µM) or thapsigargin (5–150 µM). First, the cell viability was investigated by propidium iodide staining and no significant increase in dead PI^+^ lymphocytes was observed (Fig. S1A, B). Even with the combined stimulation with pyrithione and thapsigargin, no significant increase in dead PI^+^ lymphocytes was found (Fig. S1C). Then, the induction of intracellular Zn^2+^ and Ca^2+^ signals was measured by flow cytometry 10 min after stimulation. We confirmed that pyrithione and thapsigargin increased the intracellular Zn^2+^ and Ca^2+^ concentrations, respectively, in a dose-dependent manner (Fig. 1A, B). 0.35 and 0.5 µM pyrithione increased the mean intracellular Zn^2+^ concentration from 0.048 to 0.171 and 0.248 nM, respectively. For the following experiments, 0.35 and 0.5 µM pyrithione were used, since Zn^2+^ acts in the nanomolar concentration range [23].

For Ca^2+^ signals, 50 ng/ml thapsigargin was used because it significantly increased mean intracellular Ca^2+^ levels from 18.4 to 164.5 nM. At 220 nM intracellular Ca^2+^ and above, maximal proliferation is induced in T cells [24]. However, to be able to study the effect of Zn^2+^ in parallel, we decided to use a lower Ca^2+^ signal. Importantly, thapsigargin did not cause an increase in intracellular Zn^2+^ and pyrithione did not induce a Ca^2+^ signal (Fig. 1A, B).

In addition to pyrithione, which imports extracellular Zn^2+^ into the cell, the NO donor *S*-nitrosocysteine (SNOC) has been used to release Zn^2+^ from intracellular sources as shown in some signaling pathways [25, 26]. SNOC releases Zn^2+^ bound to metallothionein by S-nitrosylation [27, 28]. In initial experiments, SNOC elicited only a very small Zn^2+^ signal 10 min after stimulation, so we examined intracellular Zn^2+^ concentration in a 15-min interval. SNOC significantly increased the intracellular free Zn^2+^ concentration after 30 min which is slower than pyrithione (Fig. 1C). Therefore, we decided to preincubate with SNOC for 30 min in the following experiments and then stimulate additionally with thapsigargin.

### Zn^2+^ and Ca^2+^ signals activate T cells in a p38 MAPK-dependent manner

**Fig. 2:**
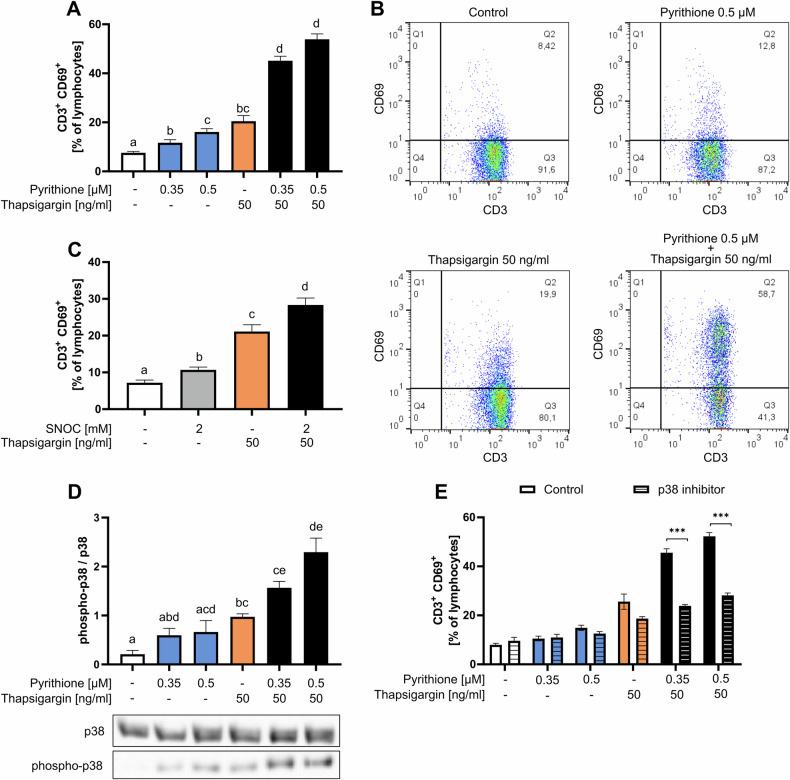
Zn^2+^ and Ca^2+^ signals activate T cells synergistically. **A** PBMC were stimulated with pyrithione and thapsigargin at the indicated concentrations and incubated for 48 h at 37 °C. Activated T cells were then identified by CD3 and CD69 antibody staining. **B** Exemplary analysis shows the population of CD3^+^ T cells after pyrithione + thapsigargin stimulation. **C** PBMC were stimulated with *S*-nitrosocysteine (SNOC) and thapsigargin and incubated for 48 h at 37 °C. Activated T cells were then identified by CD3 and CD69 antibody staining. **D** PBMC were stimulated with pyrithione and thapsigargin at the indicated concentrations and incubated for 48 h. Protein expression of phospho-p38 was examined by western blot and normalized to p38 expression. A representative western blot is shown. **E** PBMC were preincubated for 30 min with the p38 MAPK inhibitor SB202190 (10 µM) and then stimulated with pyrithione and thapsigargin at the indicated concentrations. After 48 h, activated T cells (CD3^+^CD69^+^) were determined by flow cytometry. The control data shown in (**E**) are also included in the data shown in (**A**). Data are presented as mean + SEM with *n* = 13 (**A**), *n* = 6 (**C**) and *n* = 5 (**D**, **E**) experiments. Statistical significance was determined by one-way ANOVA with Tukey’s multiple comparisons test and significantly different results (*p* < 0.05) have no common identification letter (**A**, **C**). Statistical significance was determined by two-way ANOVA with Sidak’s multiple comparisons test (***p < 0.001).

First, we investigated the activation of T cells after stimulation with pyrithione, thapsigargin, and a combination of both. Therefore, the surface expression of the early activation marker CD69 in CD3^+^ T cells was measured 48 h after stimulation with flow cytometry. We found that pyrithione and thapsigargin individually upregulate CD69 expression in T cells in 12–20% of lymphocytes (Fig. 2A). However, when PBMC were stimulated simultaneously with different concentrations of pyrithione and thapsigargin, 45.1 and 53.9% of lymphocytes were activated, respectively, and a distinct CD3^+^CD69^+^ population emerged (Fig. 2B). Therefore, Zn^2+^ and Ca^2+^ signals synergistically induce CD69 expression, which is important for the retention of activated T cells in the lymph node [29]. In comparison, SNOC increased CD69 expression, and when combined with thapsigargin, CD69 expression was also further increased (Fig. 2C). However, on average, SNOC + thapsigargin activated fewer T cells (28.3%) than pyrithione + thapsigargin (0.5 µM pyrithione +thapsigargin: 53.9%). Therefore, in the following study, we focused on Zn^2+^ signals induced by pyrithione and not by SNOC. However, this experiment shows that also intracellular Zn^2+^ release is able to synergize with Ca^2+^ signals.

We next investigated proteins involved in the upregulation of CD69 by Ca^2+^ and Zn^2+^ signals. We focused on p38 mitogen-activated protein kinase (p38 MAPK), which is activated upon T-cell receptor stimulation, by CD28 and IL-12/IL-18 receptor signaling. Further, p38 MAPK is important for CD69 expression in cytokine-stimulated T cells [30], and is critical for differentiation into Th1 or Th2 cells [31] and has been shown to be activated by Zn^2+^ [32]. Therefore, we investigated whether T-cell activation by pyrithione and thapsigargin is also mediated via p38 MAPK. First, we found that phosphorylation of p38 was increased in PBMC after thapsigargin stimulation and was further enhanced by additional stimulation with pyrithione (Fig. 2D). Therefore, we preincubated PBMC for 30 min with the specific p38 MAPK inhibitor SB202190 and subsequently stimulated with pyrithione and thapsigargin. It was found that the strong expression of CD69 induced by pyrithione and thapsigargin was significantly reduced when p38 MAPK was inhibited (Fig. 2E).

### Only Zn^2+^ plus Ca^2+^ induces differentiation into Th1 cells

**Fig. 3:**
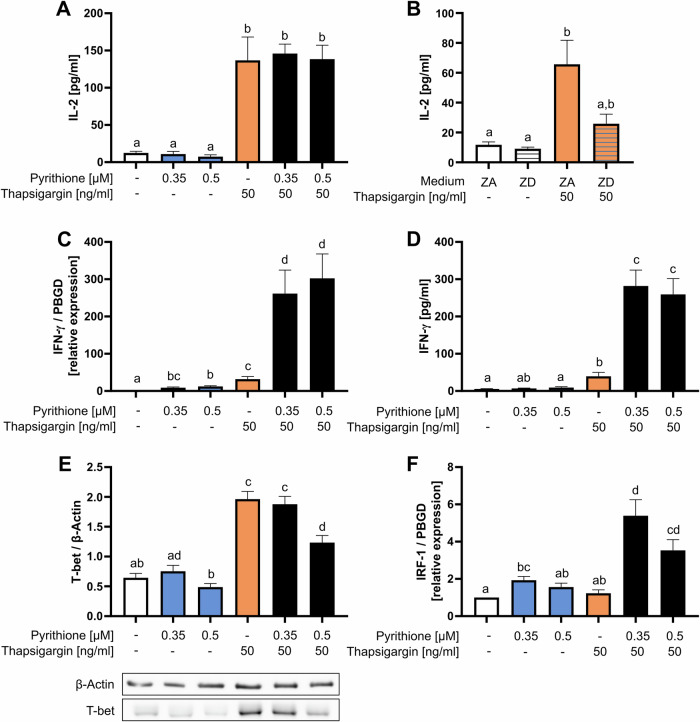
Zn^2+^ and Ca^2+^ induce differentiation into Th1 cells. **A** PBMC were stimulated at the same time with pyrithione and thapsigargin at the indicated concentrations. After 48 h, released IL-2 was measured by ELISA. **B** PBMC were stimulated with thapsigargin in Zn^2+^-adequate (ZA) or Zn^2+^-deficient (ZD) medium and the IL-2 production was measured after 48 h by ELISA. **C** PBMC were stimulated as described in (**A**) and the IFN-γ mRNA expression was examined by qPCR after 3 h and normalized to PBGD expression. **D** PBMC were stimulated as described in (**A**) and after 48 h, IFN-γ in the supernatant was measured by ELISA. **E** PBMC were stimulated as described in (**A**) and the protein expression of transcription factor T-bet was examined by western blot 48 h after stimulation and normalized to β-actin. A representative western blot is shown. **F** PBMC were stimulated as described in (**A**) and the IRF-1 expression was examined by qPCR after 3 h and relative mRNA expression was determined by 2^−ΔΔCt^ method. Data are presented as mean + SEM with *n* = 8 (**A**), *n* = 12 (**B**), *n* = 10 (**C**), *n* = 9 (**D**), *n* = 14 (**E**) and *n* = 12 (**F**) experiments. Statistical significance was determined by one-way ANOVA with Tukey’s multiple comparisons test (**A**, **C**–**F**) or with Friedman test with Dunn’s multiple comparison test (**B**). Significantly different results (*p* < 0.05) have no common identification letter.

After T-cell activation, the release of growth factor interleukin (IL)-2 is important for T-cell proliferation and the development of regulatory T cells (Treg) [33]. In Zn^2+^ deficiency, IL-2 expression is reduced [34, 35] whereas Zn^2+^ had no impact on IL-2 expression in mixed lymphocyte cultures [19]. Therefore, we were interested in the individual functions of Zn^2+^ and Ca^2+^ signals in IL-2 regulation. IL-2 secretion by PBMC was measured 48 h after stimulation by ELISA. The results showed that stimulation with pyrithione had no effect on IL-2 expression, whereas thapsigargin increased IL-2 secretion (Fig. 3A). Pyrithione did not enhance IL-2 production by thapsigargin. This was also not seen in experiments with lower thapsigargin concentrations. 10 ng/ml thapsigargin did not significantly induce IL-2 production with or without pyrithione costimulation (Fig. S2). These results suggest that only Ca^2+^, but not Zn^2+^ signaling is critical for IL-2 release. However, we found that the induction of IL-2 by thapsigargin depends on extracellular Zn^2+^, as the thapsigargin stimulation in Zn^2+^-deficient (ZD) medium did not induce a significant release of IL-2 (Fig. 3B).

**Fig. 4:**
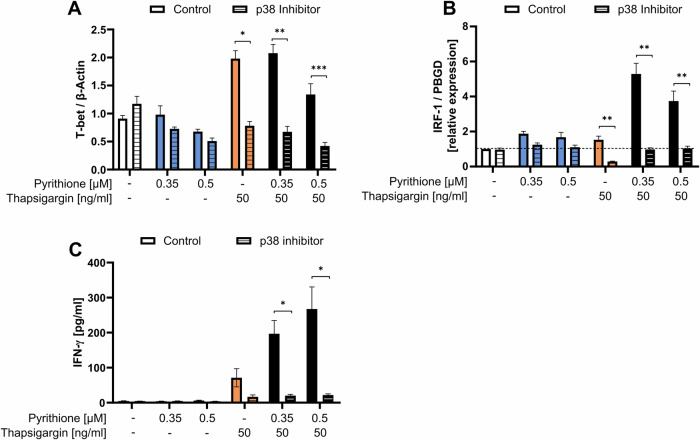
Th1 differentiation after p38 MAPK inhibition. PBMC were preincubated for 30 min with the p38 MAPK inhibitor SB202190 (10 µM) and then stimulated with pyrithione and thapsigargin at the indicated concentrations. **A** Protein expression of the transcription factor T-bet was examined by western blot 48 h after stimulation and normalized to β-actin. The control data shown in (**A**) are also included in the data shown in Fig. 3E. **B** IRF-1 expression was also analyzed by qPCR after 3 h and relative mRNA expression was determined by 2^−ΔΔCt^ method. The control data shown in (**B**) are also included in the data shown in Fig. 3F. **C** After 48 h, IFN-γ was measured by ELISA. The control data shown in (**C**) are partially included in the data shown in Fig. 3D. Data are presented as mean + SEM with *n* = 6 (**A**), *n* = 7 (**B**) and *n* = 7 (**C**) experiments. Statistical significance was determined by Friedman test with Dunn’s multiple comparisons test (**A**, **B**) or by one-way ANOVA with Sidak’s multiple comparisons test (**C**) (*p < 0.05; **p < 0.01; ***p < 0.001).

Further, it has already been shown that Zn^2+^ affects the balance of Th1 and Th2 cells and induces Treg cells [36, 37]. Therefore, we investigated how pyrithione and thapsigargin affect T-cell differentiation. To investigate the differentiation into Th1 cells, the characteristic cytokine IFN-γ was determined. By qPCR and ELISA, IFN-γ was shown to be partially upregulated by pyrithione and thapsigargin, but the combined stimulation greatly increased its expression (Fig. 3C, D). This was not only seen in PBMC but also in isolated naïve CD4^+^ T cells (Fig. S3). Therefore, we examined T-bet, which is critical for Th1 differentiation and a key transcription factor of IFN-γ expression [38]. Interestingly, T-bet was regulated differently from IFN-γ. T-bet was induced by thapsigargin alone and was not further increased by pyrithione but rather decreased with high pyrithione concentrations (Fig. 3E). Thus, T-bet cannot explain the increased expression of IFN-γ by thapsigargin and pyrithione. Therefore, we further examined the transcription factor IRF-1 (interferon regulatory factor 1), which is also involved in Th1 differentiation. IRF-1 increases the responsiveness to IL-12, and Irf1^−/−^ CD4^+^ T cells showed reduced IFN-γ production [39, 40]. In our experiments, IRF-1 expression was examined at the mRNA level 3 h after stimulation and was significantly increased by 0.35 µM pyrithione and by the combination of pyrithione and thapsigargin (Fig. 3F). In contrast, thapsigargin did not affect IRF-1 expression. The Ca^2+^ dependence of T-bet and the Zn^2+^ dependence of IRF-1 could jointly explain the increase in IFN-y in the presence of simultaneous Zn^2+^ and Ca^2+^ signals. Moreover, the induction of T-bet and IRF-1 (Fig. 4A, B) and the release of IFN-γ (Fig. 4C) by thapsigargin and pyrithione are dependent on p38 MAPK activity.

Additionally, we investigated if Zn^2+^ induces T-cell activation and IFN-γ expression in dependence of the protein kinase C (PKC). Phorbol 12-myristate 13-acetate (PMA) and calcium ionophores are commonly used for T-cell activation [41] and PMA stimulation was already shown to increase intracellular Zn^2+^ levels in Jurkat T cells [21]. Thus, the question arose if Zn^2+^ mimics the effect of PMA costimulation. Thus, experiments were performed in the presence of the PKC inhibitor bisindolylmaleimide (BIS) II (Fig. S4) [42]. We found that thapsigargin and pyrithione still synergistically increased the CD69 expression (Fig. S4A) and that also the IFN-γ expression by thapsigargin and pyrithione was not reduced in the presence of the PKC inhibitor (Fig. S4B). Thus, the effects of Zn^2+^ are not dependent on PKC and Zn^2+^ does not mimic the costimulatory effect of PMA.

**Fig. 5:**
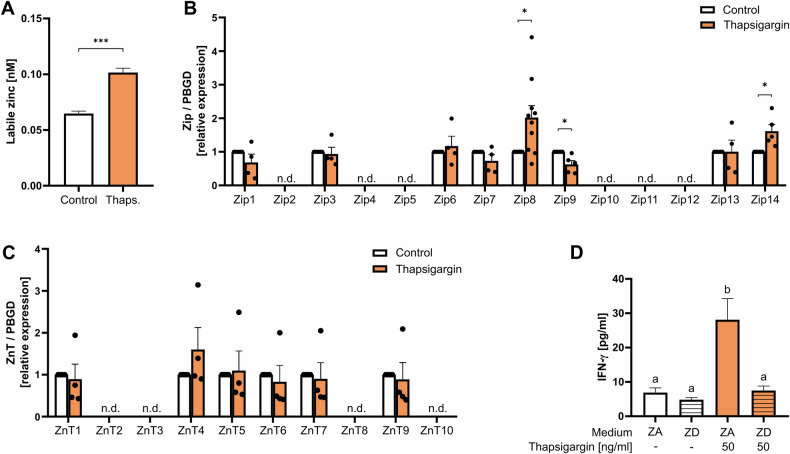
Thapsigargin increases IFN-γ by altering Zn^2+^ homeostasis. PBMC were left untreated, stimulated with 50 ng/ml thapsigargin. **A** 48 h after thapsigargin stimulation the intracellular Zn^2+^ concentration was determined by FluoZin-3. **B** 3 h after stimulation the mRNA expression of the Zn^2+^ importer and **C** the Zn^2+^ exporter were analyzed by qPCR and relative expression was determined by ${{\bf{2}}}^{{\boldsymbol{-}}{\boldsymbol{\Delta }}{\boldsymbol{\Delta }}{{\boldsymbol{C}}}_{{\boldsymbol{T}}}}$ method. The expression of some transporter was not detectable (n.d.). **D** PBMC were preincubated for 15 min in Zn^2+^-adequate (ZA) or Zn^2+^-deficient (ZD) medium and were subsequently stimulated with thapsigargin. After 48 h, IFN-γ was measured in the supernatant by ELISA. Data are presented as mean + SEM with *n* = 5 (**A**), *n* = 4–10 (**B**), *n* = 4 (**C**) and *n* = 9 (**D**) experiments. Statistical significance was determined by paired *t*-test (*p < 0.05; ***p < 0.001) (**A**–**C**) or by Friedman test with Dunn’s multiple comparisons test (**D**). Significantly different results (*p* < 0.05) have no common identification letter (**D**).

We next investigated how thapsigargin causes a small IFN-γ release even in the absence of a Zn^2+^ signal via pyrithione. As shown above in Fig. 1A, thapsigargin does not induce a rapid Zn^2+^ flux. Therefore, we examined the effect of thapsigargin on the intracellular Zn^2+^ homeostasis and found that intracellular Zn^2+^ was significantly increased 48 h after stimulation (Fig. 5A). This can be explained by the altered Zn^2+^ transporter expression 3 h after stimulation with thapsigargin (Fig. 5B, C). The mRNA expression of the Zn^2+^ importer Zip8 and Zip14 were significantly upregulated whereas Zip9 was downregulated by thapsigargin (Fig. 5B). The Zn^2+^ exporter ZnT1, ZnT4-7 and ZnT9 were not affected (Fig. 5C) whereas Zip2, Zip4, Zip5, Zip10-12, ZnT2, ZnT3, ZnT8 and ZnT10 were not detectable in PBMC.

In comparison we found that the stimulation with pyrithione and thapsigargin also significantly increased Zip14 expression (Fig. S5A). Zip6 was significantly upregulated which shows the same trend as with thapsigargin stimulation alone. In contrast, Zip8 was downregulated. Furthermore, the zinc exporter ZnT1 was upregulated and ZnT5 and ZnT6 were downregulated with combined thapsigargin and pyrithione stimulation (Fig. S5B).

Given this impact of thapsigargin on Zn^2+^ homeostasis, we wondered whether thapsigargin still induces the release of IFN-γ even in the absence of extracellular Zn^2+^. For this purpose, PBMC were stimulated with thapsigargin in normal cell culture medium or Chelex-treated Zn^2+^-deficient (ZD) medium. Interestingly, without extracellular Zn^2+^, thapsigargin did not induce the release of IFN-γ (Fig. 5D). Since the Chelex^®^ resin used to produce the ZD medium removes divalent cations, we measured the depletion of Zn, Ca, Mg, Cu, Fe, and Mn (Table S1). Subsequently, the removed metal ions including Zn^2+^ were reconstituted accordingly (3.59 µM ZnSO_4_/234.6 µg/L Zn) and the Zn^2+^-reconstituted (ZR) medium was measured again. A Zn^2+^-deficient (ZD) medium in these experiments was obtained by reconstituting all metal ions except Zn^2+^ (Table S1). We found that in ZR culture medium, thapsigargin-induced IFN-γ expression was significantly increased in comparison to the stimulation in ZD medium (Fig. S6). Therefore, these results conclude that thapsigargin stimulation increases the IFN-γ expression in a Zn^2+^-depend way by altering the Zn^2+^ transporter expression and thus increasing intracellular Zn^2+^.

### Th2, Th17 and Treg differentiation after Zn^2+^ and Ca^2+^ signals

**Fig. 6:**
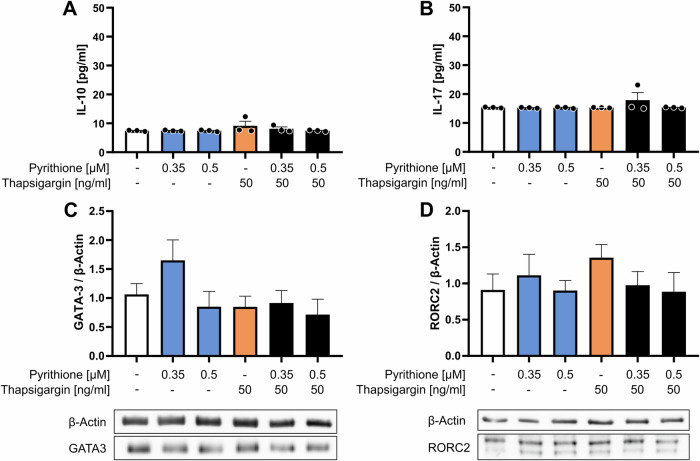
Zinc and calcium signals do not induce Th2 and Th17 differentiation. PBMC were stimulated at the same time with pyrithione and thapsigargin at the indicated concentrations and differentiation into Th2 and Th17 was determined. **A** 48 h after stimulation, IL-10 (Th2) and **B** IL-17 (Th17) were measured by ELISA. **C** Protein expression of the transcription factor GATA-3 (Th2) and **D** RORC2 (Th17) were examined by western blot 48 h after stimulation and normalized to β-actin. For each a representative western blot is shown. Data are presented as mean + SEM with *n* = 3 (**A**, **B**), *n* = 5 (**C**, **D**) experiments. Statistical significance was tested by one-way ANOVA with Tukey’s multiple comparisons test (**A**, **B**) or Friedman test with Dunn’s multiple comparisons test (**D**), but no significances were found.

In addition to the markers for Th1 differentiation, we also examined characteristic cytokines and transcription factors of Th2, Th17 and Treg cells by ELISA and western blot. The cytokines IL-10 (Th2) and IL-17 (Th17) were not induced either by Zn^2+^ or Ca^2+^ signals (Fig. 6A, B). The concentrations were below the detection limits of the respective ELISA. The transcription factors GATA-3 (Th2) and RORC2 (Th17) were also not altered by thapsigargin and pyrithione (Fig. 6C, D). Zn^2+^ and Ca^2+^ signals alone accordingly did not induce differentiation into Th2 and Th17 cells.

**Fig. 7:**
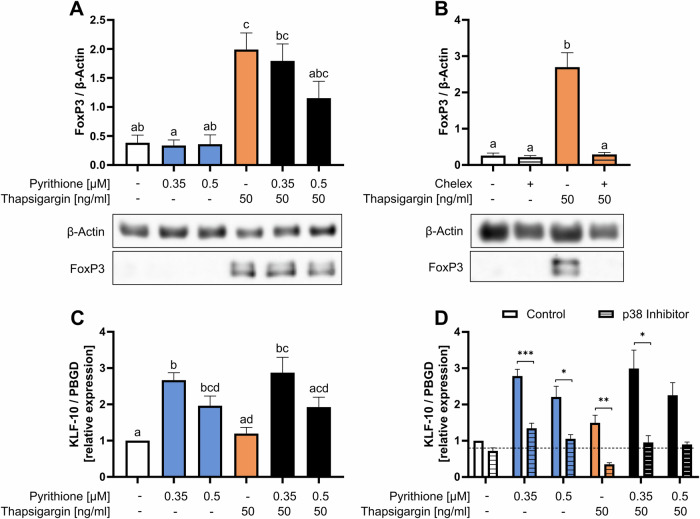
Zn^2+^ and Ca^2+^ are necessary for Treg differentiation. **A** PBMC were stimulated at the same time with pyrithione and thapsigargin. Protein expression of the transcription factor FoxP3 was examined by western blot 48 h after stimulation and normalized to β-actin. A representative western blot is shown. **B** In addition, PBMC were preincubated for 15 min in normal or Chelex-treated Zn^2+^-deficient medium and were subsequently stimulated with thapsigargin. FoxP3 was also examined by western blot after 48 h and normalized to β-actin. A representative western blot is shown. **C** PBMC were stimulated at the same time with pyrithione and thapsigargin. 3 h after stimulation KLF-10 expression was examined by qPCR and normalized to PBDG. **D** In addition, PBMC were preincubated with the p38 MAPK inhibitor SB202190 (10 µM) for 30 min and then stimulated with pyrithione and thapsigargin. KLF-10 expression was also examined after 3 h by qPCR. The control data shown in (**D**) are also included in the data shown in Fig. 7C. Data are presented as mean + SEM with *n* = 9 (**A**), *n* = 6 (**B**), *n* = 12 (**C**) and *n* = 7 (**D**) experiments. Statistical significance was determined by one-way ANOVA with Tukey’s multiple comparisons test (**A**–**C**) or with Sidak’s multiple comparisons test (**D**). Significantly different results (*p* < 0.05) have no common identification letter (**A**, **C**) and *p < 0.05; **p < 0.01; ***p < 0.001.

Forkhead box P3 (FoxP3) is a characteristic transcription factor of Treg cells [43]. FoxP3 was induced by thapsigargin but was not affected by pyrithione (Fig. 7A). This result was unexpected, as Zn^2+^ supplementation has already been shown to stabilize FoxP3 expression and to induce Treg [19, 20]. Therefore, we additionally investigated whether thapsigargin can also induce FoxP3 in Zn^2+^ depletion. It was found that in Chelex-treated Zn^2+^-deficient medium thapsigargin can no longer induce FoxP3 (Fig. 7B). Like pyrithione, ZnSO_4_ had no additional effect on FoxP3 expression. We further examined the transcription factor KLF-10 (Krüppel-like factor 10), which suppresses differentiation into Th1 and instead promotes differentiation into Treg [44]. KLF-10 was significantly induced by pyrithione but was not affected by thapsigargin, nor further induced by the combination with pyrithione and thapsigargin (Fig. 7C). The induction of KLF-10 by pyrithione was also dependent on p38 MAPK (Fig. 7D).

## Discussion

In this study we focused on the mechanism of how Zn^2+^ and Ca^2+^ signals act in parallel in the induction of T-cell activation and differentiation. We found that Zn^2+^ and Ca^2+^ regulate different aspects but that the presence of both signals is critical for T-cell activation, Th1 differentiation and Treg function. As a marker of early T-cell activation, we investigated CD69 expression, which is important for the retention of activated T cells in lymph nodes but is also involved in T-cell differentiation [29, 45]. We have shown that Ca^2+^ and Zn^2+^ signals individually cause a low level of T-cell activation (CD69), but that both signals in combination amplify the CD69 expression several-fold. Zn^2+^ and Ca^2+^ signals upregulate the CD69 expression in CD3^+^ T cells in a p38 MAPK-dependent manner. However, when p38 MAPK was inhibited, the CD69 expression was not fully reduced to the control level, suggesting that on the one hand p38 MAPK, but also other signaling molecules are important for the effects of Zn^2+^ and Ca^2+^ on CD69 expression. Furthermore, we have investigated if the Zn^2+^ signal mimics the costimulatory effect seen by PMA [41] since it was shown that PMA stimulation increases intracellular Zn^2+^ levels [21]. Thus, we have examined the role of PKC by using the inhibitor BIS II. Our results demonstrate that the synergistic effects of thapsigargin and pyrithione do not depend on the PKC.

Further, the release of IL-2, which is important for cell proliferation, was only induced by Ca^2+^ signals. Ca^2+^ activates calcineurin, which in turn dephosphorylates NFAT to induce IL-2 transcription [5]. Zn^2+^ signals had no effect on the IL-2 expression. However, in alternative T-cell stimulation models, the importance of Zn^2+^ in regulating IL-2 has been previously demonstrated. After stimulation with concanavalin A or PMA and ionomycin high extracellular Zn^2+^ has been shown to inhibit calcineurin and IL-2 expression [46]. Conversely, in Zn^2+^ deficiency, IL-2 expression has been shown to be downregulated in a CREMα-dependent manner [34, 35]. This is consistent with our results showing that thapsigargin-induced IL-2 release is not induced in Zn^2+^-deficient medium. Additionally, Zn^2+^ did not affect the release of IL-2 in mixed lymphocyte cultures [19].

Moreover, we focused on the role of Zn^2+^ and Ca^2+^ in the differentiation into Th1 cells. The individual signals barely triggered the release of IFN-γ, but in combination the IFN-γ expression was highly increased. Unexpectedly, the major transcription factor T-bet was differentially regulated than IFN-γ and was only induced by Ca^2+^ but not Zn^2+^. Thus, Zn^2+^ seems to induce IFN-γ independently from T-bet expression, so accordingly we investigated IRF-1 regulation. IRF-1 is an important regulator of Th1 differentiation which enhances IL-12 signaling and thus increased IFN-γ expression. In previous studies Irf1^−/−^ CD4^+^ T cells showed reduced IFN-γ production [39, 40] and IRF-1 is also upregulated by Zn^2+^ in unstimulated PBMC [47]. In our experiments we show that Zn^2+^ but not Ca^2+^ upregulated IRF-1. Thus, only when Zn^2+^ and Ca^2+^ signals were combined, resulting in IRF-1 and T-bet upregulation respectively, the IFN-γ release increased several-fold. This agrees well with the observation that people in Zn^2+^ deficiency have an impaired IFN-γ release [48].

However, Zn^2+^ might affect IFN-γ release by other factors as well and future studies are necessary to clarify the role of IRF-1. For example, it was previously published that Zn^2+^ deficiency affects IFN-γ on the post-transcriptional level [49]. Additionally, experiments were performed in PBMC which have mixed populations of T cells, B cells, natural killer (NK) cells, monocytes and dendritic cells. Thus, the regulation of IFN-γ by T-bet and IRF-1 might not only be specific for T cells and other cell types, particularly NK cells [50], could be masking the T-cell response. Nevertheless, we confirmed the synergistic effect of thapsigargin and pyrithione in IFN-γ production in isolated CD4^+^ T cells.

Further, we focused on the low IFN-γ release by single Ca^2+^ signals. We found that Ca^2+^ upregulates specific Zn^2+^ transporters, leading to an increase in intracellular Zn^2+^ and thus upregulating IFN-γ to a small extent. The Zn^2+^ transporter Zip4 and Zip14 are located in the plasma membrane [51] and their upregulation results in increased import of Zn^2+^ from the extracellular space into the cell. Zip8 is located in the lysosomal membrane [51] and its upregulation results in increased transport of Zn^2+^ from the lysosomes into the cytoplasm.

Zip8 is already known to be strongly upregulated after T-cell activation [16]. Aydemir et al. have shown that Zip8 is important for IFN-γ expression because the released Zn^2+^ via Zip8 inhibits calcineurin and thus sustains CREB phosphorylation which results in increased IFN-γ [16]. The importance of Zip8 and Zn^2+^ in IFN-γ expression was further highlighted in studies with proton-pump inhibitors [52] and is currently further investigated in our group in the elderly population. Complementary to Aydemir et al. [16] our results suggest that the upregulation of Zip8 is induced by the Ca^2+^ signal which occurs after T-cell receptor stimulation. However, the stimulation with pyrithione and thapsigargin downregulated Zip8 in our experiments, demonstrating a different zinc transporter pattern 3 h after stimulation than in T-cell stimulation via the TCR 48 h after stimulation [16].

Zip14 is evolutionary closely related to Zip8 [51] and its upregulation was shown before by phytohaemagglutinin and T-cell receptor stimulation in PBMC, respectively. In addition, Zip14 was also upregulated by the combined stimulation with pyrithione and thapsigargin. Zip14 is located in the plasma membrane and emphasizes the importance of extracellular Zn^2+^ [51]. However, the function of Zip14 in lymphocytes remains to be elucidated [16, 53].

The mRNA of the transporter Zip9-11 was downregulated, but as the overall Zn^2+^ increased, this seemed not to have a great impact. As a result of the altered Zn^2+^ transporter expression we measured an increase in intracellular Zn^2+^. Zn^2+^ was essential for the Ca^2+^-induced IFN-γ release, since under extracellular Zn^2+^ deficiency thapsigargin did not induce IFN-γ release. This is comparable to the described “homeostatic Zn^2+^ signal”, because the Zn^2+^ concentration was increased 48 h after stimulation due to altered transporter expression [23].

In addition to Th1 differentiation, we showed the importance of Zn^2+^ and Ca^2+^ in Treg differentiation. Ca^2+^ signals induced the expression of FoxP3, but Zn^2+^ had no effect on FoxP3. This result was unexpected, as Zn^2+^ supplementation has already been shown to be important for FoxP3 expression and to induce Treg in other T-cell activation models [19, 20]. Therefore, we examined the expression of FoxP3 after stimulation with thapsigargin under extracellular Zn^2+^ deficiency and found that FoxP3 was no longer upregulated. This confirms previous observations that Zn^2+^ is necessary for the stabilization of FoxP3 [19]. In contrast, KLF-10 was only upregulated by Zn^2+^ but not by Ca^2+^ signaling. KLF-10 suppresses differentiation into Th1 and instead promotes differentiation into Treg [44].

While Th1 and Treg cell differentiation was induced with pyrithione and thapsigargin stimulation, a differentiation into Th2 and Th17 cells was not observed, as no IL-10 and IL-17 was produced. However, the importance of Ca^2+^ signals for Th2 and Th17 differentiation, respectively, was described before.

In murine T cells, the calcium ionophore ionomycin alone was shown to induce IL-4 production in a p38 and NFAT-dependent manner [54]. Therefore, the characteristic Th2 cytokines IL-4, IL-5 and IL-13 [55] could also be induced by thapsigargin and could be investigated in future studies. Our experiments have shown that p38 phosphorylation is enhanced by the combined stimulation of thapsigargin and pyrithione, suggesting that p38-dependent IL-4 expression [54] might also be synergistically affected by Zn^2+^ signaling.

In the differentiation of Th17 cells, the store-operated calcium entry increased the development of a pathogenic Th17 phenotype [56]. Blocking the pore subunit of CRAC channels Orai1 impaired Th17 differentiation more than Th1 and Th2 cell differentiation [57]. However, further studies demonstrated an increased Th17 differentiation with low strength T-cell stimulation and a reduction of Th17 when a Ca^2+^ ionophore was added [58]. Thus, Ca^2+^ signaling regulates the differentiation into T17 cells, however in our experiments the induction of Th17 cells was not observed. This might be due the missing stimulation via the TCR or a low threshold Ca^2+^ signal via thapsigargin. Additionally, Zn^2+^ supplementation was shown to decrease the amount of Th17 cells, so no enhancing effect of Zn^2+^ and Ca^2+^ signals was expected [59, 60].

The results of this study reinforce the relevance of Zn^2+^ signaling in addition to the well-known Ca^2+^ signals and differentiate the effects in activation and differentiation of T cells. This is of great importance for understanding the basic mechanisms of T-cell fate.

In summary, we have demonstrated different levels of Zn^2+^ and Ca^2+^ action. Zn^2+^ signals are essential for complementing Ca^2+^ signals in the activation of T cells and, in particular, Th1 differentiation, which is important for defense against intracellular pathogens.

## Materials and methods

### Isolation of peripheral blood mononuclear cells (PBMC)

After obtaining informed consent and explaining the nature and potential consequences of the studies, human venous blood was drawn from healthy volunteer donors, anticoagulated with sodium heparin (B. Braun, Melsungen, Germany) and diluted 1:2 in PBS (Sigma-Aldrich, Steinheim, Germany). PBMC were isolated from whole blood using Lymphocytes Separation Media, 1.077 g/ml (Capricorn Scientific, Ebsdorfergrund, Germany). Isolated PBMC were washed in PBS and adjusted to a final concentration of 1 × 10^6^ cells/mL in culture medium with or without Zn^2+^. The zinc-adequate (ZA) culture medium consisted of RPMI-1640 (Sigma-Aldrich) supplemented with 10% heat-inactivated fetal calf serum (FCS) “Low Endotoxin” (Bio&Sell, Feucht, Germany) 2 mM l-glutamine, 100 U/mL potassium penicillin and 100 U/mL streptomycin sulfate (all from Sigma-Aldrich). To incubate cells in medium without Zn^2+^, the described medium was treated for 1 h with Chelex^®^ 100 sodium form (Sigma-Aldrich) to chelate all divalent cations. Afterwards, 500 µM CaCl_2_ and 400 µM MgCl_2_ (both from Merck, Darmstadt, Germany) were reconstituted and the pH was adjusted back to the level of the culture medium (pH 7.4). Finally, the Zn^2+^-deficient (ZD) medium was sterile filtered. The Zn^2+^ depletion was always confirmed by atomic absorption spectrometry using an AAnalyst 800 (Perkin-Elmer, Waltham, USA). For experiments with Zn^2+^-reconstituted medium (ZR), the Chelex^®^-treated medium was measured by inductively coupled plasma mass spectrometry (ICP-MSMS) (Agilent 8900 ICP-MSMS, Agilent Technologies, Waldbronn, Germany). The concentration of the metal ions Zn, Ca, Mg, Cu, Fe and Mn were measured in samples that were diluted with nitrogenic acid, using rhodium as an internal standard (Table S1). To obtain a ZR-medium all removed metal ions were reconstituted accordingly and the ZR-medium was measured again by ICP-MS.

### T-cell isolation

Naïve CD4^+^ T cells were isolated using the MojoSort™ Human CD4 Naïve T Cell Isolation Kit (BioLegend, San Diego, CA, USA) according to the manufacturer’s protocol. For isolation, PBS with 0.5% bovine serum albumin (AppliChem, Darmstadt, Germany), and 2 mM EDTA (Sigma-Aldrich) were used.

### PBMC stimulation

PBMC were adjusted to 1 × 10^6^ cells/mL in normal (ZA) or Chelex-treated Zn^2+^-deficient (ZD) medium. Cells were stimulated with the indicated concentration of sodium pyrithione and thapsigargin and incubated at 37 °C (both from Sigma-Aldrich). SNOC (generated as described before [61]) was preincubated for 30 min at 37 °C and then additionally stimulated with thapsigargin. The time of incubation after stimulation is indicated in each figure legend. In general, RNA was analyzed 3 h and protein expression 48 h after stimulation. When p38 was examined, cells were preincubated at 37 °C for 30 min with 10 µM of the p38 MAPK inhibitor SB202190 (Sigma-Aldrich) or left untreated and subsequently stimulated with pyrithione and thapsigargin and incubated for additional 48 h at 37 °C.

### Labile Zn^2+^ and Ca^2+^ measurements

To measure Zn^2+^ and Ca^2+^ signals, respectively, 1–30 min after stimulation 2 × 10^6^ PBMC/mL were first stained with 1 µM FluoZin-3 AM or Fluo-4 for 30 min (both from Invitrogen by Thermo Fisher Scientific, Eugene, OR, USA). After the staining 1 × 10^6^ cells in 1 mL culture medium were stimulated with the indicated concentrations of pyrithione, thapsigargin (both from Sigma-Aldrich) or SNOC (generated as described before [61]) and incubated for 10 min or for the indicated time intervals at 37 °C. For Zn^2+^ measurements, the washed cells were then incubated with 100 µM ZnSO_4_ and 5 µM sodium pyrithione to induce a maximal fluorescence (*F*_max_), with 50 µM of the Zn^2+^ chelator *N*,*N*,*N*,*N*-tetrakis(2-pyridylmethyl)-ethylenediamine (TPEN) to induce a minimal fluorescence (*F*_min_) (all from Sigma-Aldrich) or were left untreated (*F*) and incubated for 10 min in a 37 °C water bath. Instead, for Ca^2+^ measurements cells were incubated with 2 µM A23187 (Tocris, bio-techne, Wiesbaden-Nordenstadt, Germany) to induce *F*_max_ and with 20 mM EDTA (Sigma-Aldrich) to induce *F*_min_ or were left untreated (*F*). The fluorescent intensity was measured using the FACSCalibur™ Flow Cytometer (BD Biosciences, San Jose, CA, USA) and analyzed with FlowJo™ Software version 10.8.1 (BD Biosciences). Lymphocytes were gated according to their size. Labile Zn^2+^ and Ca^2+^ were calculated using the equation $\left[{\rm{Zn}}^{2+}\right]{\rm{or}}[{\rm{Ca}}^{2+}]={K}_{\rm{D}}\times \frac{({F-F}_{\min })}{({F}_{\max }-F)}$ with a dissociation constant (*K*_D_) for FluoZin-3 AM (Zn^2+^) of 8.9 nM and for Fluo-4 (Ca^2+^) of 335 nM.

For measurements of intracellular Zn^2+^ 48 h after stimulation, 1 × 10^6^ PBMC/mL were first stimulated as described before and incubated for 48 h at 37 °C. Afterwards, the collected cells were stained with 1 µM FluoZin-3 AM for 30 min, and subsequently *F*_min_, *F*_max_ and *F* were induced in each sample and measured and analyzed by flow cytometry as described above.

### Flow cytometry

1 × 10^6^ cells in PBS + 1% FCS were incubated with the antibodies CD3-FITC (Cat. 345763) and CD69-APC (Cat. 555533) or the respective isotype controls IgG1, κ-FITC (Cat. 555748) and IgG1, κ-APC (Cat. 555751) (antibodies from BD Biosciences) for 20 min at room temperature in the dark. Afterwards, cells were washed and measured by flow cytometry using FACSCalibur™ Flow Cytometer (BD Biosciences) and analyzed with FlowJo™ Software version 10.8.1 (BD Biosciences). Lymphocytes were gated according to their size.

### ELISA

For the quantification of IL-2, IFN-γ, IL-10 and IL-17 the supernatants were collected after 48 h of incubation and stored at −20 °C. Samples were diluted and IL-2, IFN-γ, IL-10 (all from BD Biosciences) or IL-17 ELISA (R&D Systems, Minneapolis, MN, USA) were performed according to the manufacturer’s protocol. The detection limits were as follows: 7.8 pg/mL (IL-2); 4.7 pg/mL (IFN-γ); 7.8 pg/mL (IL-10) and 15.6 pg/mL (IL-17). Values below the detection limit were substituted a value just below the detection limit. Samples were determined in duplicates and absorption was detected using the Spark plate reader (Tecan, Männedorf, Switzerland).

### Quantitative real-time polymerase chain reaction (qPCR)

2 × 10^6^ PBMC were lysed in 1 mL TRIzol® Reagent (Ambion/Life Technologies, Darmstadt, Germany) and stored at −80 °C. mRNA was extracted according to the manufacturer’s protocol and the mRNA concentration was determined by Nanodrop 2000 (Thermo Fisher Scientific, Waltham, MA, USA). cDNA synthesis was performed using the qScript^TM^ cDNA Synthese Kit (Quantabio, Beverly, MA, USA) and was diluted 1:2.5 in UV-irradiated H_2_O.

qPCR was performed with PowerSYBR^®^ Green PCR Master Mix (Applied Biosystems by Thermo Fisher Scientific, Woolston, UK) and 100 nM of the respective forward and reverse primer. The following primer were used: IFN-γ [49], PBGD [49], IRF-1 [47], KLF-10 [47], Zip1-2 [34], Zip3 [62]; Zip4-12 [63], Zip13 [63], Zip14 [64], ZnT1-9 [65], ZnT10 [34]. The following amplification cycles were used: 1 × 10 min at 95 °C, 40 × 15 s at 95 °C and 60 s at 60 °C, 62 °C (Zip13) or 65 °C (ZnT10). Afterwards, a melting curve was performed. Data were analyzed by ${2}^{-\Delta \Delta {C}_{T}}$ method [66] and if the mean C_T_ of the PCR product was >35, the corresponding mRNA expression was excluded as not detectable (n.d.).

### Western blot

2 × 10^6^ cells/sample were resuspended in 100 µL sampling buffer (65 mM Tris-HCl [pH 6.8], 2% SDS, 1 mM Na_3_VO_4_, 26% glycerol, 1% β-mercaptoethanol and 0.01% bromphenol blue), lysed by sonification using Vibra Cell sonicator (Sonics & Materials, Newtown, CT, USA) and heated for 5 min at 95 °C. The sample volume used was defined by the protein concentration, which was determined by Bio-Rad Protein Assay Dye Reagent Concentrate (Bio-Rad Laboratories, Inc., Hercules, CA, USA). A color prestained protein standard (New England BioLabs, Frankfurt a.M., Germany) and the samples were run on a 10% polyacrylamide gel at 170 V and blotted onto nitrocellulose membranes at 100 V. Loading of the gel and protein transfer was confirmed by Ponceau S staining (AppliChem). Membranes were blocked for 1 h in TBS-T (20 mM Tris [pH 7.6],137 mM NaCl and 0.1% Tween-20), containing 5% fat-free dry milk. Subsequently, membranes were incubated overnight at 4 °C in primary antibodies which were diluted 1:1000 in TBS-T and 5% bovine serum albumin (p38 MAPK (Cat. 9212 S), phospho-p38 MAPK (Thr180/Tyr182) (Cat. 9215 S), T-bet (Cat. 13232), GATA-3 (Cat. 5852), FoxP3 (Cat. 12632), β-actin (Cat. 4967 S) (all from Cell Signaling Technology, Danvers, MA, USA), ROR*γ*(t) (Cat. PA5-86733, Invitrogen by Thermo Fisher Scientific)). The secondary antibody HRP-linked anti-rabbit IgG (Cat. 7074, Cell Signaling Technology) was diluted 1:2000 in TBS-T and 5% fat-free dry milk and membranes were incubated at room temperature for 3 h. Band density was determined using Westar Antares (Cyanagen, Bologna, Italy) and LAS-3000 (Fujifilm) and was analyzed by ImageJ (National Institutes of Health, Bethesda, MD, USA). Original western blots are available as supplementary information.

### Statistical analysis

Data were statistically analyzed using GraphPad Prism (version 8.0.1). All data were tested for outliers by the ROUT method, which were removed accordingly, and tested for normality with the Shapiro–Wilk test (*n* < 8) or with the Anderson–Darling test (*n* > 8). Accordingly, parametric or non-parametric two-sided tests were used. Sphericity was not assumed and the Geisser–Greenhouse correction was used. The corresponding tests and the number of sample units are indicated in the figure legends. Different N are PBMC from individual donors whereas experiments were performed with up to three individual donors on 1 day. Experiments were performed with at least three individual donors. If experiments were significant or clearly not significant, experiments were stopped. In the case of statistical trends, a simple power analysis was performed and the number of experiments was increased.

Data are presented as mean + SEM. When the mean of each column was compared with the mean of every other column, letters were used to clearly show significant differences. Significantly different results (*p* < 0.05) have no common identification letter. For all other tests, significances are indicated as *p < 0.05; **p < 0.01; ***p < 0.001.

### Supplementary information

Complete supplementary material

Original Data

## Data Availability

All data generated or analyzed during this study are included in this published article and detailed data are available from the corresponding author on reasonable request. Original western blots were uploaded as Supplementary Material.
