# Supplementary material for: Ca2+ signals are essential for T-cell proliferation, while Zn2+ signals are necessary for T helper cell 1 differentiation
Source: Cell Death Discov. 2024 Jul 24;10:336. doi: 10.1038/s41420-024-02104-1 (PMC11266428; doi:10.1038/s41420-024-02104-1)
Supplement: Supplementary file 1 — Complete supplementary material [file 41420_2024_2104_MOESM1_ESM.docx]

**Supplementary Information**


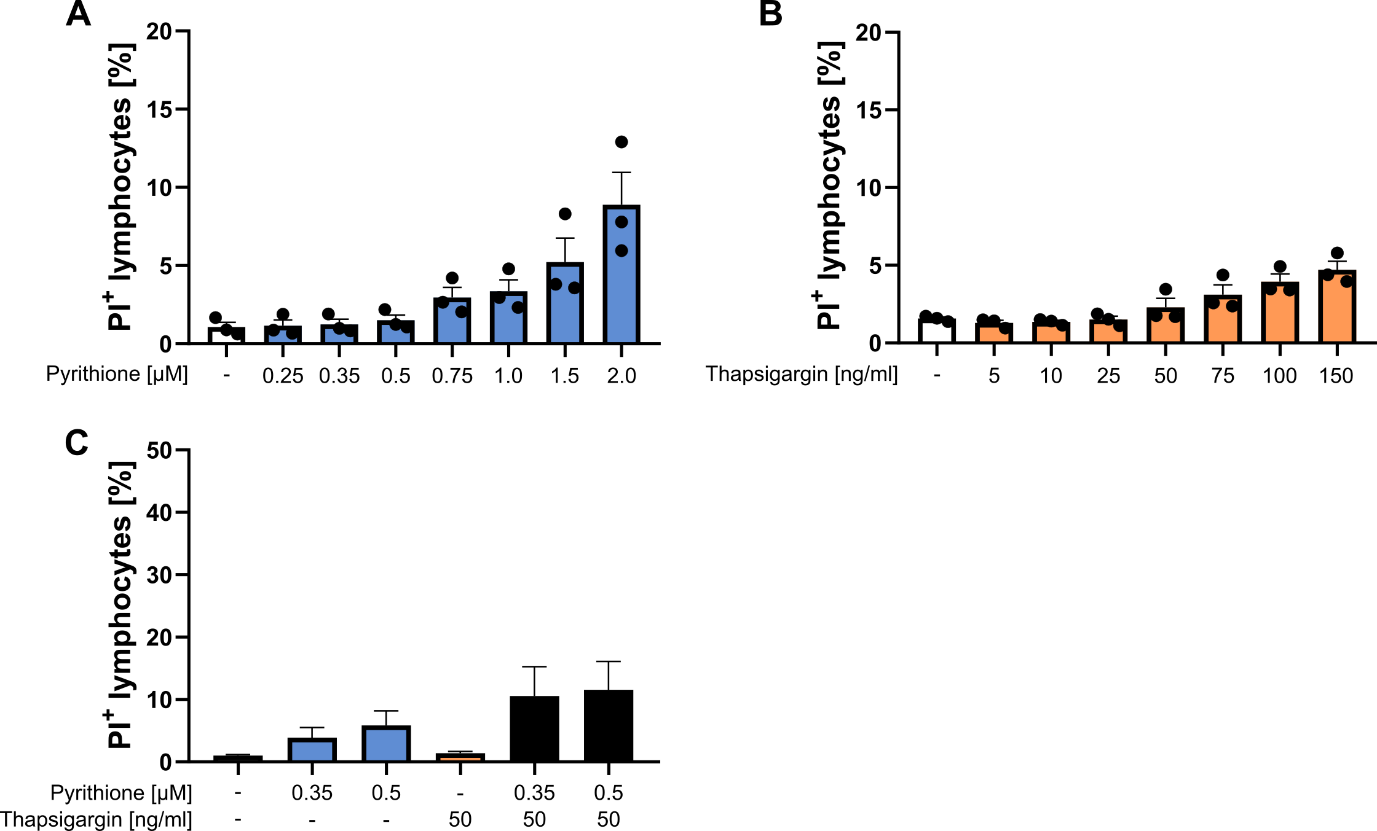


Fig. S 1. Toxicity of pyrithione and thapsigargin. 1 × 10^6^/ml PBMC were stimulated with (A) pyrithione, (B) thapsigargin or (C) with the combination of both at the indicated concentrations and incubated for 48 h at 37 °C. Afterwards, cells were stained with propidium iodide (PI) to stain dead cells. Cells were investigated with flow cytometry and PI^+^ cells are shown for gated lymphocytes. Data are presented as mean + SEM with n = 3 (A, B) and n = 5 (C) experiments. Statistical significance to the control (-) was determined by one-way ANOVA with Dunnett's multiple comparisons test. No significant differences were found.


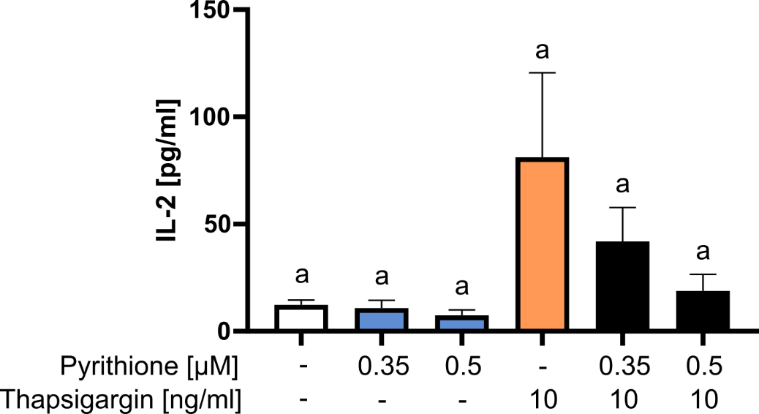


Fig. S 2. Interleukin (IL)-2 expression with low thapsigargin concentration. 1 × 10^6^/ml PBMC were stimulated with pyrithione and thapsigargin at the indicated concentration and incubated for 48 h at 37 °C. Afterwards, the IL-2 concentration in the supernatant was determined by ELISA. Data are presented as mean + SEM with n = 5 experiments. Data of the experiments without thapsigargin are also included in Fig. 3A. Statistical significance was determined by one-way ANOVA with Tukey's multiple comparisons test. No significant differences were found.

Fig. S 3. Interferon (IFN)-γ expression in naïve CD4^+^ T cells. 1 × 10^6^/ml isolated naïve CD4^+^ T cells were stimulated with or without the indicated concentrations of pyrithione and thapsigargin and incubated for 48 h. Afterwards, IFN-γ was measured in the supernatant by ELISA. Data are presented as mean + SEM with n = 6 experiments. Statistical significance was determined by paired t-test (* p <  0.05).


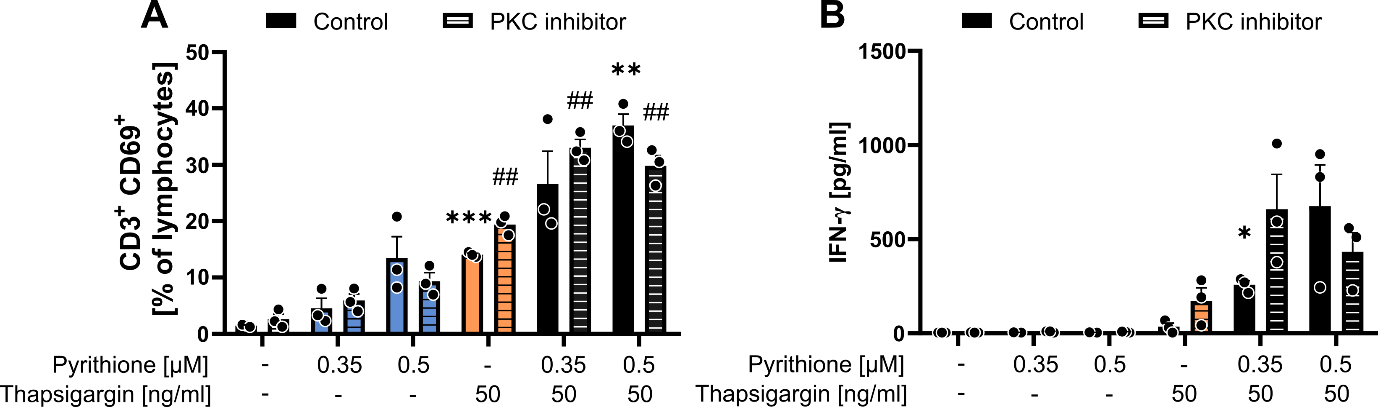


Fig. S 4. Inhibition of protein kinase C activity. 1 × 10^6^/ml PBMC were preincubated with or without 5 µM of the protein kinase C (PKC) inhibitor bisindolylmaleimide II (Sigma-Aldrich, Steinheim, Germany) for 60 min at 37°C. Subsequently, cells were stimulated with the indicated concentrations of pyrithione and thapsigargin and incubated for 48 h at 37°C. (A) After 48 h, activated T cells (CD3+CD69+) were determined by flow cytometry and (B) IFN-γ production was determined by ELISA. Data are presented as mean + SEM with n = 3 experiments. Statistical significance was determined by two-way ANOVA with Dunnett’s multiple comparisons test (** p < 0.01, *** p < 0.001 to unstimulated control; ## p < 0.01 to unstimulated BIS II).


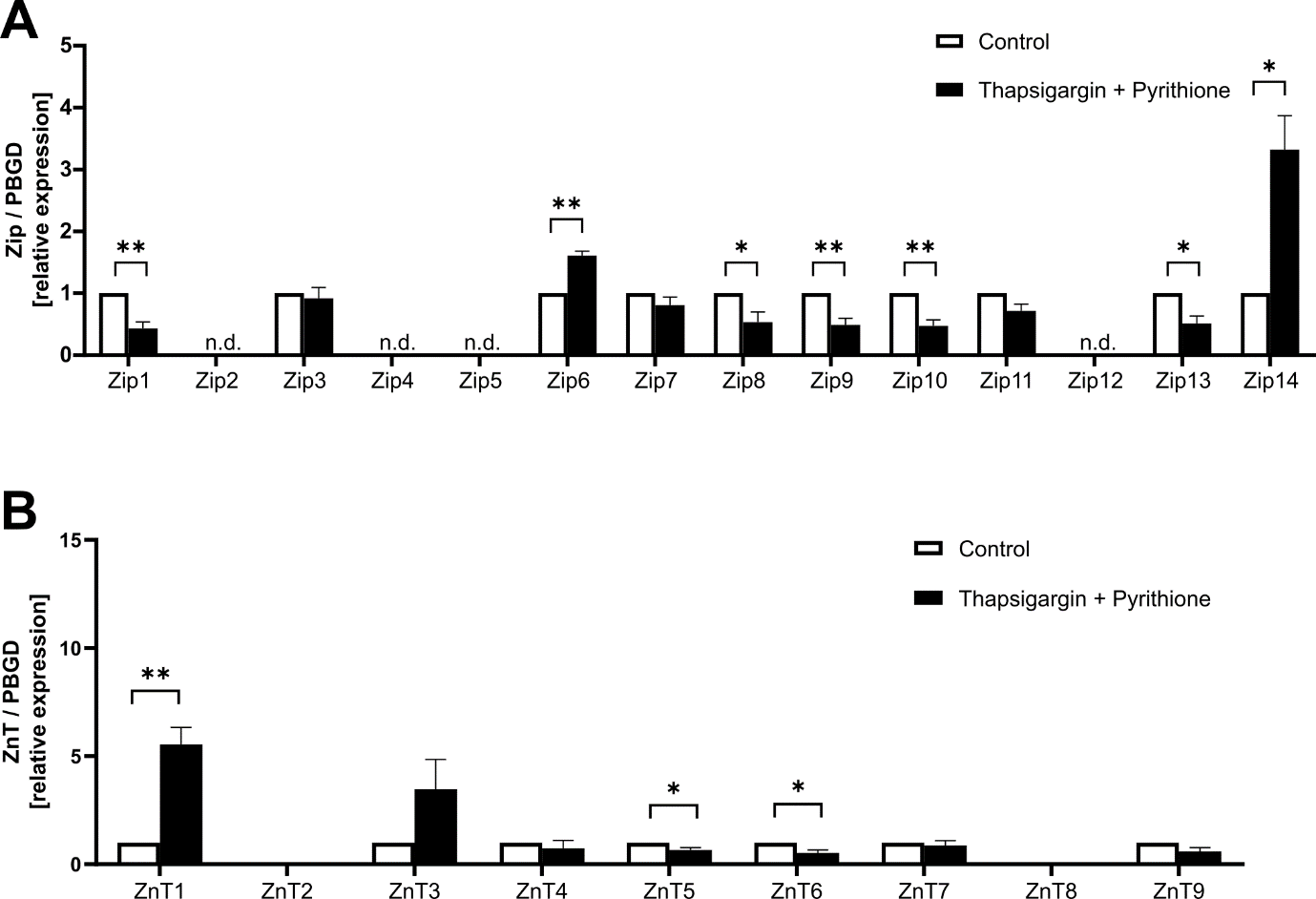


Fig. S 5. Zn^2+^ transporter expression after thapsigargin and pyrithione stimulation. PBMC were left untreated, stimulated with 50 ng/ml thapsigargin and 50 µM pyrithione. (A) 3 h after stimulation the mRNA expression of the Zn^2+^ importer and (B) the Zn^2+^ exporter were analyzed by qPCR and relative expression was determined by $\mathbf{2}^{\boldsymbol{-\Delta\Delta}\boldsymbol{C}_{\boldsymbol{T}}}$ method. The expression of some transporter was not detectable (n.d.). Data are presented as mean + SEM with n = 5 (A, B) experiments. Statistical significance was determined by paired t-test (* p < 0.05; ** p < 0.01).

Fig. S 6. Extracellular Zn^2+^ is needed for thapsigargin induced IFN-γ expression. 1 × 10^6^/ml PBMC were stimulated with 50 ng/ml thapsigargin in Zn^2+^-deficient (ZDR) or Zn^2+^-reconstituted (ZR) culture medium. The respective media are described in Table S 1. After 48 h of incubation, IFN-γ was measured in the supernatant by ELISA. Data are presented as mean + SEM (n = 9) of responsive donors. Statistical significance was determined by paired t-test (* p <  0.05).

Table S 1. Culture media. The concentrations of the respective metal ions were measured by inductively coupled plasma mass spectrometry (ICP-MSMS) in zinc-adequate (ZA) and Chelex^®^-treated (Chelex) culture medium. In the respective experiments in the main manuscript the Chelex medium was reconstituted with Ca and Mg to obtain a zinc-deficient (ZD) medium since Mayer et al (2014) have shown that mainly Zn, Ca and Mg are removed by Chelex treatment. In Fig. S 6 we have investigated whether Zn supplementation rescues the effects of ZD. To exclude effects by other metal ions, not only Ca and Mg but also Cu, Fe and Mn were reconstituted in the Chelex-treated medium to obtain a zinc-deficient reconstituted medium (ZDR) or, as a rescue condition, Zn was additionally added to obtain a fully reconstituted medium (ZR). The ZR medium was again measured by ICP‑MSMS. NA: not applicable; +: reconstituted, –: not reconstituted.

| **Scheme of reconstitution** | | | | | |  | **ICP-MS measurements** | | |
| --- | --- | --- | --- | --- | --- | --- | --- | --- | --- |
|  | **ZA** | **Chelex** | **ZD** | **ZDR** | **ZR** |  | **ZA** | **Chelex** | **ZR** |
| Zn | NA | – | – | – | + | Zn [µg/L] | 277.6 | 43.1 | 253.7 |
| Ca | NA | – | + | + | + | Ca [mg/L] | 26.4 | 0.2 | 24.6 |
| Mg | NA | – | + | + | + | Mg [mg/L] | 12.3 | 0.0 | 12.9 |
| Cu | NA | – | – | + | + | Cu [µg/L] | 12.6 | 9.9 | 11.8 |
| Fe | NA | – | – | + | + | Fe [µg/L] | 199.4 | 189.0 | 211.3 |
| Mn | NA | – | – | + | + | Mn [µg/L] | 4.3 | 2.4 | 4.4 |

**References**

Mayer LS, Uciechowski P, Meyer S, Schwerdtle T, Rink L & Haase H. Differential impact of zinc deficiency on phagocytosis, oxidative burst, and production of pro-inflammatory cytokines by human monocytes. *Metallomics* **6**, 1288–95 (2014)
