# Supplementary material for: Ca2+ signals are essential for T-cell proliferation, while Zn2+ signals are necessary for T helper cell 1 differentiation
Source: Cell Death Discov. 2024 Jul 24;10:336. doi: 10.1038/s41420-024-02104-1 (PMC11266428; doi:10.1038/s41420-024-02104-1)
Supplement: Supplementary file 2 — Original Data [file 41420_2024_2104_MOESM2_ESM.docx]

**Supplementary Information: Original Western Blots**

- β-Actin control blots were sometimes used for different figures since the same blots were used for detecting different specific factors. This is indicated in the respective blots.
- Lanes outside the frames were not used in this manuscript. These lanes represent different conditions and controls not used in this manuscript.

Figure 2D:

Lanes as described in Fig. 2D: Control | Pyr 0.35 | Pyr 0.5 | Thaps 50 | Thaps 50 + Pyr 0.35 | Thaps 50 + Pyr 0.5

|  | p38 | | phospho-p38 | |
| --- | --- | --- | --- | --- |
|  | Original molecular weight ladder | Original chemiluminescent blot | Original molecular weight ladder | Original chemiluminecent blot |
| J 01.08.2023 | 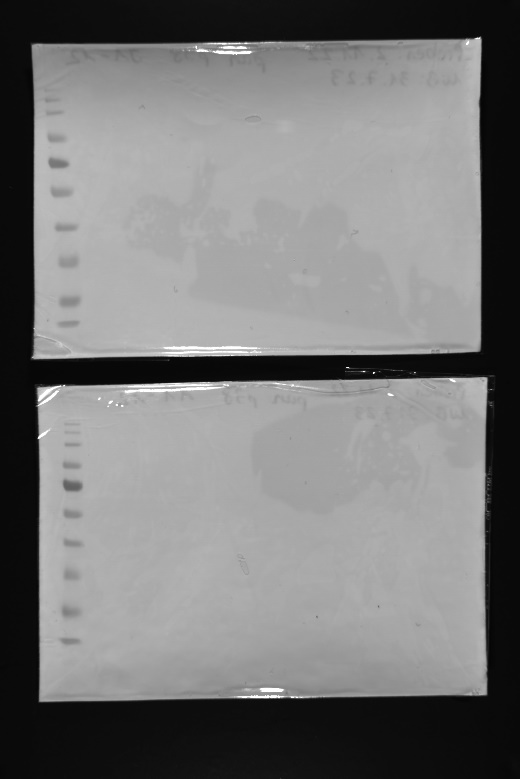 | 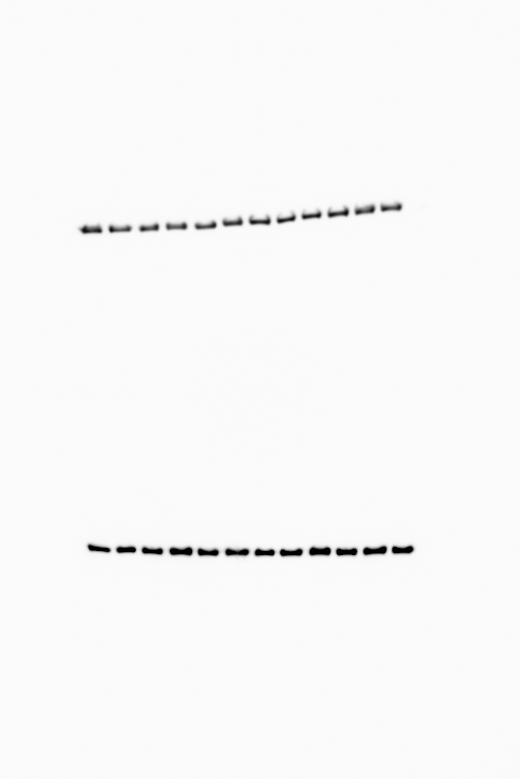 | 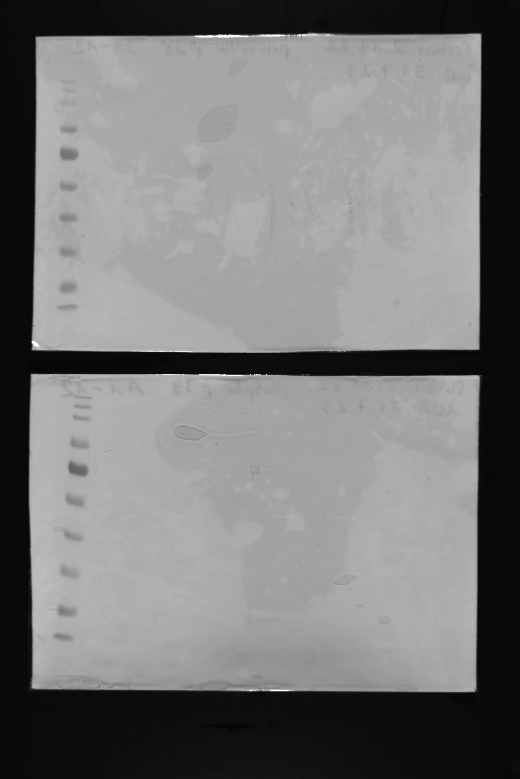 | 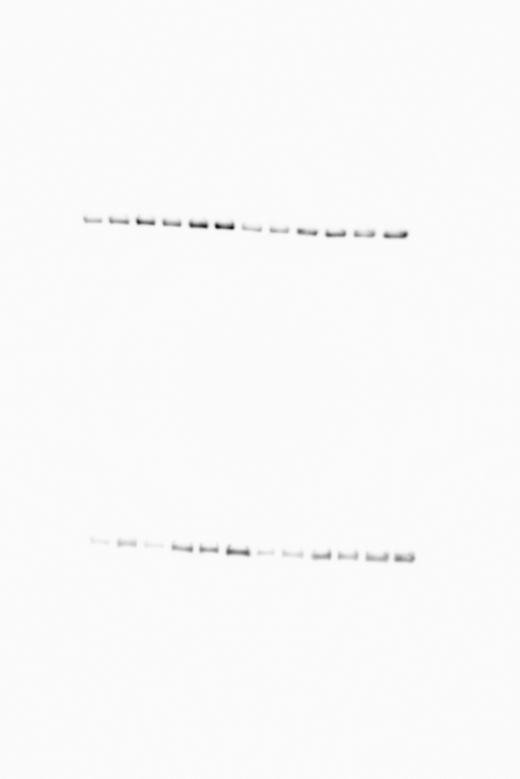 |
| A 01.08.2023 | 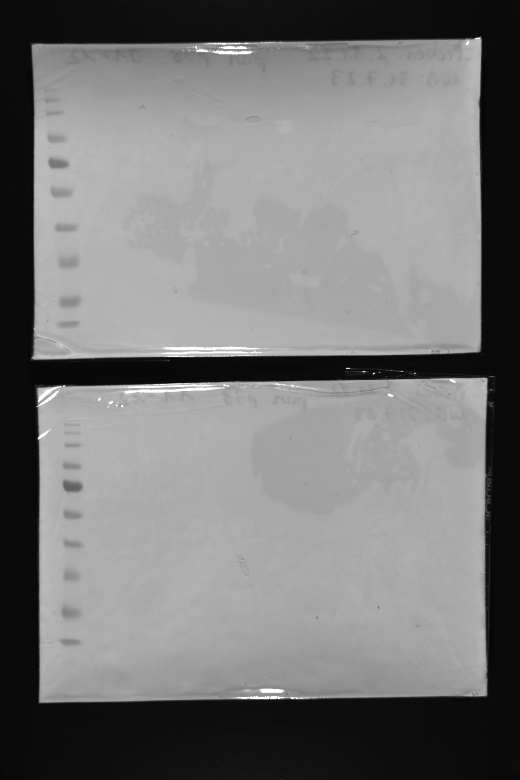 | 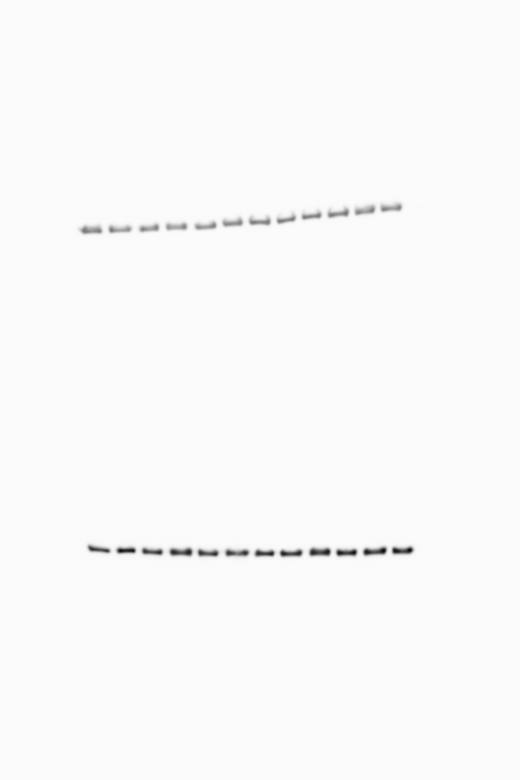 | 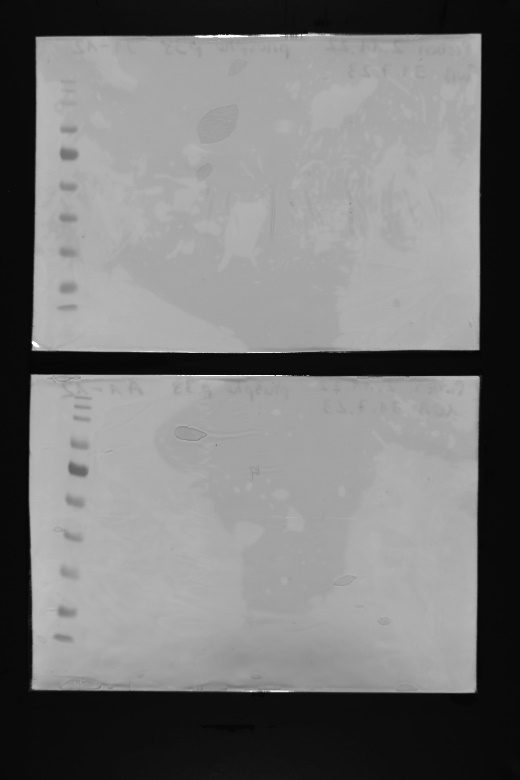 | 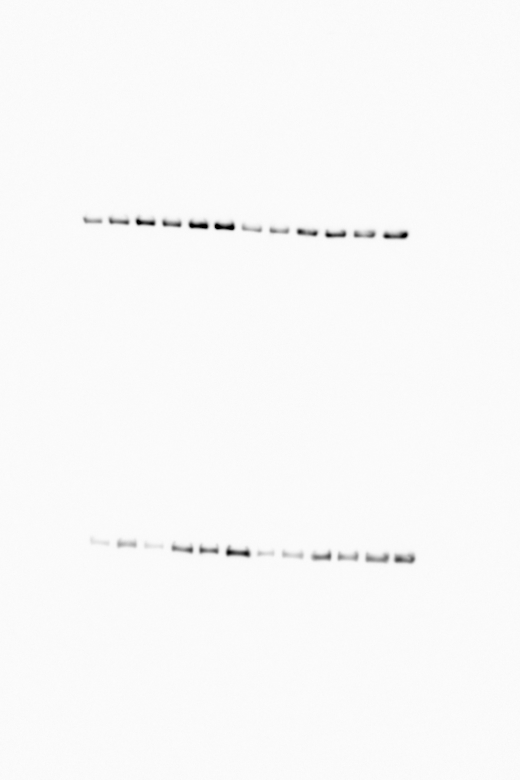 |
| J 24.08.2023 | 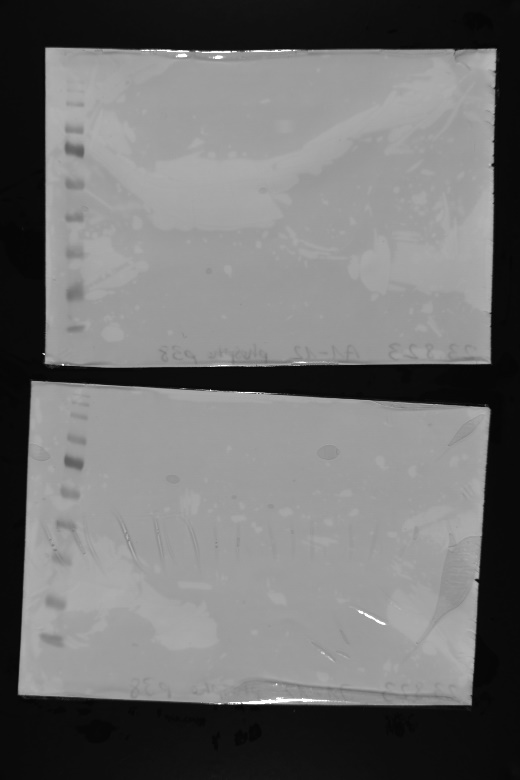 | 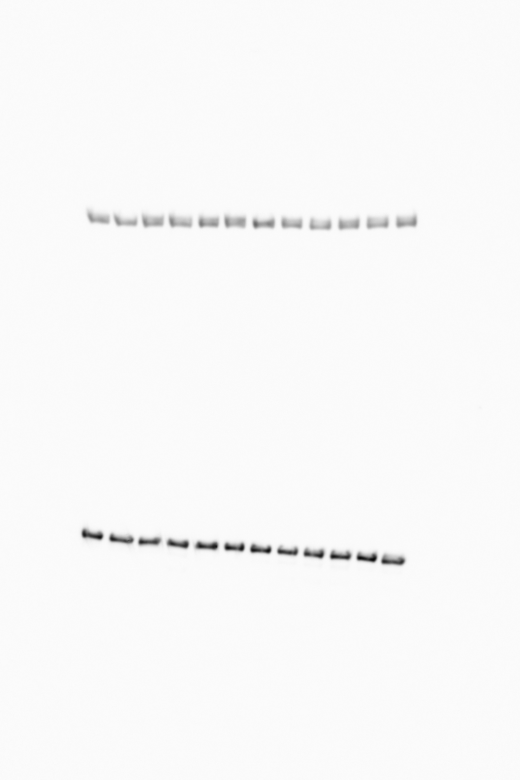 | 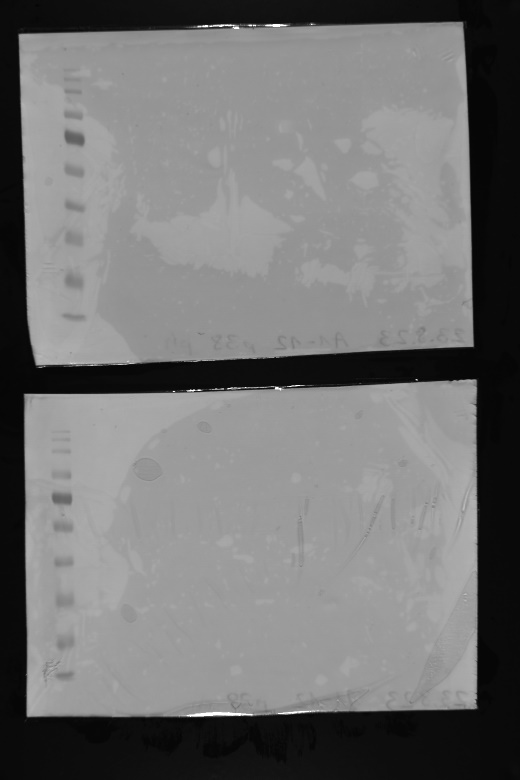 | 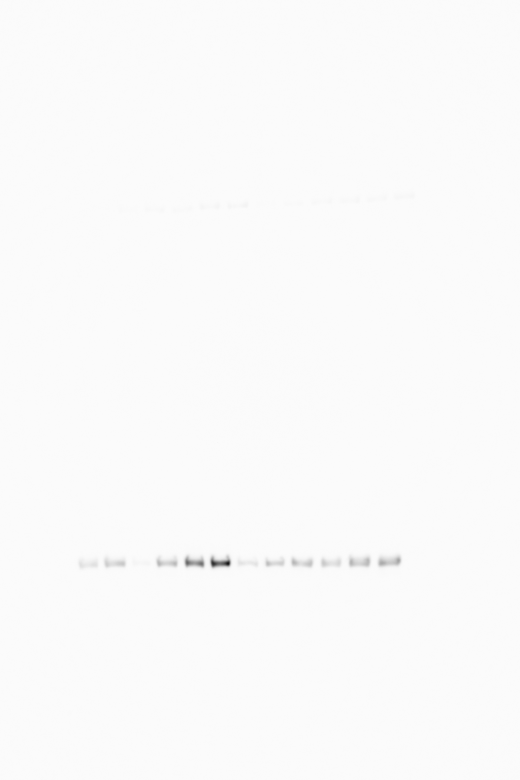 |
| T 24.08.2024 | 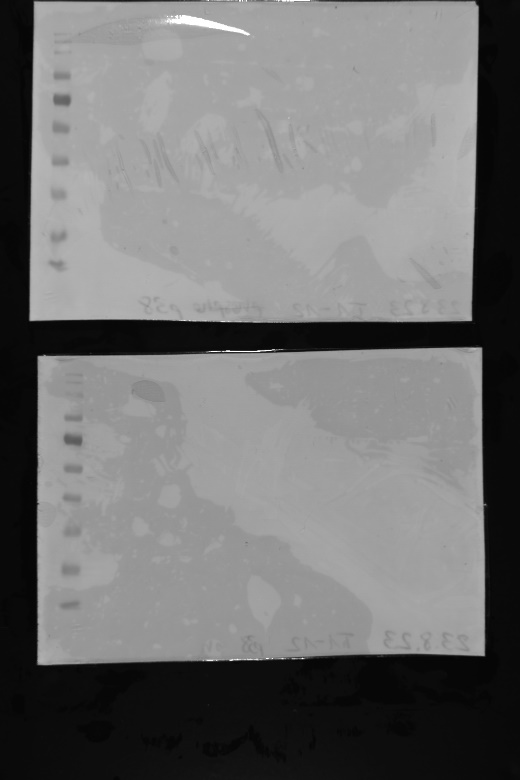 | 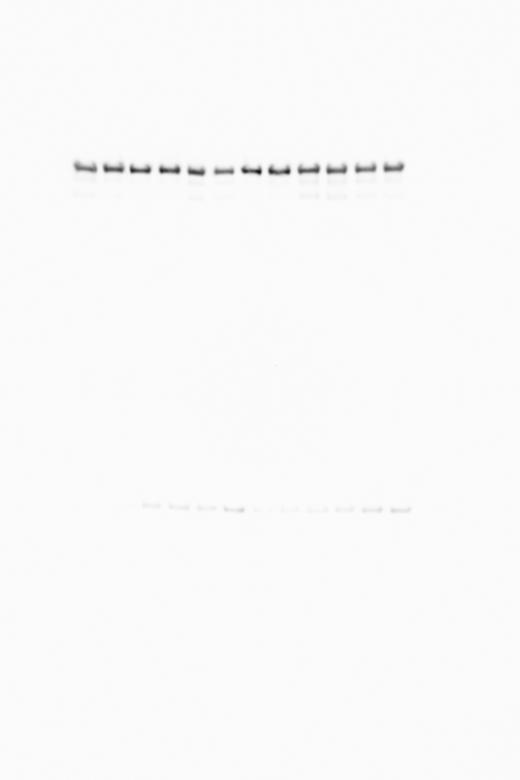 | 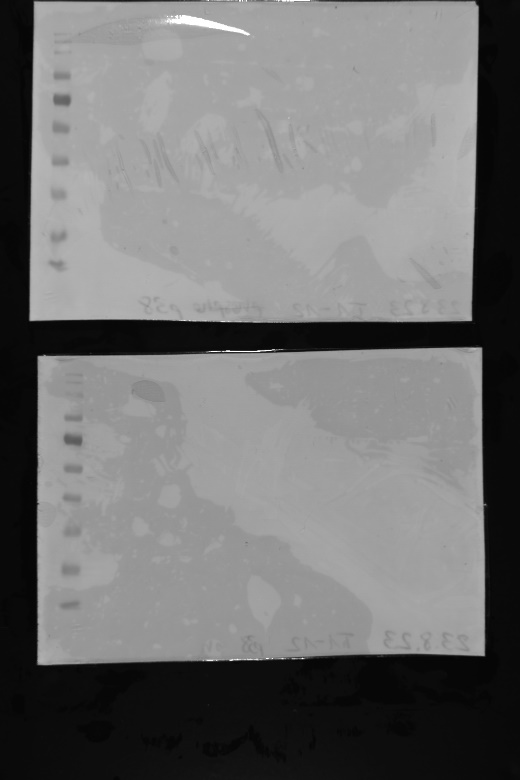 | 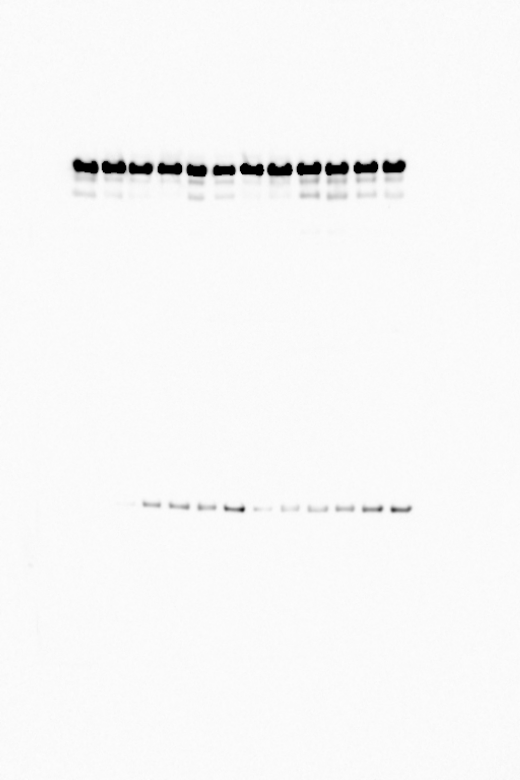 |
| A 24.08.2024 | 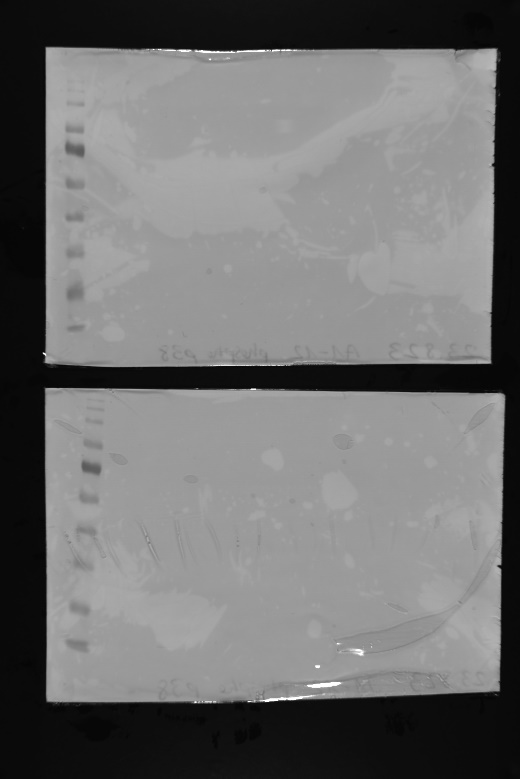 | 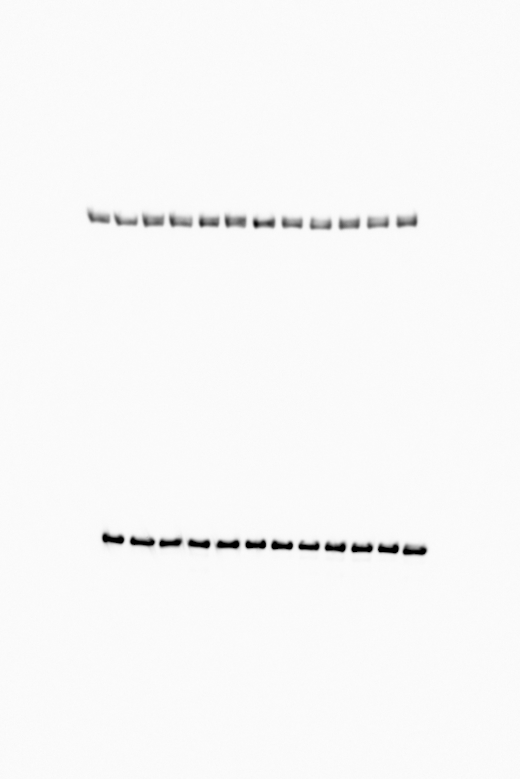 | 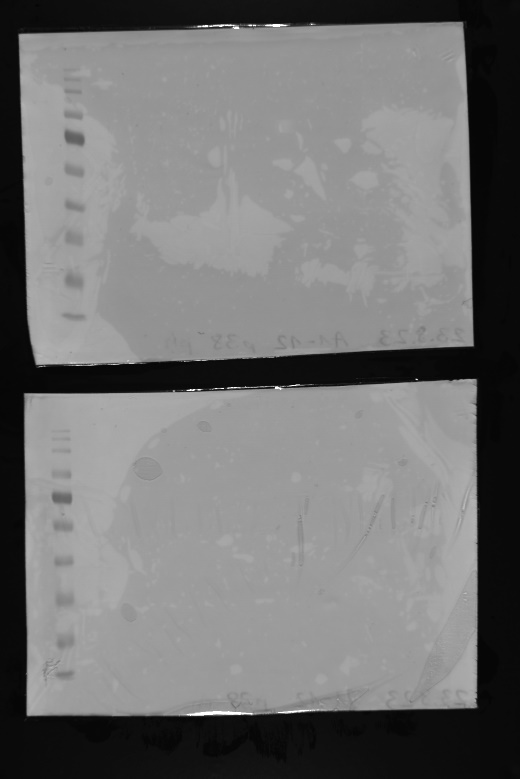 | 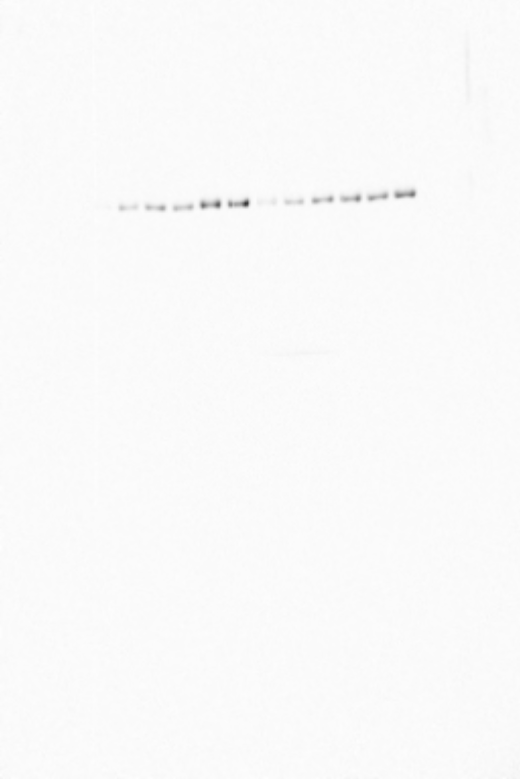 |

Figure 3D:

Lanes as described in Fig. 3D: Control | Pyr 0.35 | Pyr 0.5 | Thaps 50 | Thaps 50 + Pyr 0.35 | Thaps 50 + Pyr 0.5

|  | β−Actin | |  | T-bet | |
| --- | --- | --- | --- | --- | --- |
|  | Original molecular weight ladder | Original chemiluminescent blot |  | Original molecular weight ladder | Original chemiluminecent blot |
| M 08.10.2021 | 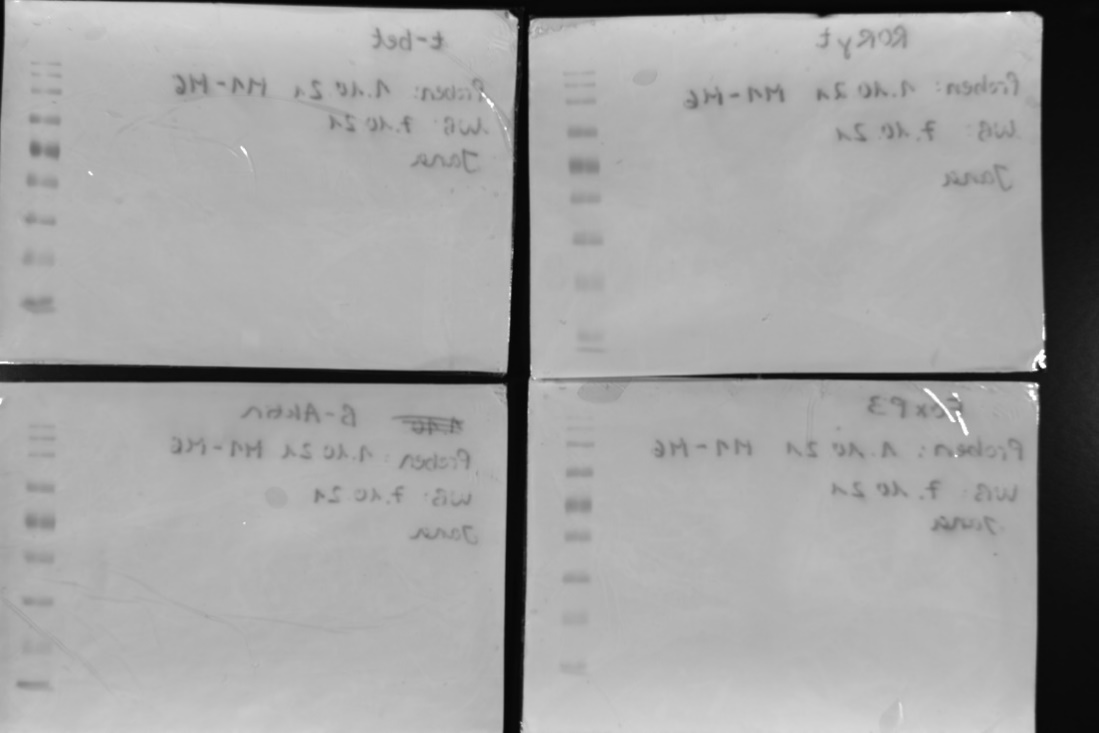 | 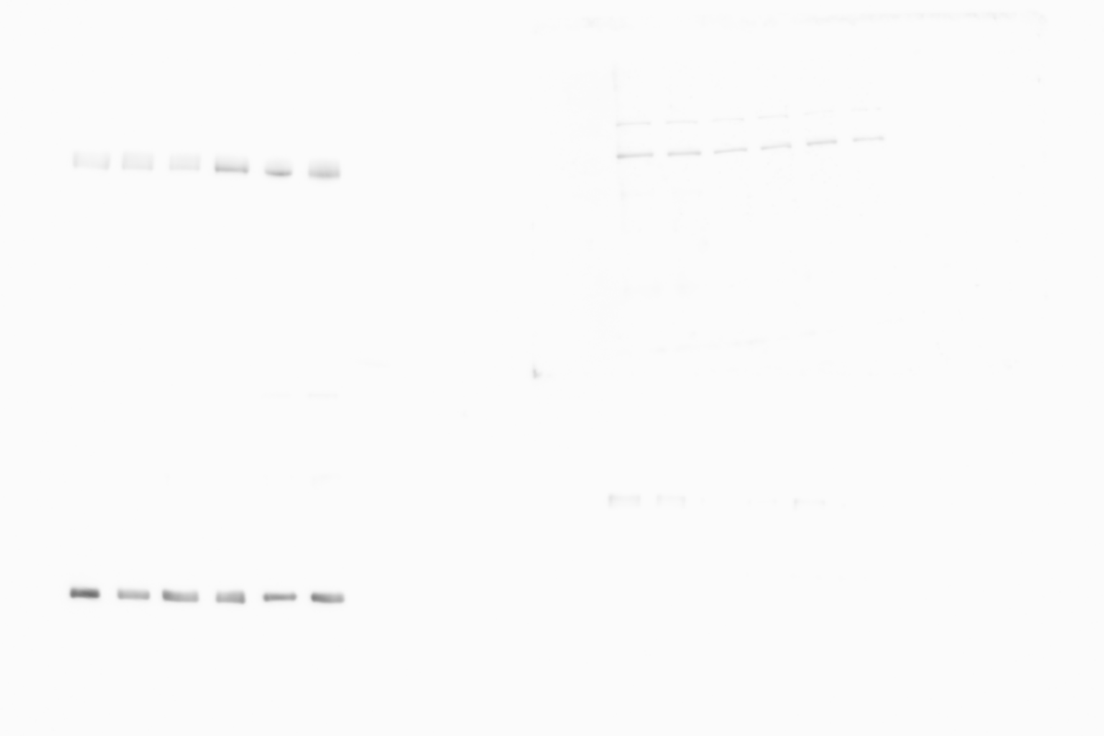 |  | 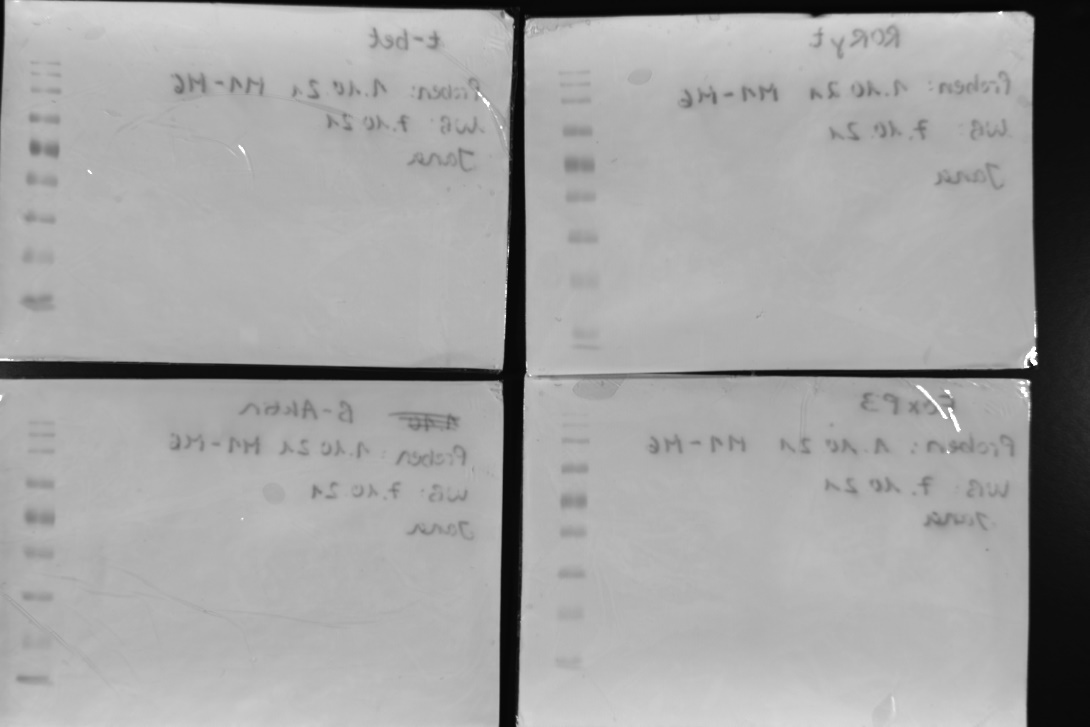 | 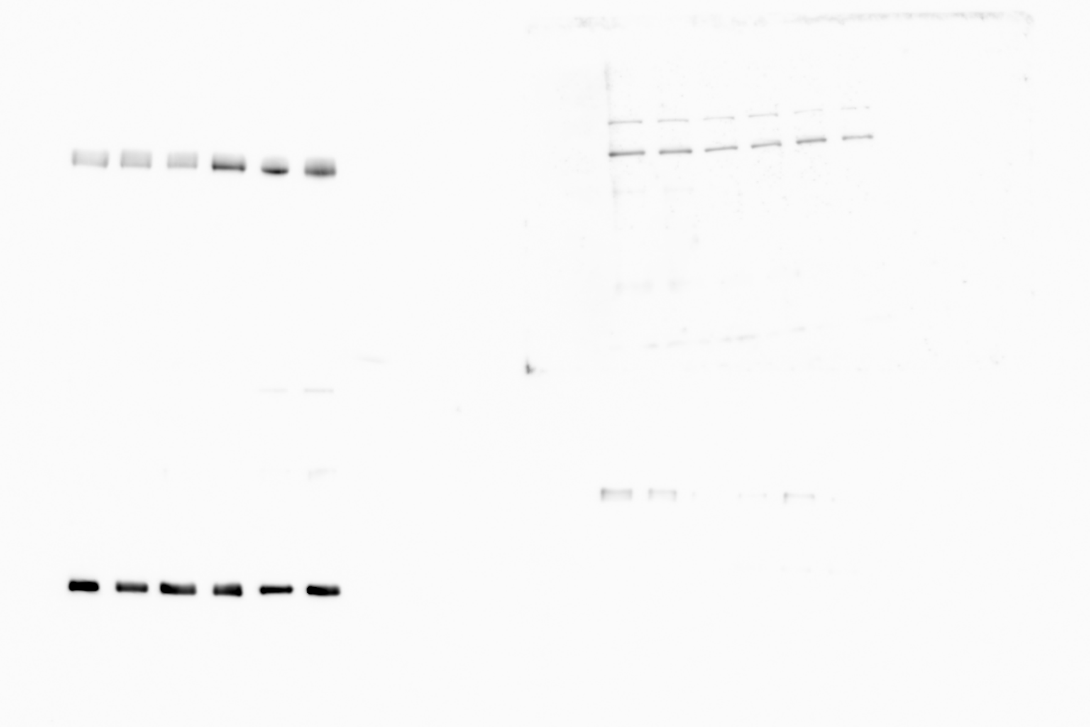 |
| A 08.10.2021 | 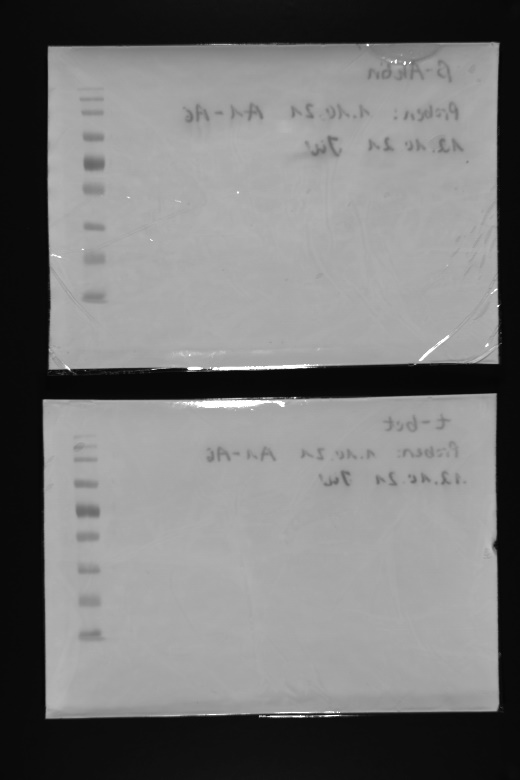 | 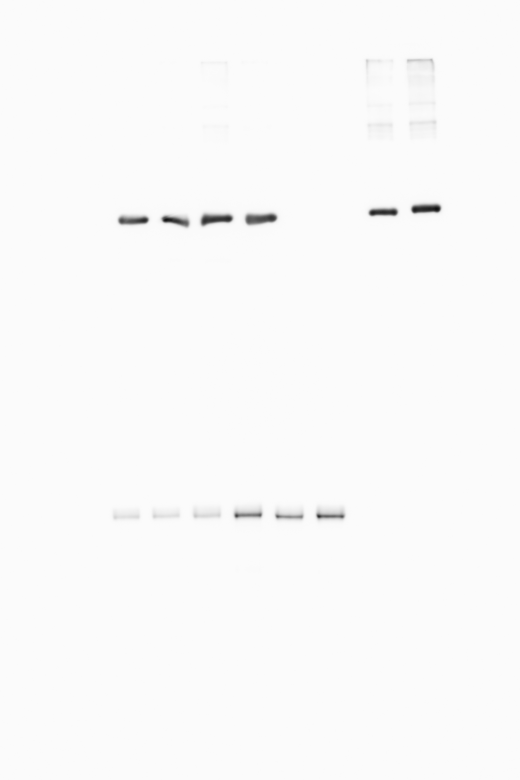 |  | 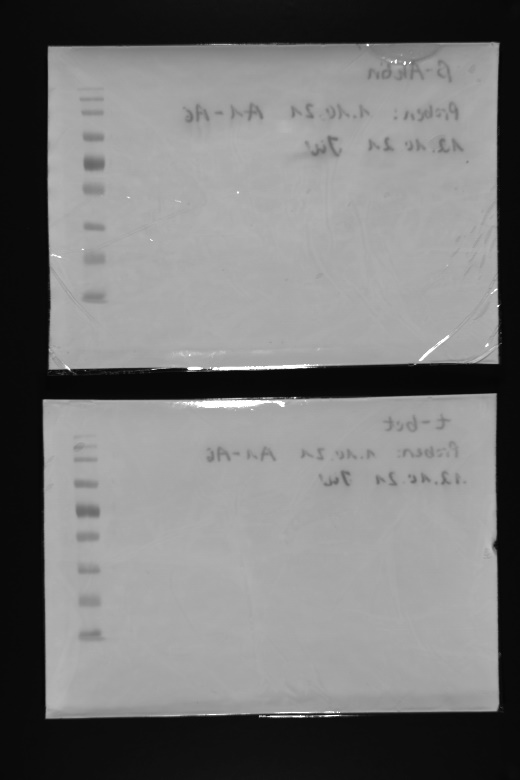 | 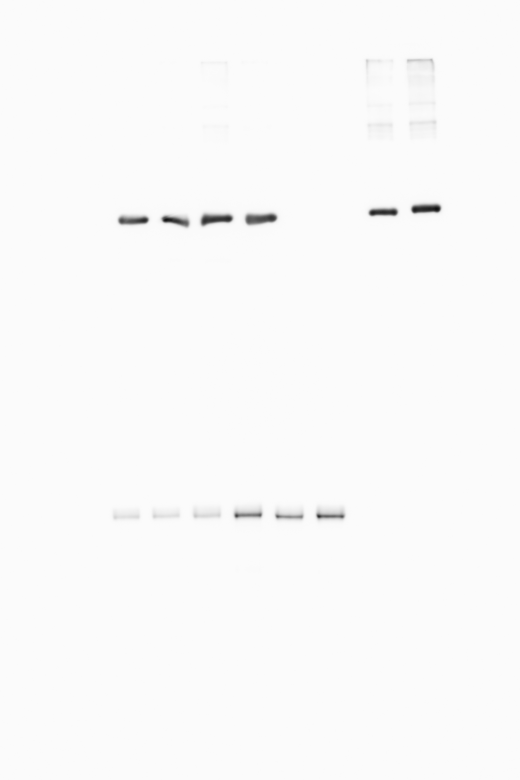 |
| K 22.10.2021 | 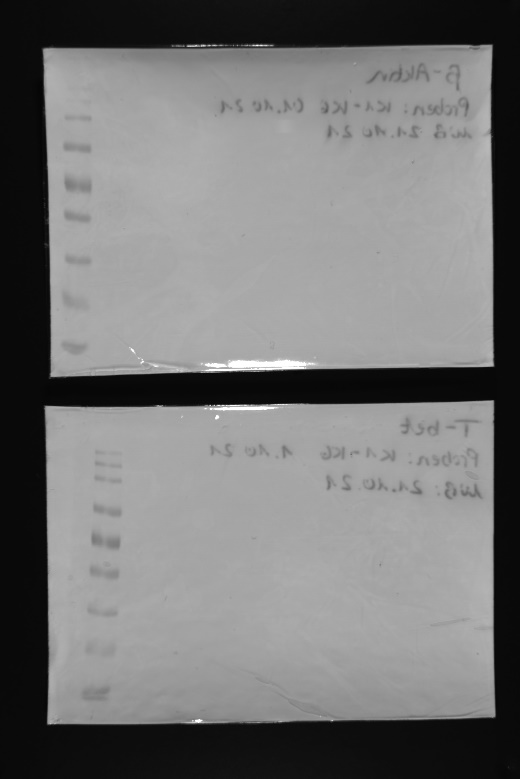 | 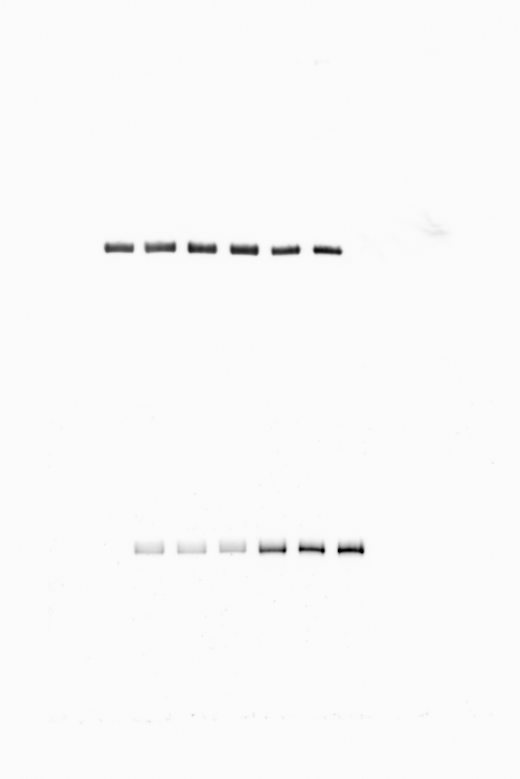 |  | 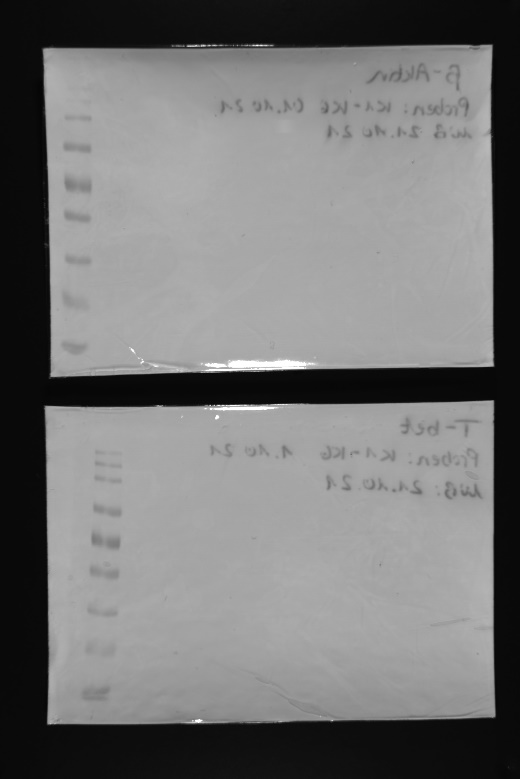 | 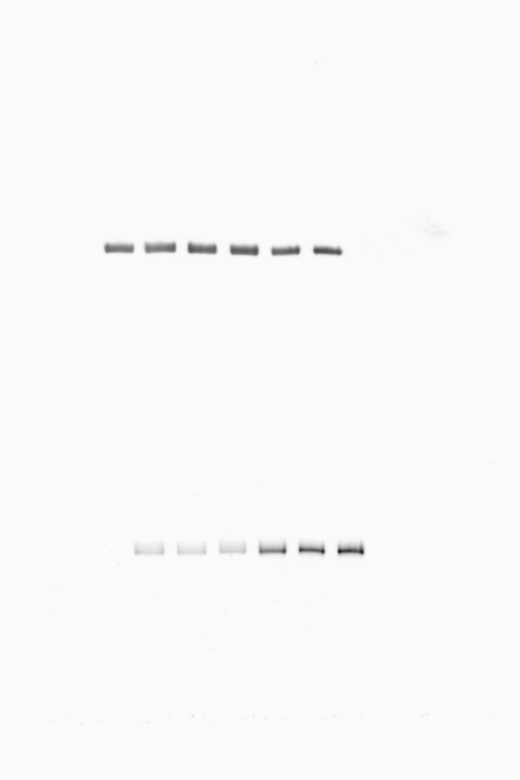 |
| B 19.11.2021 | 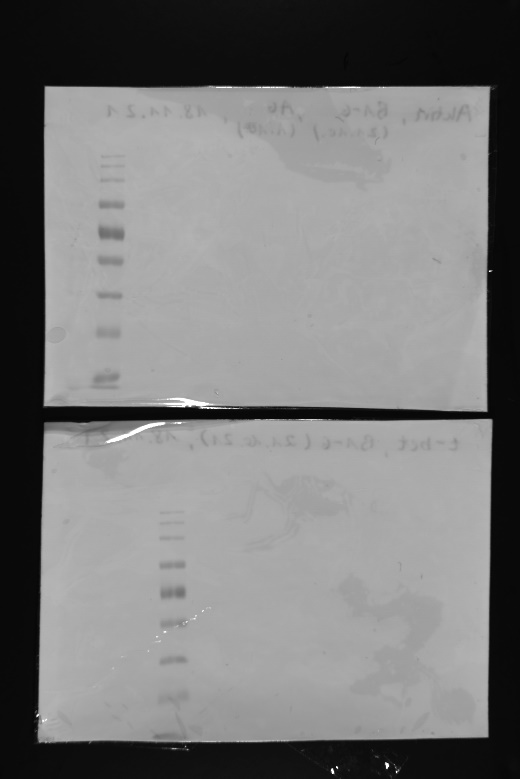 | 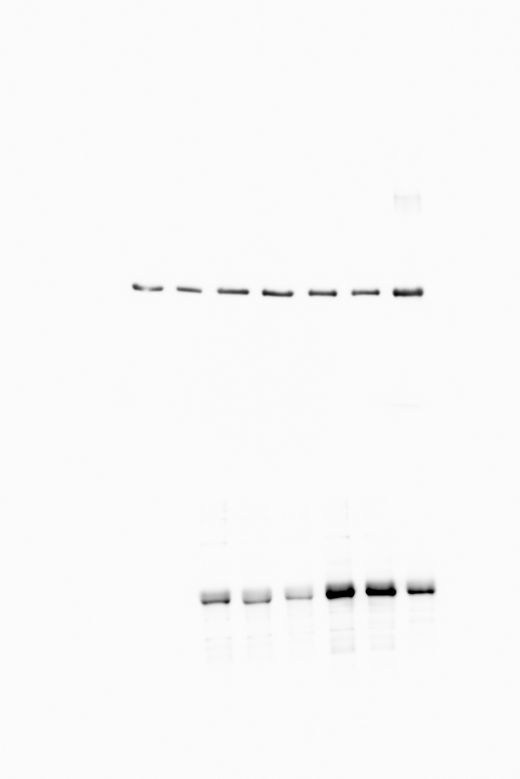 |  | 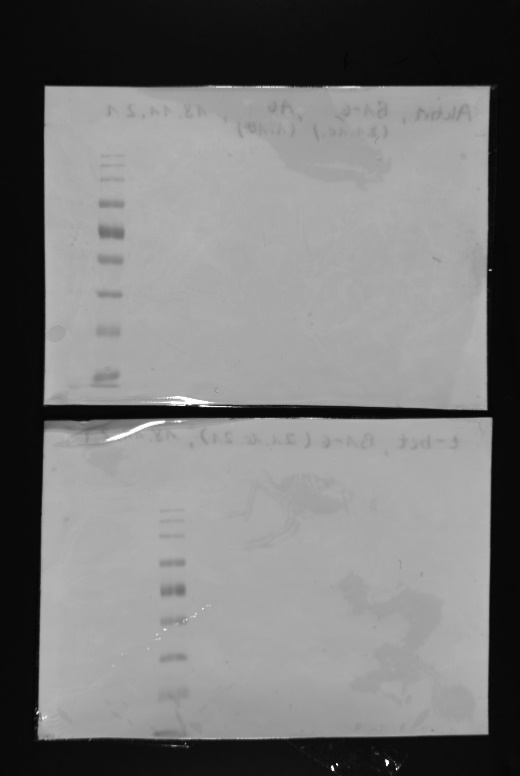 | 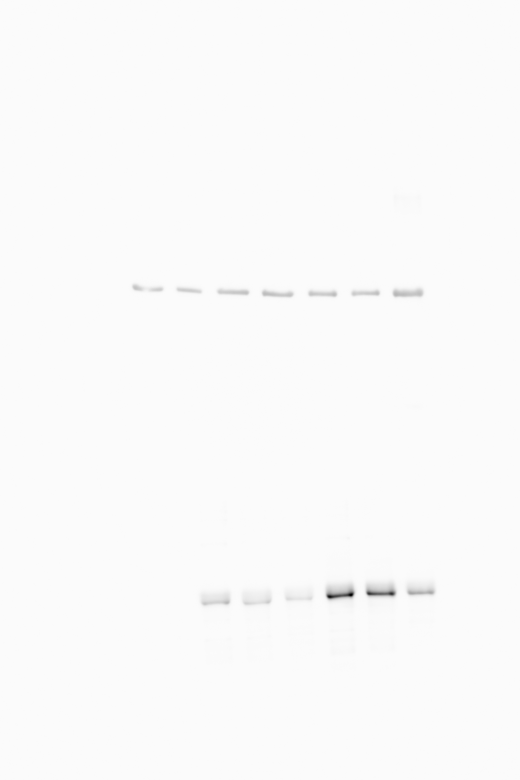 |
| A 21.12.2021 | 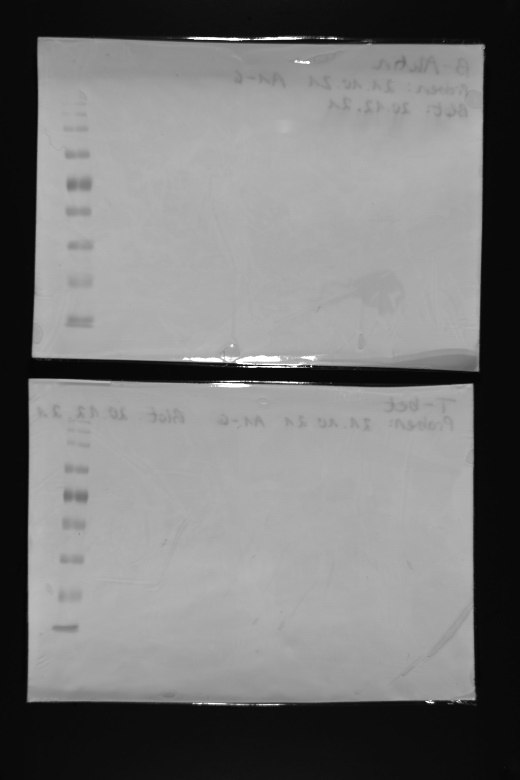 | 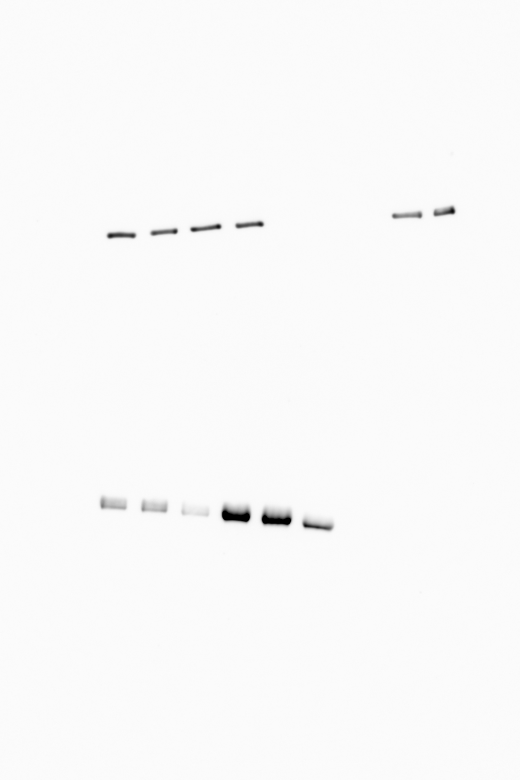 |  | 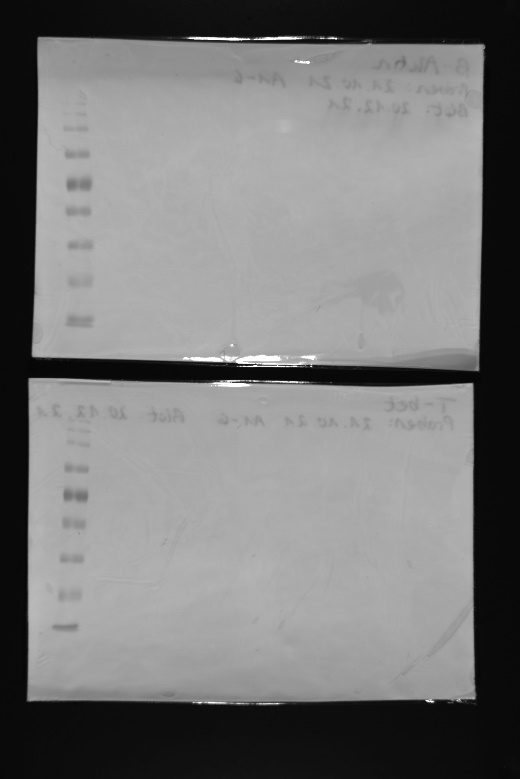 | 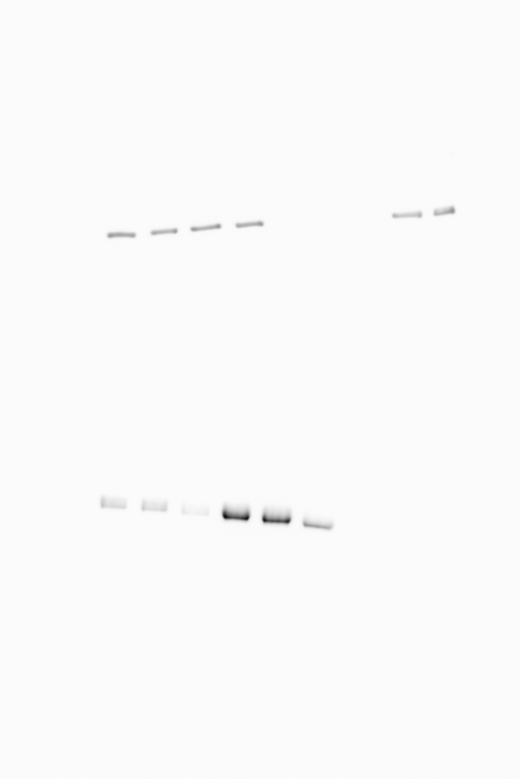 |
| J 27.01.2022 | 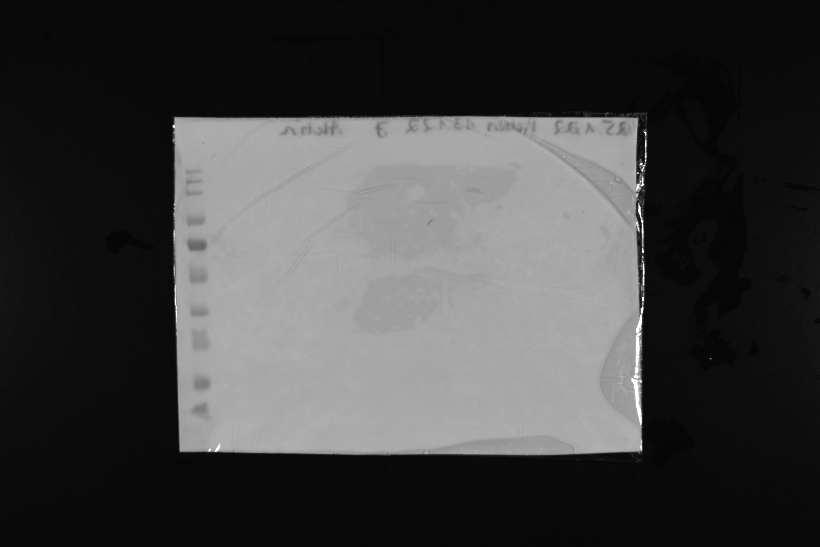 | 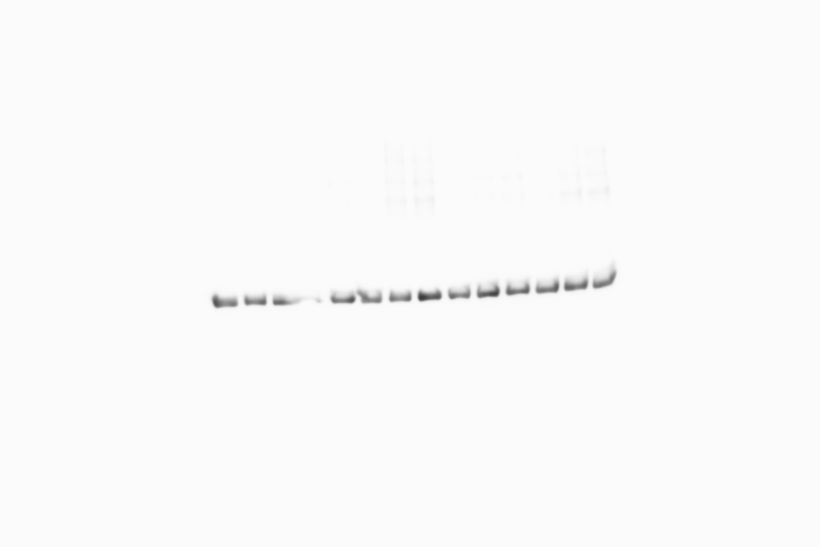 |  | 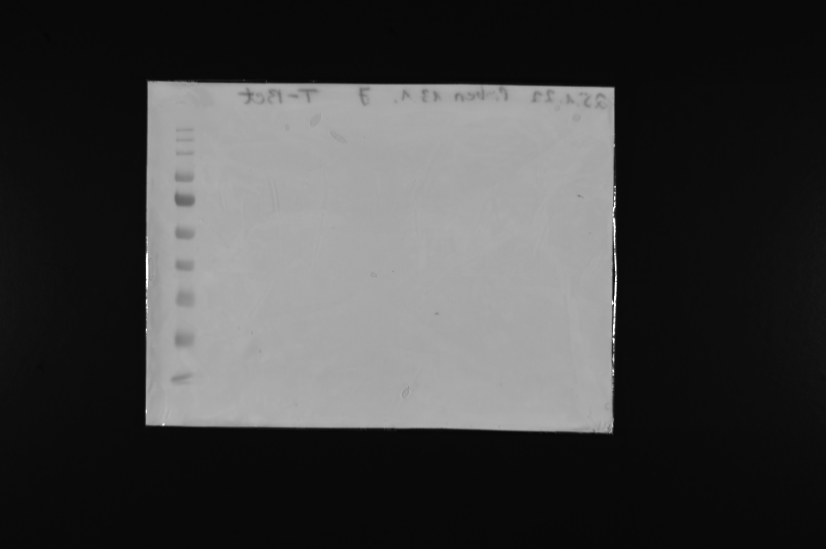 | 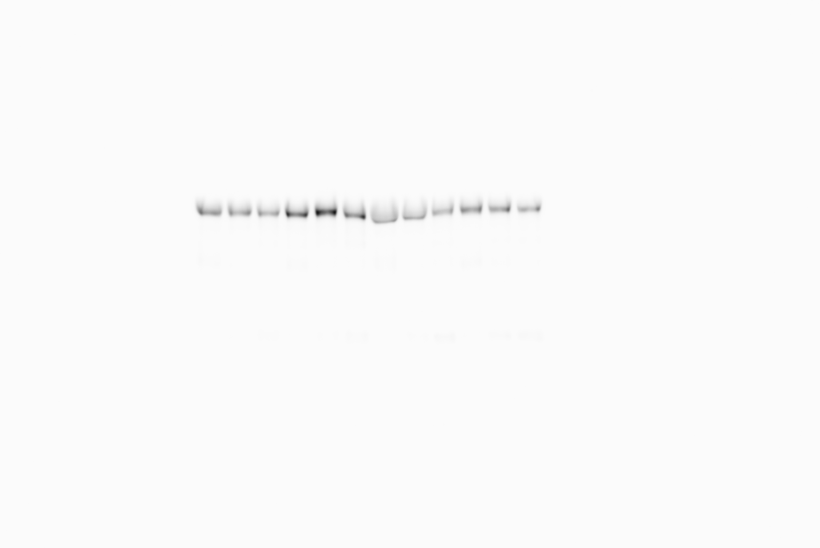 |
| S 27.01.2022 | 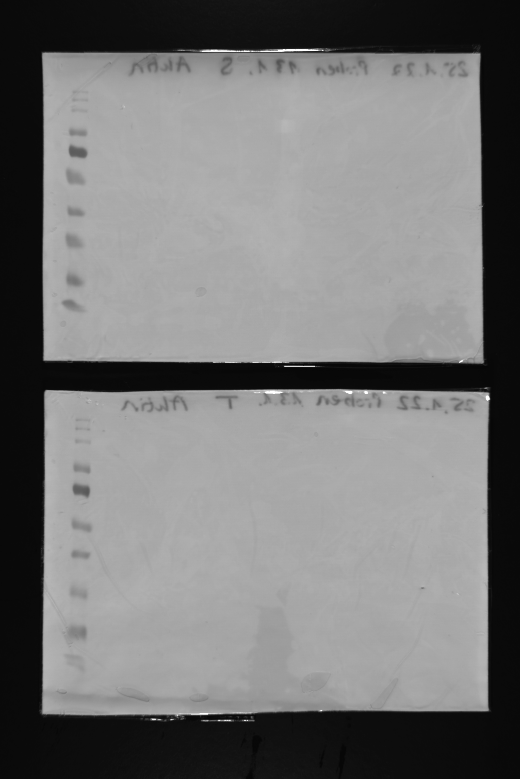 | 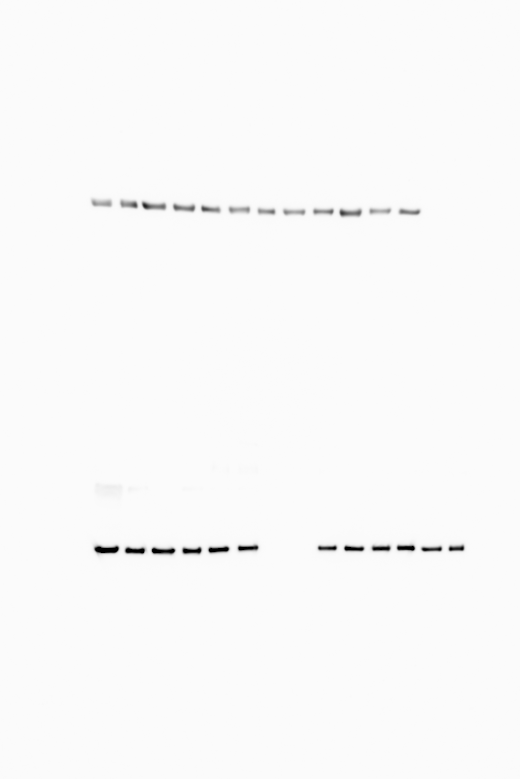 |  | 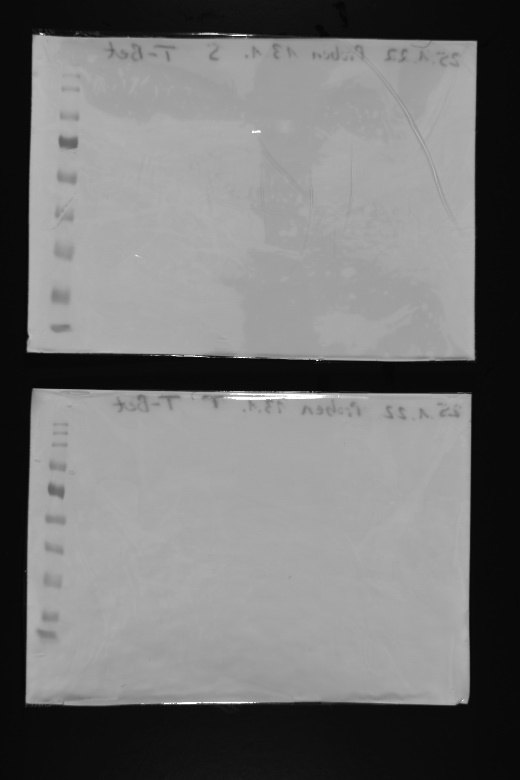 | 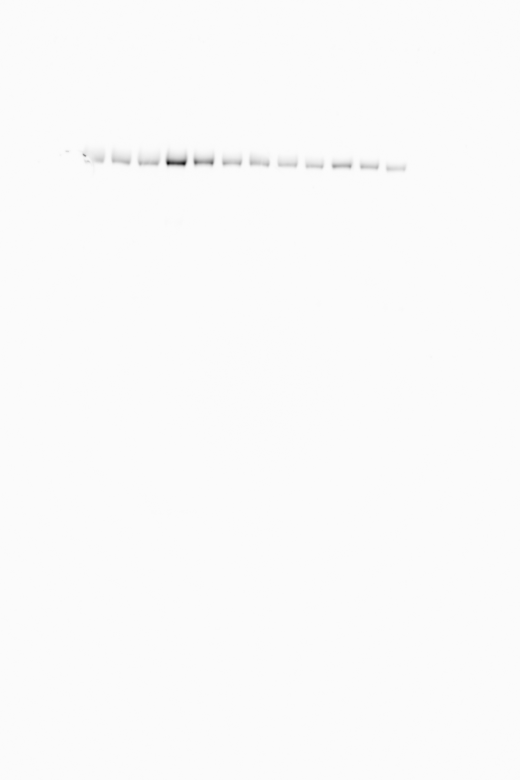 |
| T 27.01.2022 | 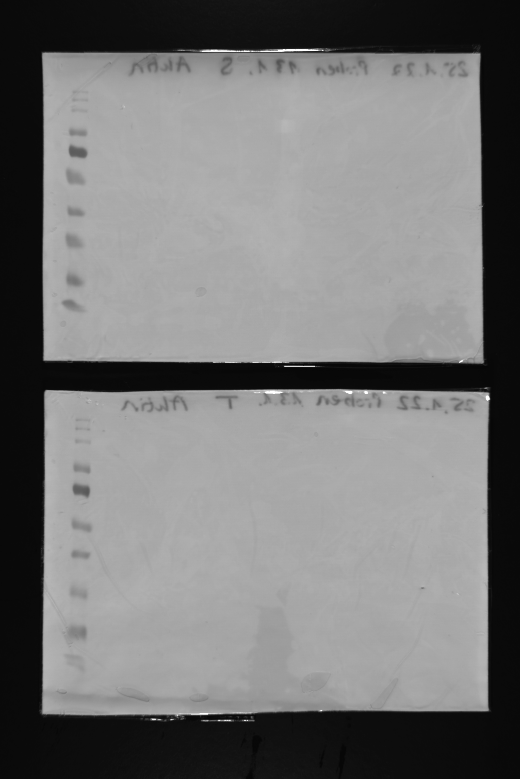 | 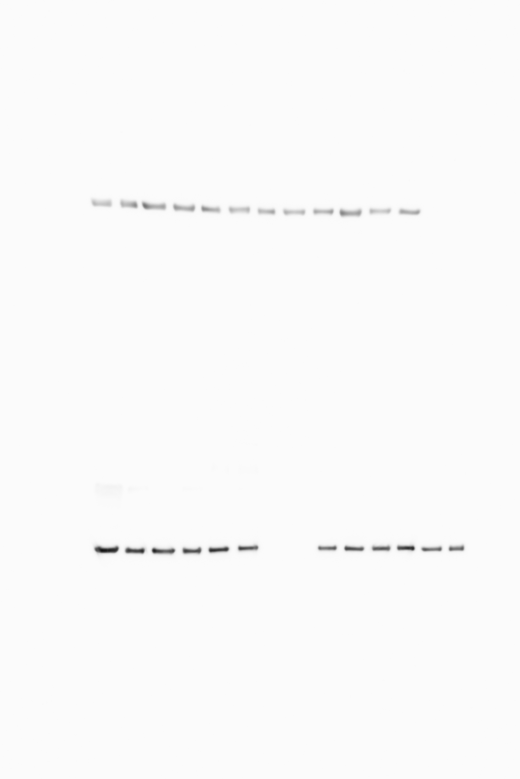 |  | 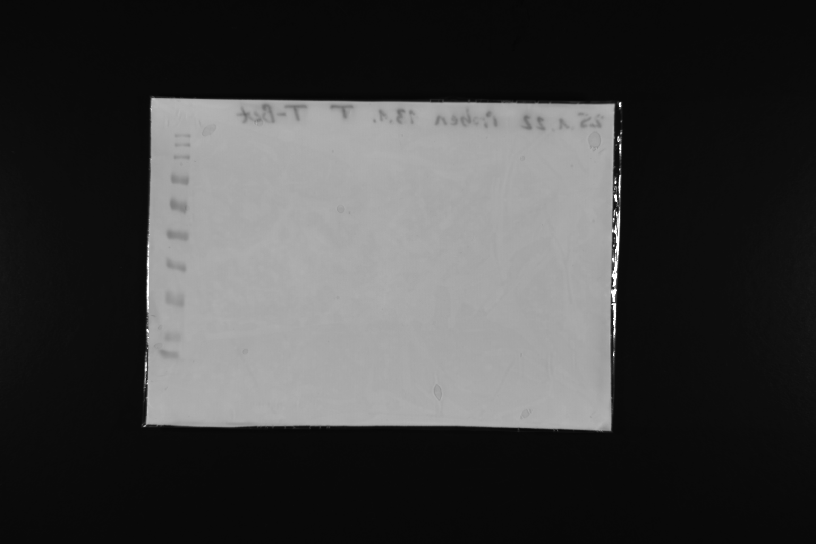 | 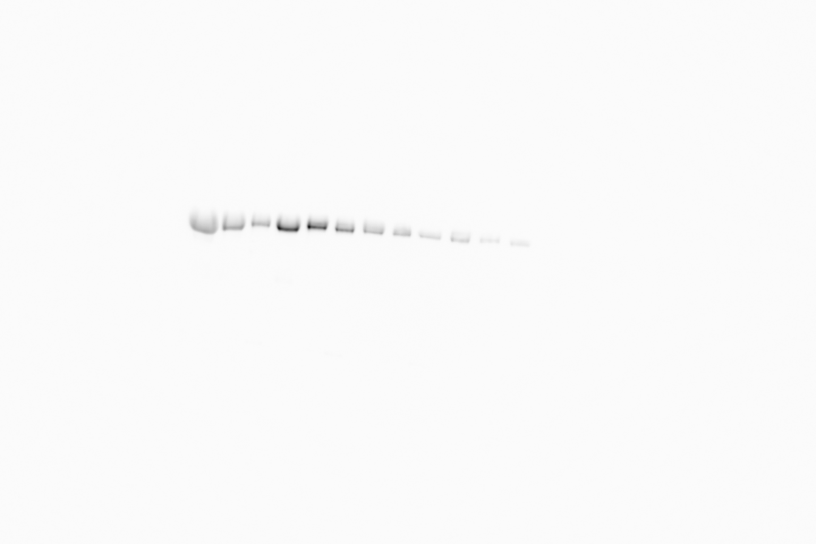 |
| E 25.02.2022 | Same blot as J 25.02.2022 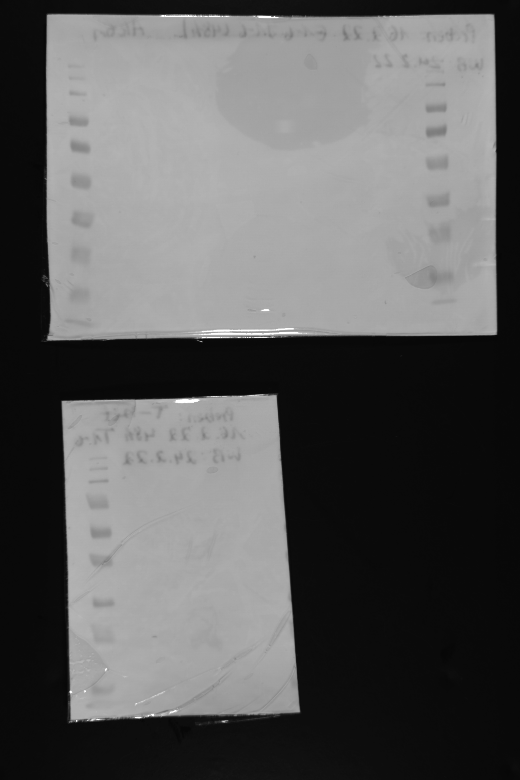 | Same blot as J 25.02.2022 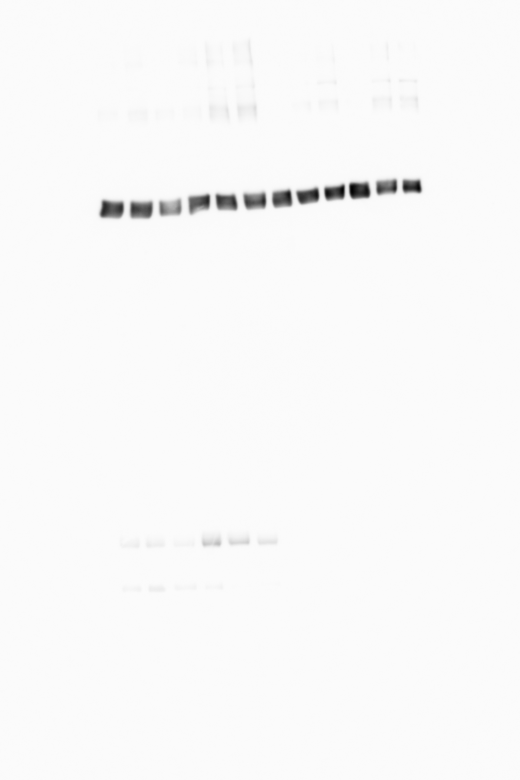 |  | Same blot as J 25.02.2022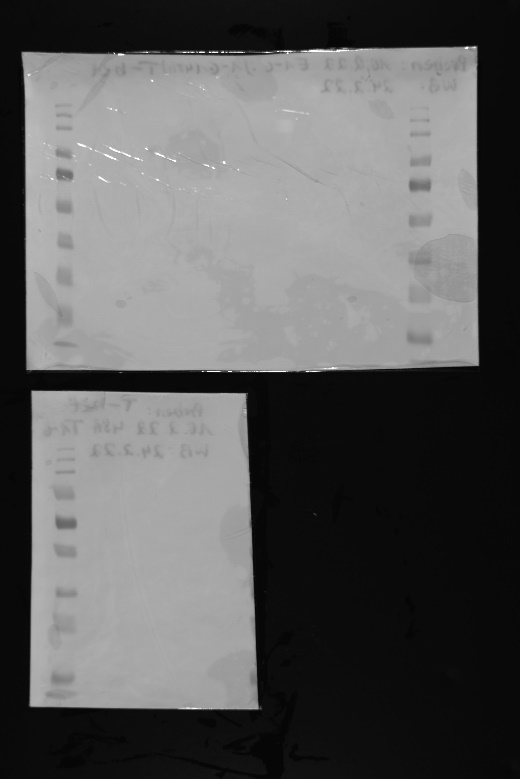 | Same blot as J 25.02.2022 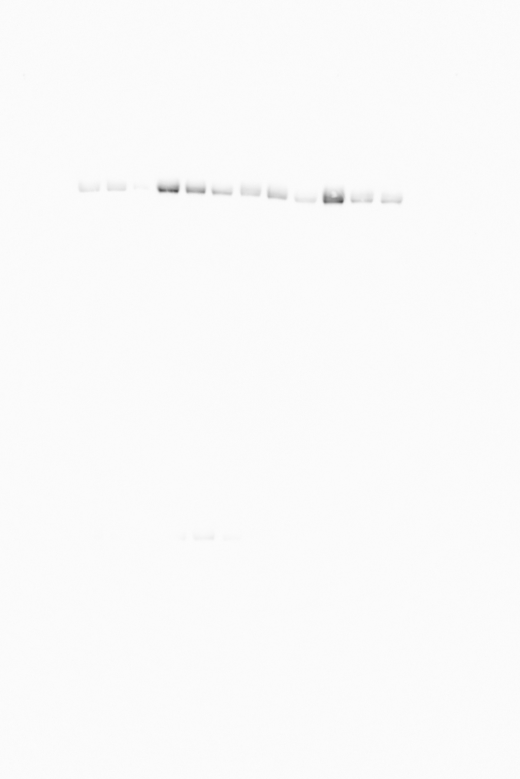 |
| J 25.02.2022 | Same blot as E 25.02.2022 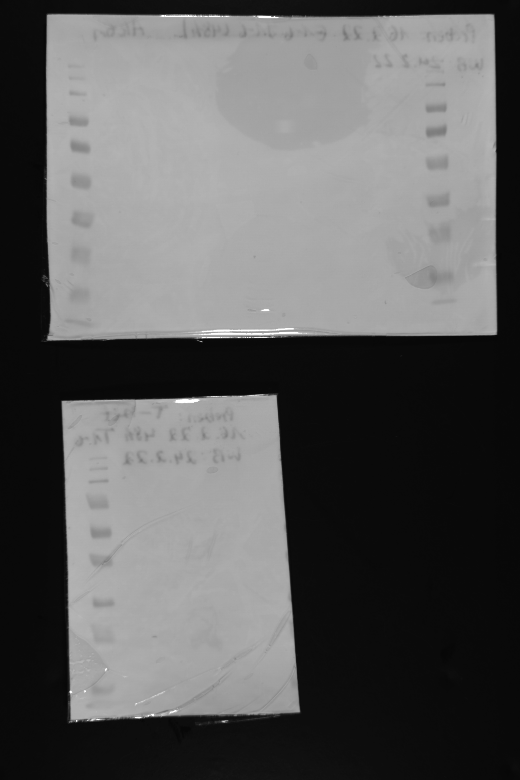 | Same blot as E 25.02.2022 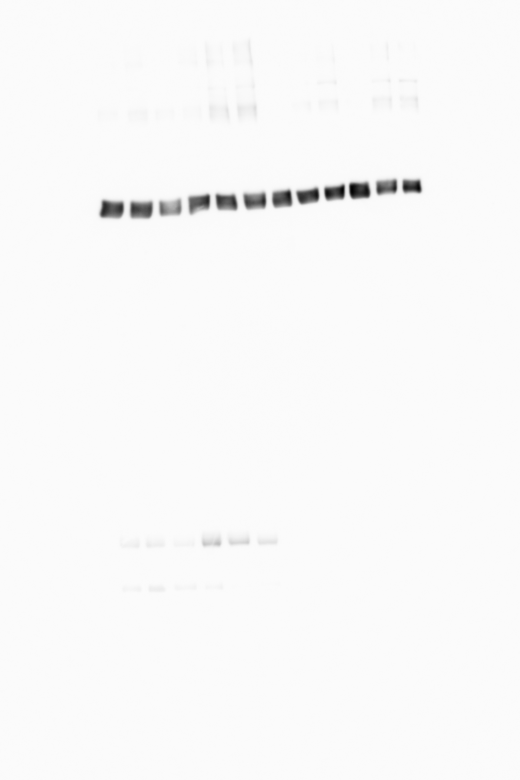 |  | Same blot as E 25.02.2022 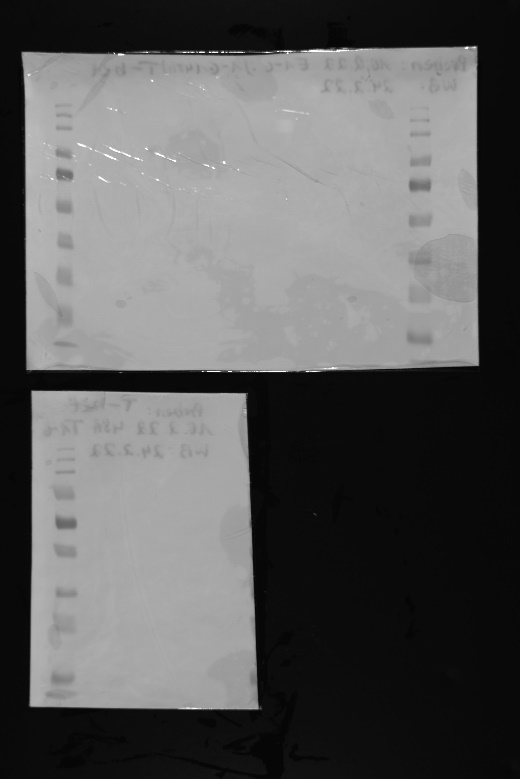 | Same blot as E 25.02.2022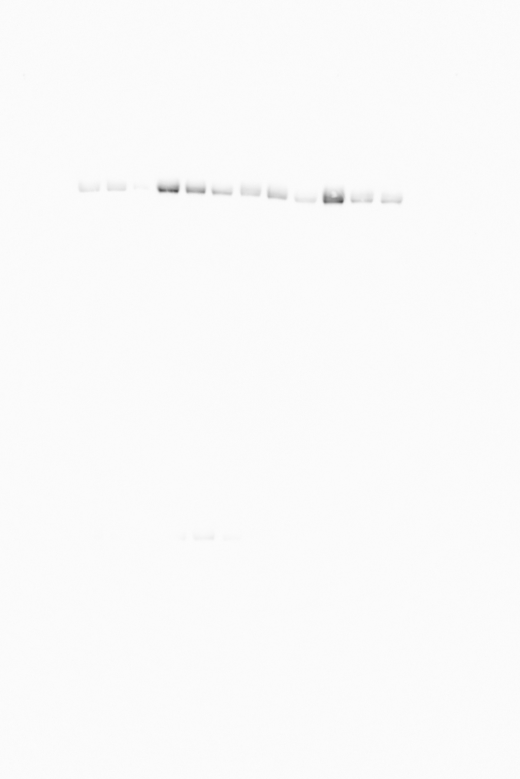 |
| T 25.02.2022 | 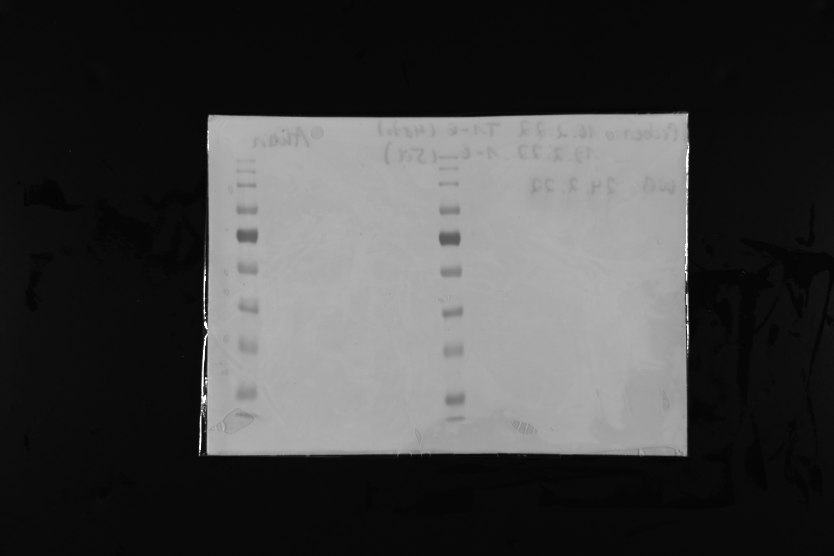 | 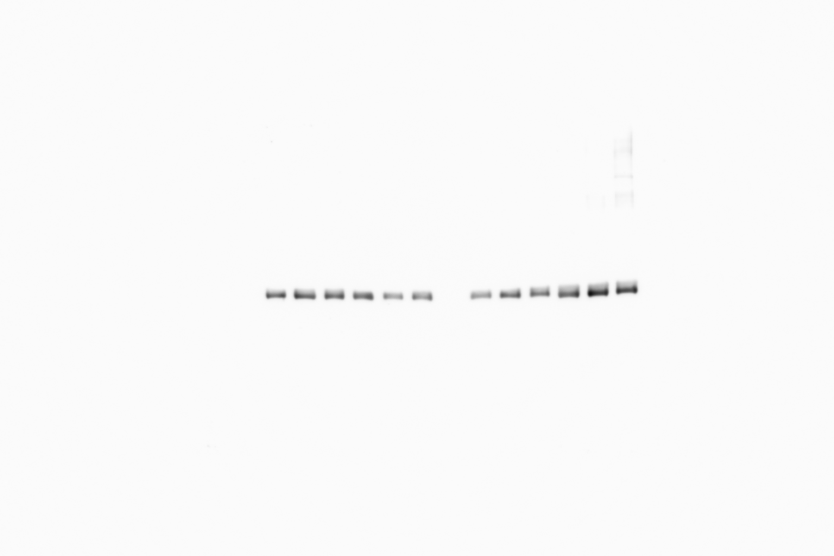 |  | 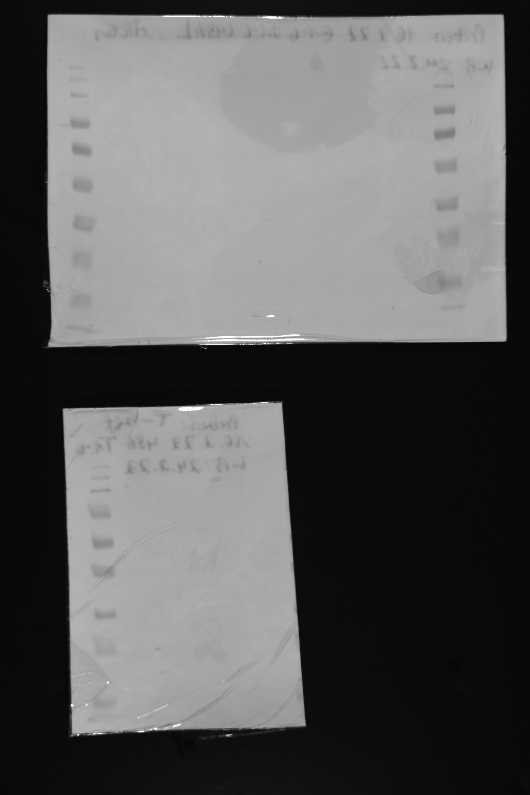 | 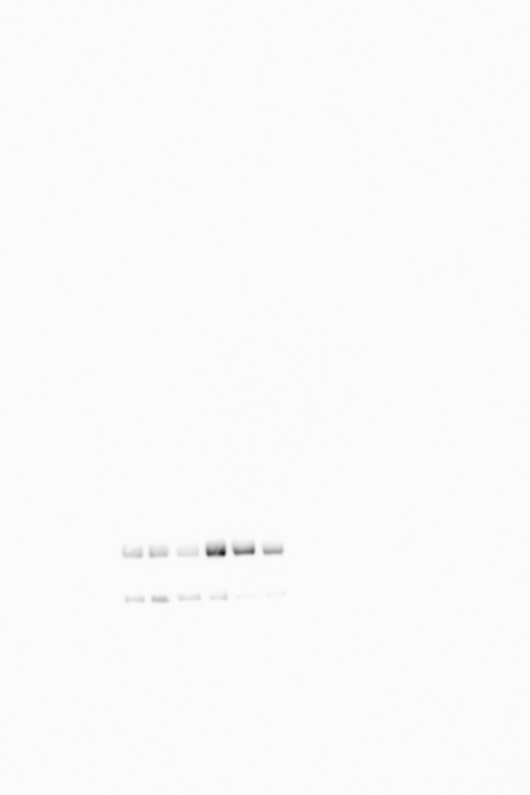 |
| A 10.01.2023 | 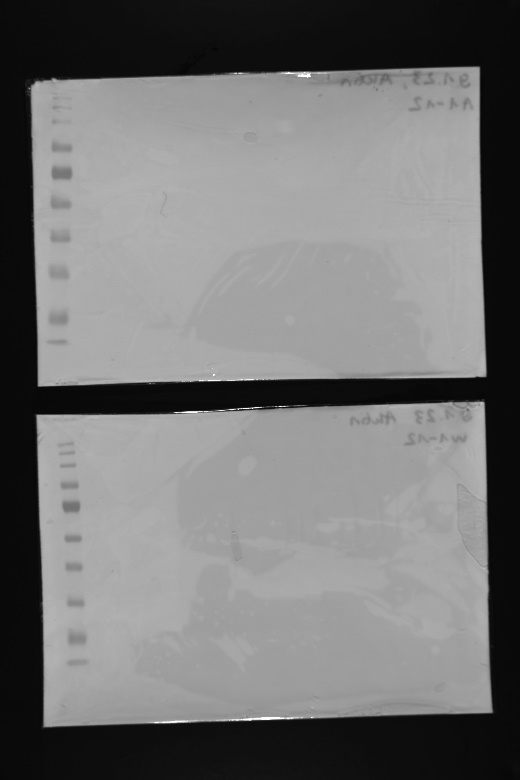 | 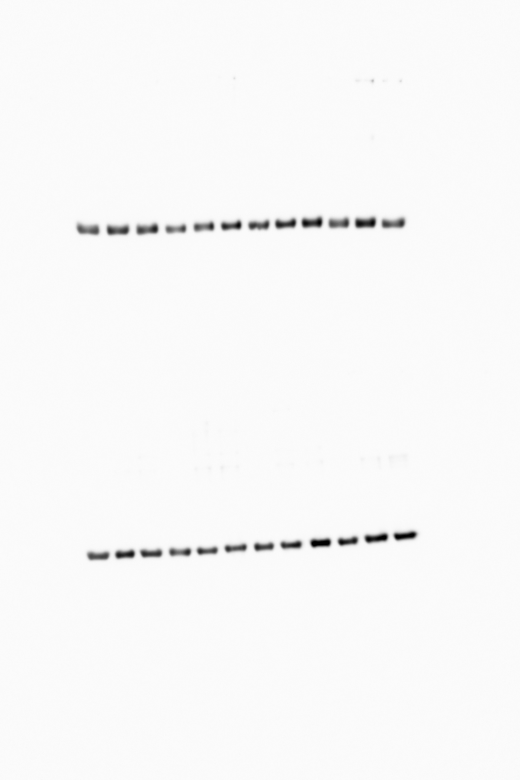 |  | 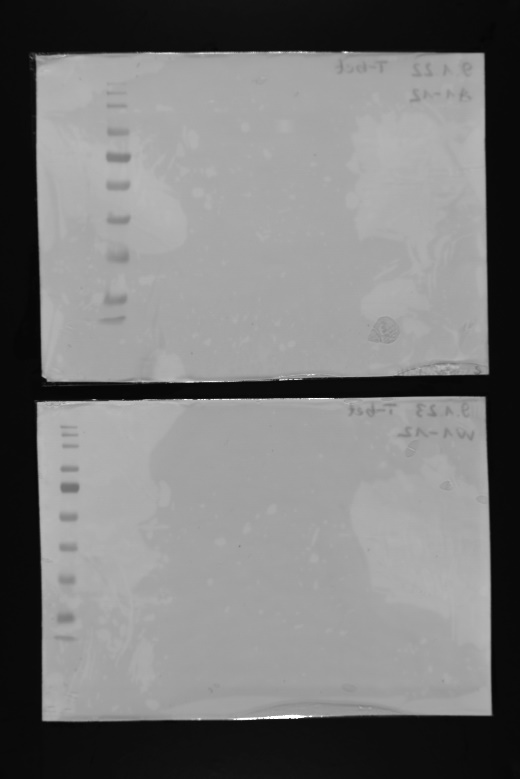 | 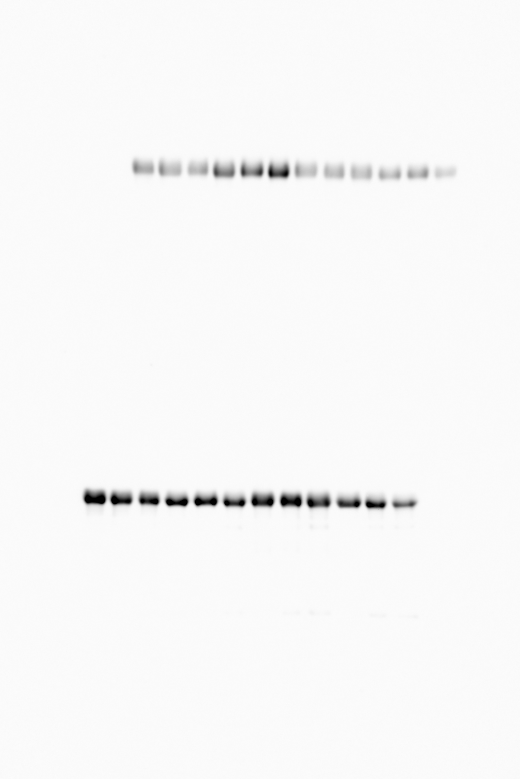 |
| H 15.02.2023 | 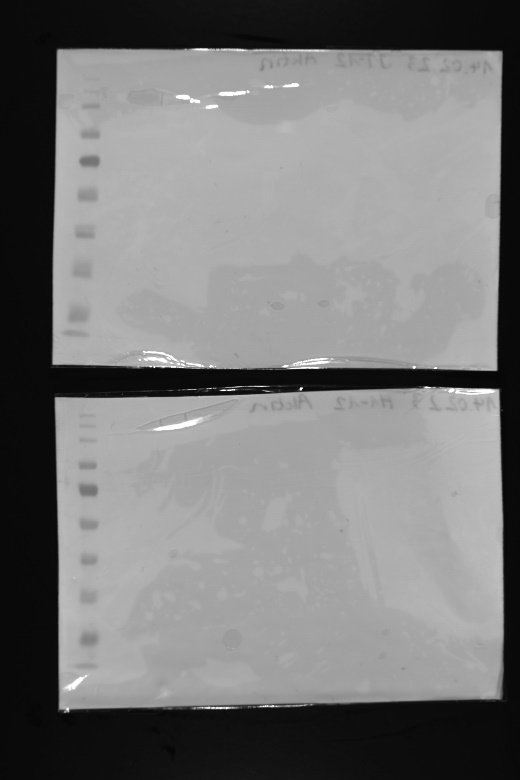 | 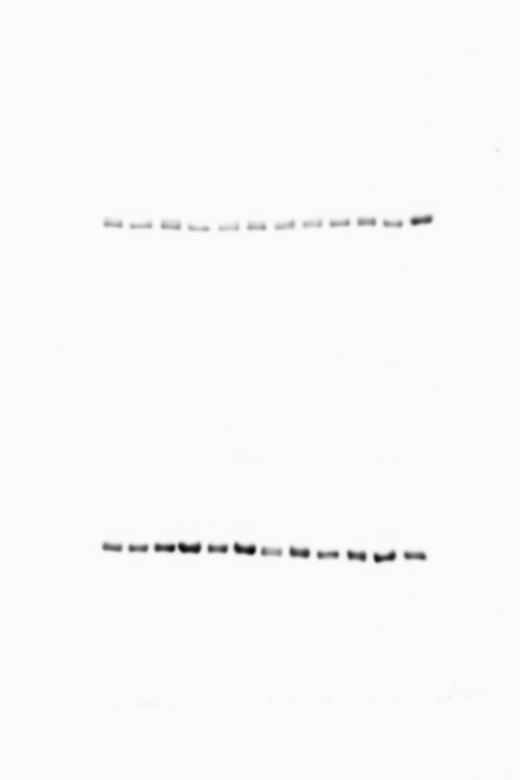 |  | 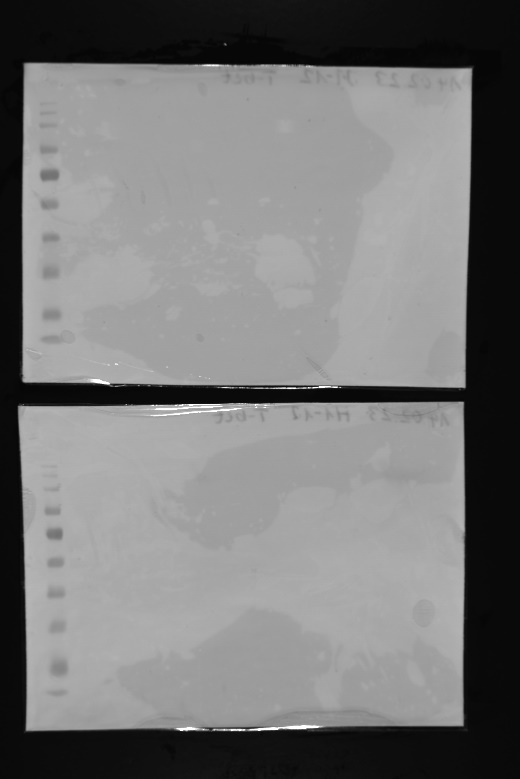 | 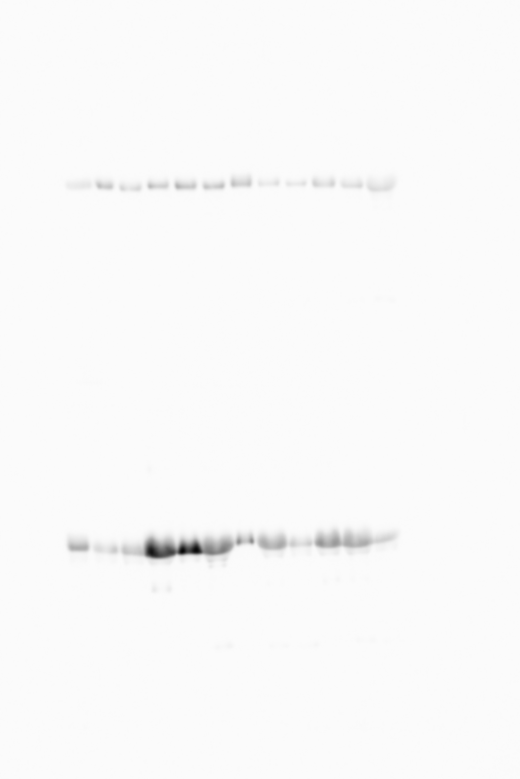 |
| J 15.02.2023 | 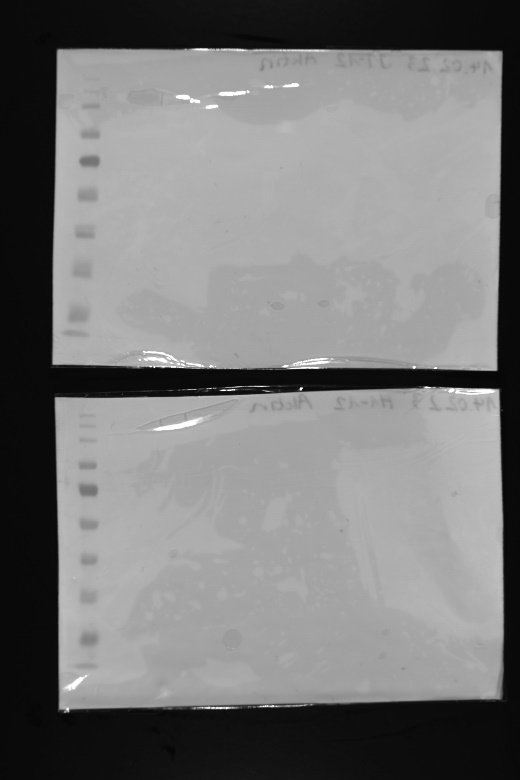 | 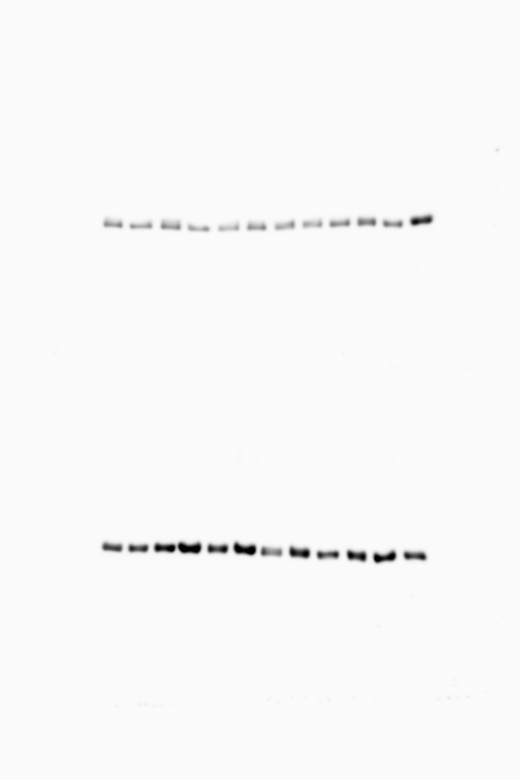 |  | 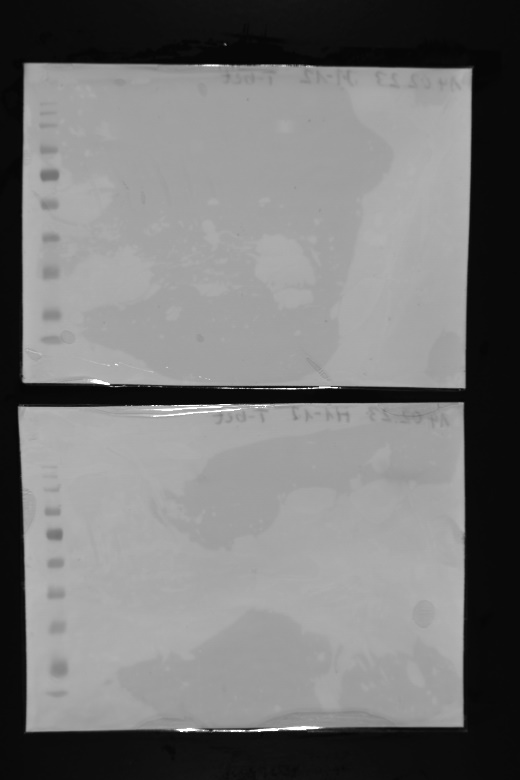 | 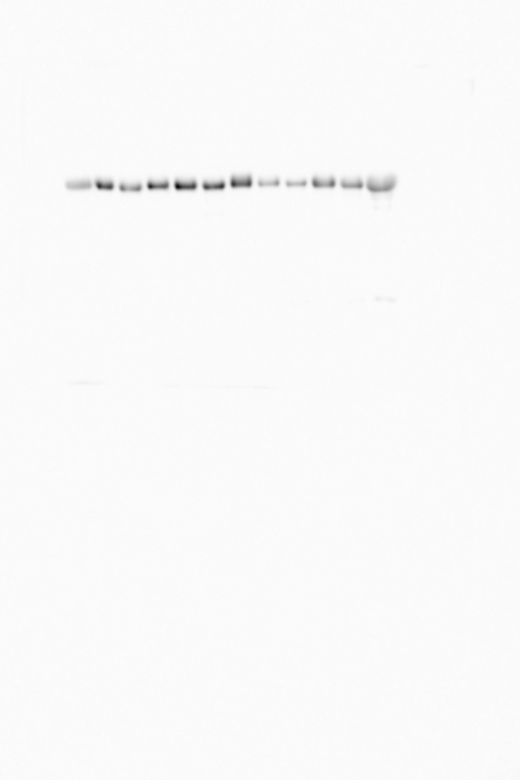 |

Fig 4 A:

Lanes as described in Fig. 4A:

| Control (same blots and lanes as is Fig. 3D) | p38 inhibitor |
| --- | --- |
| Control \| P0.35 \| P0.5 \| T50 \| T50+P0.35 \| T50 + P0.5 | Control \| P0.35 \| P0.5 \| T50 \| T50+P0.35 \| T50 + P0.5 |

|  | β−Actin | |  | T-bet | |
| --- | --- | --- | --- | --- | --- |
|  | Original molecular weight ladder | Original chemiluminescent blot |  | Original molecular weight ladder | Original chemiluminecent blot |
| J 27.01.2022 | 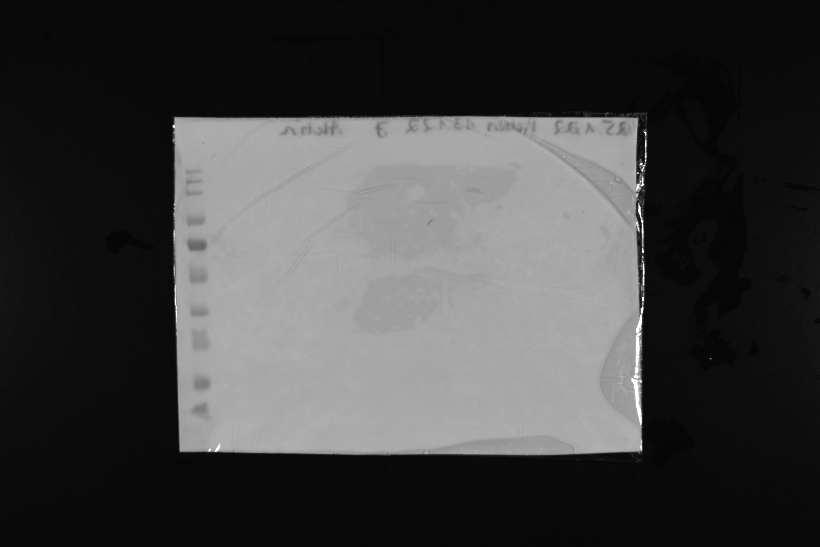 | 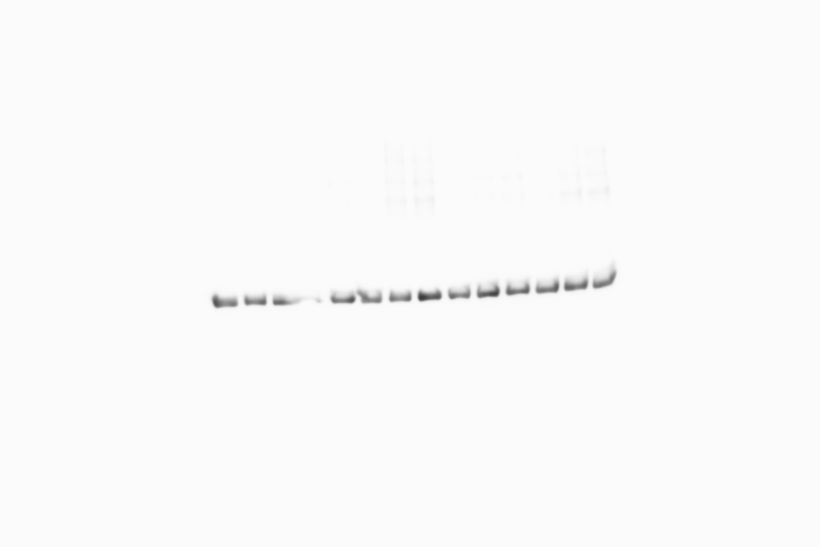 |  | 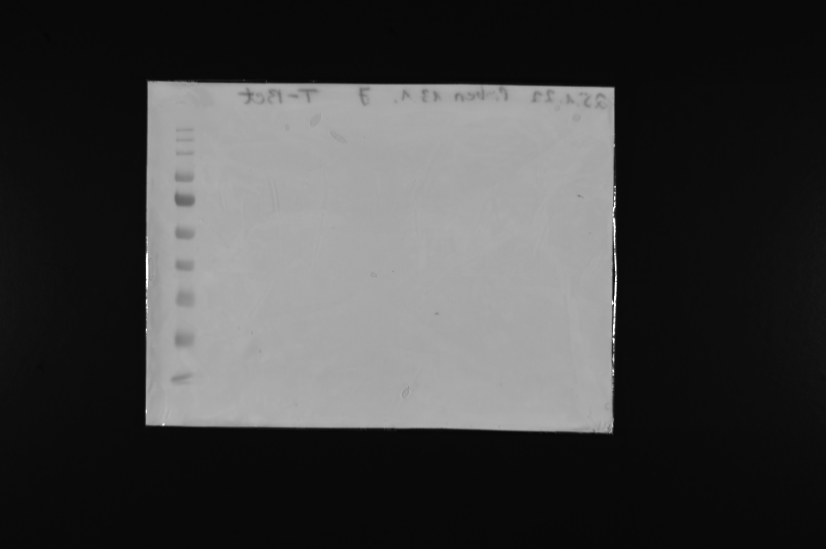 | 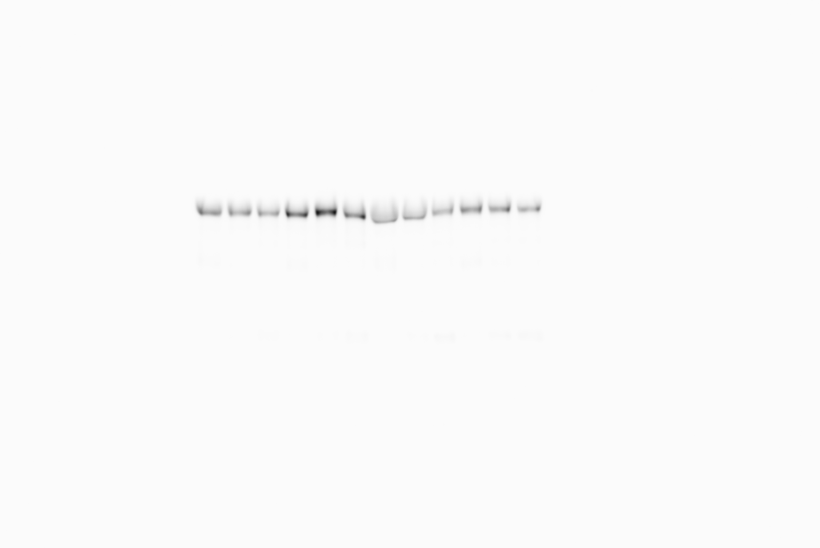 |
| S 27.01.2022 | 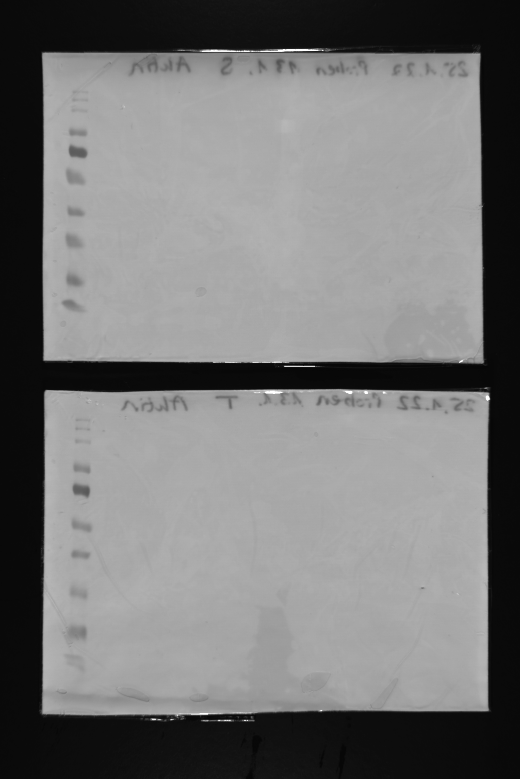 | 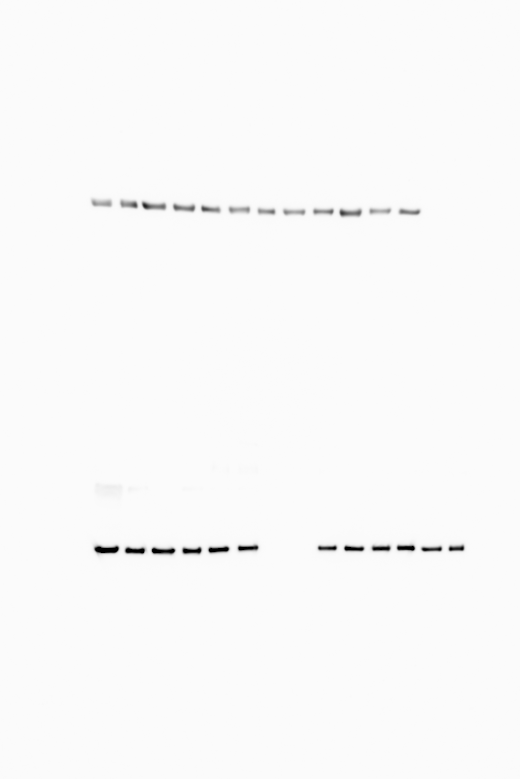 |  | 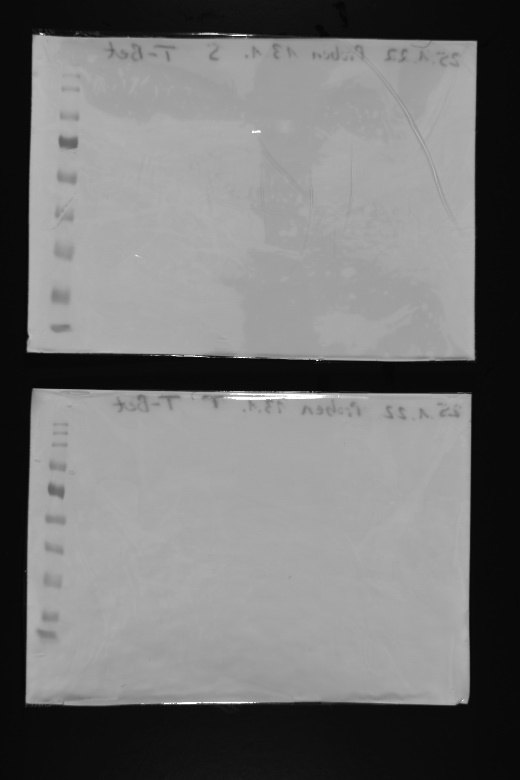 | 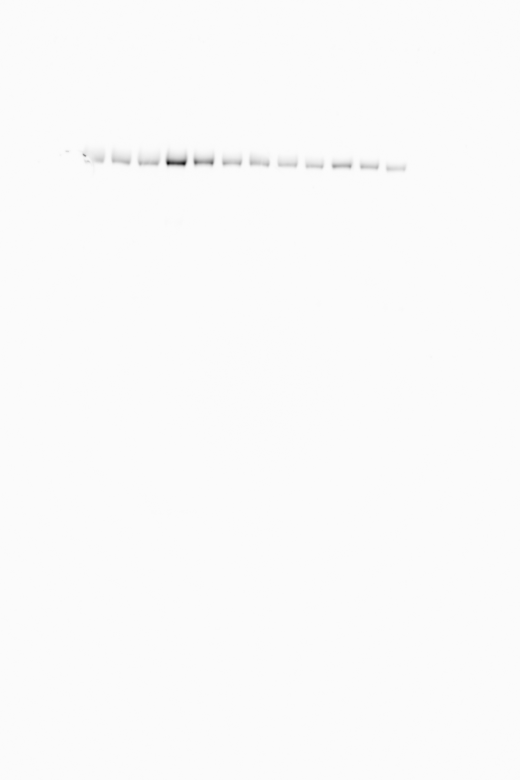 |
| T 27.01.2022 | 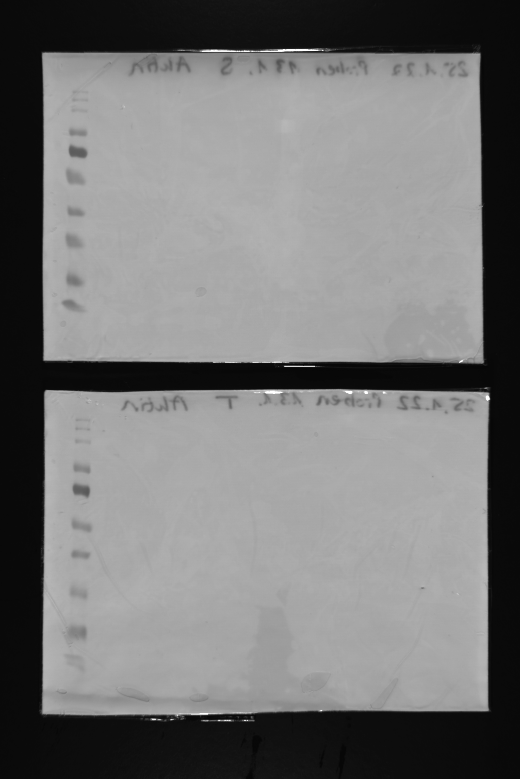 | 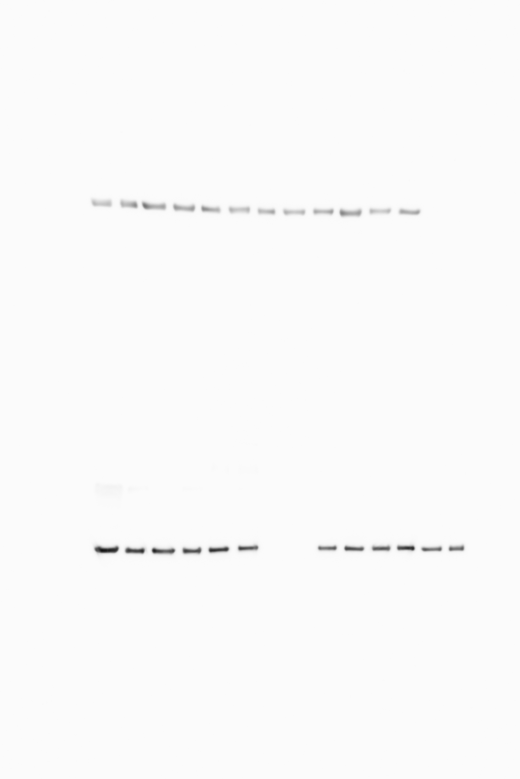 |  | 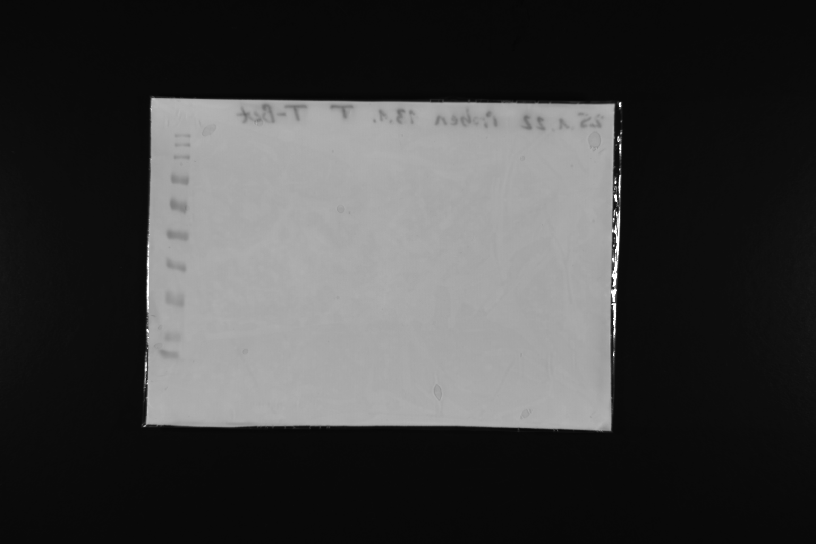 | 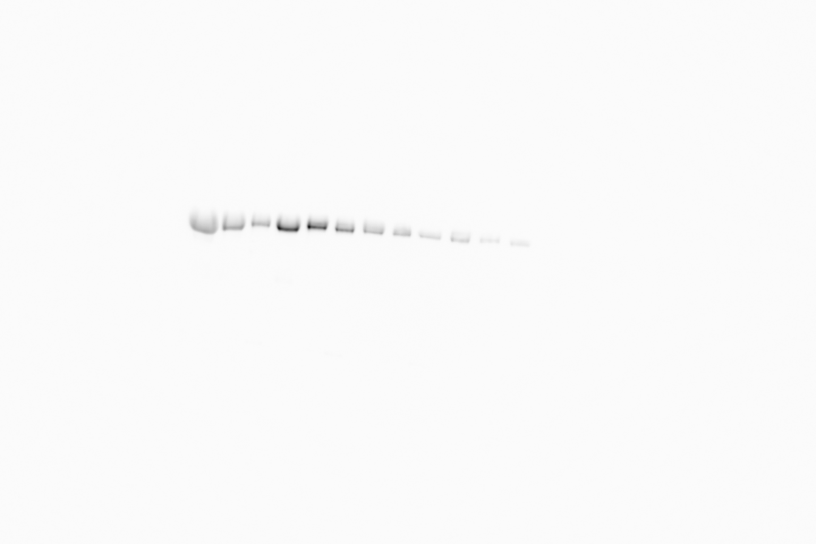 |
| A 10.01.2023 | 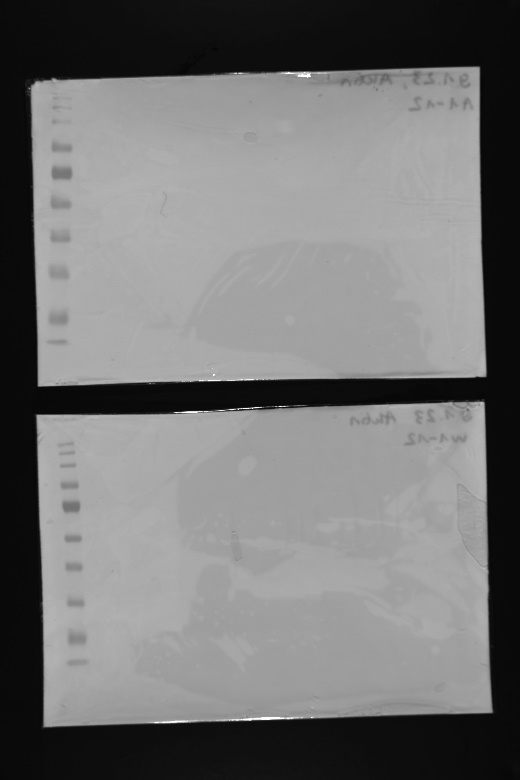 | 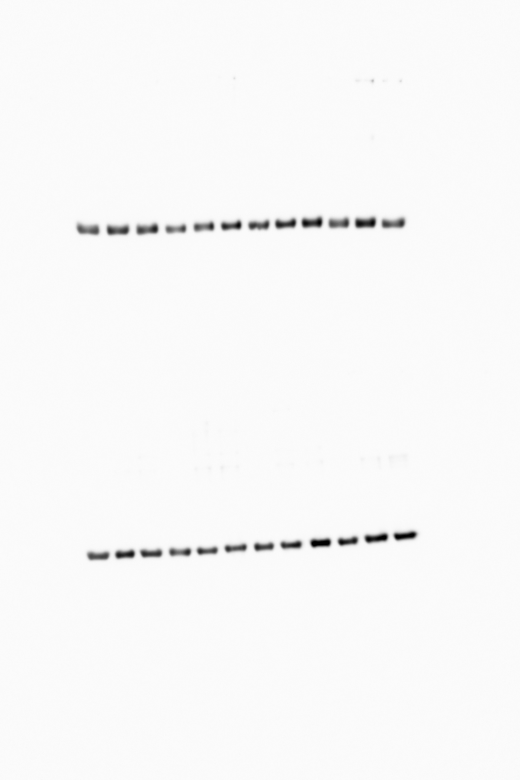 |  | 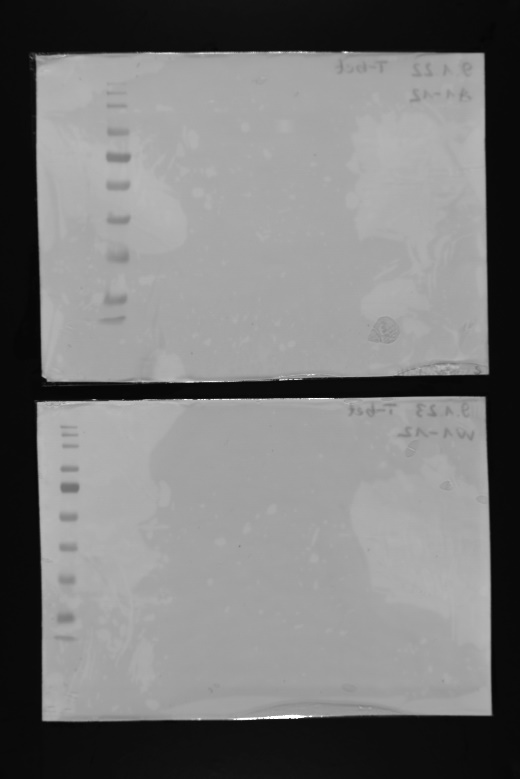 | 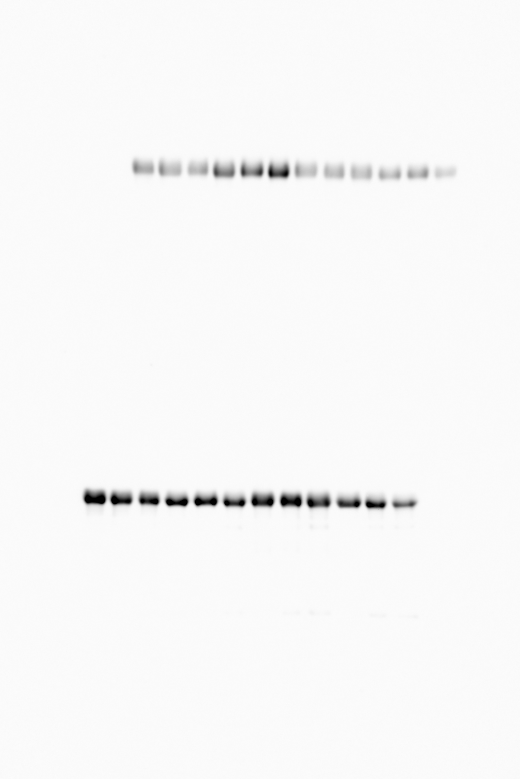 |
| H 15.02.2023 | 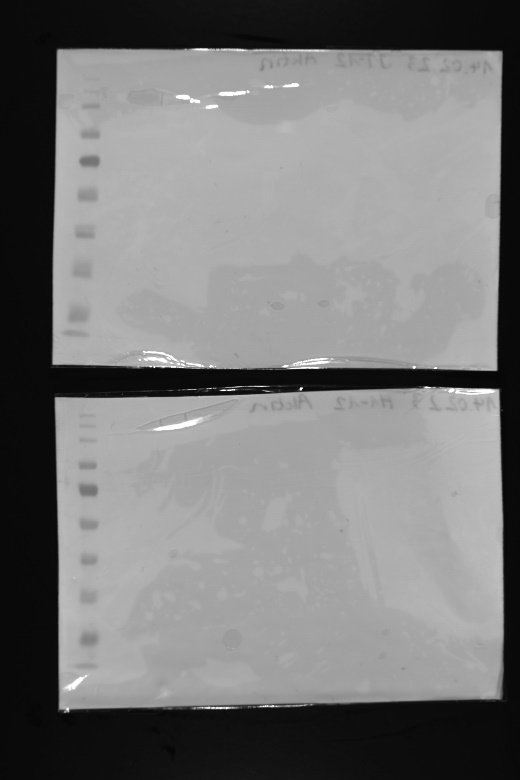 | 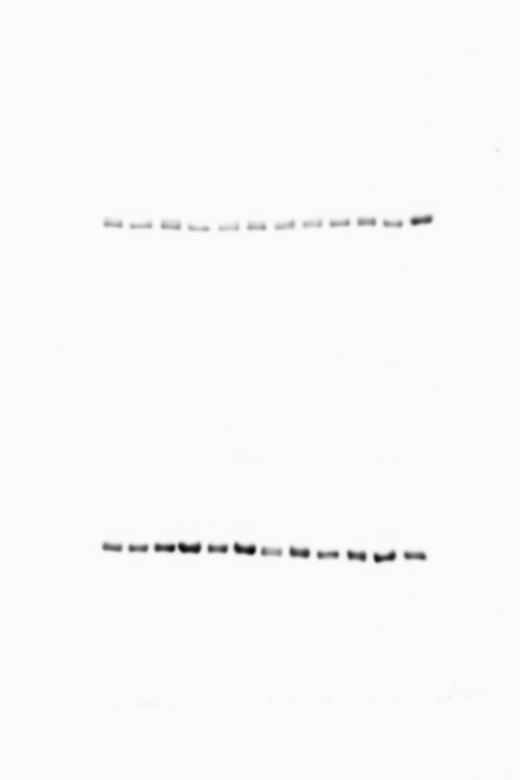 |  | 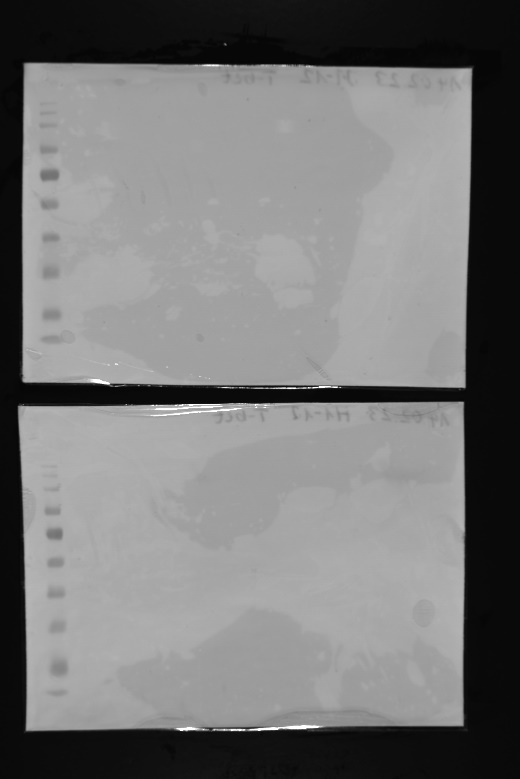 | 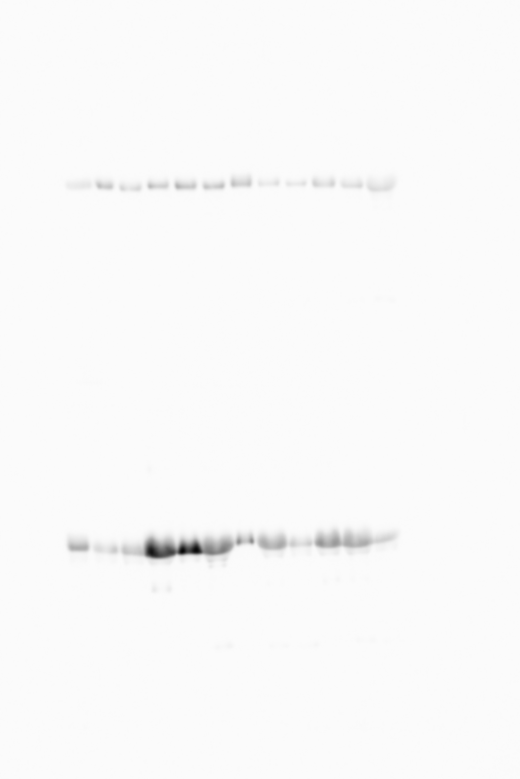 |
| J 15.02.2023 | 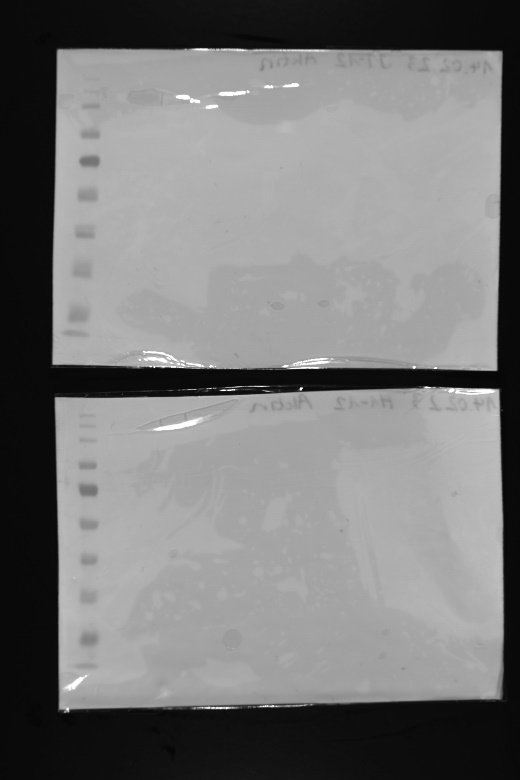 | 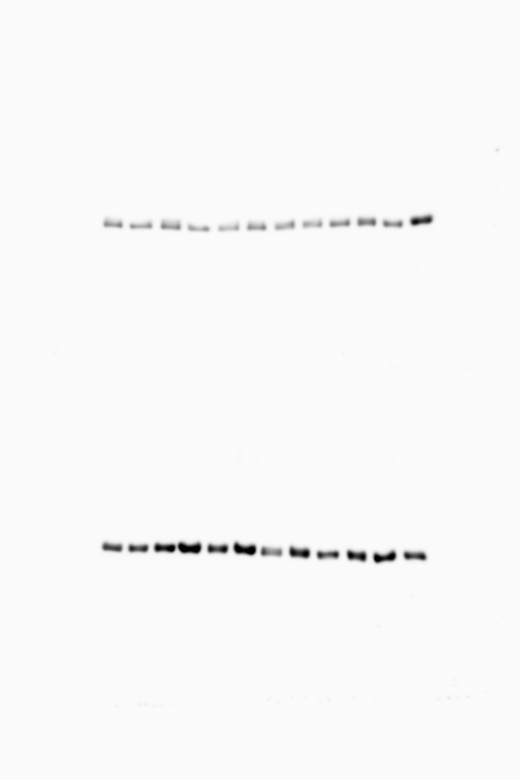 |  | 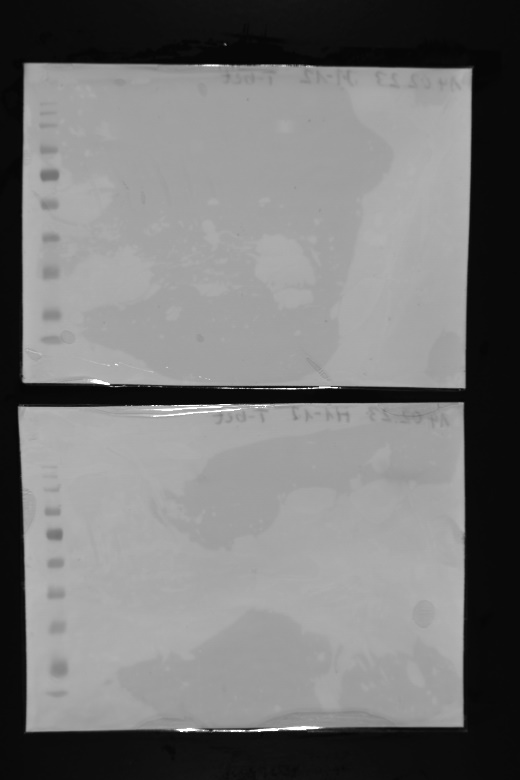 | 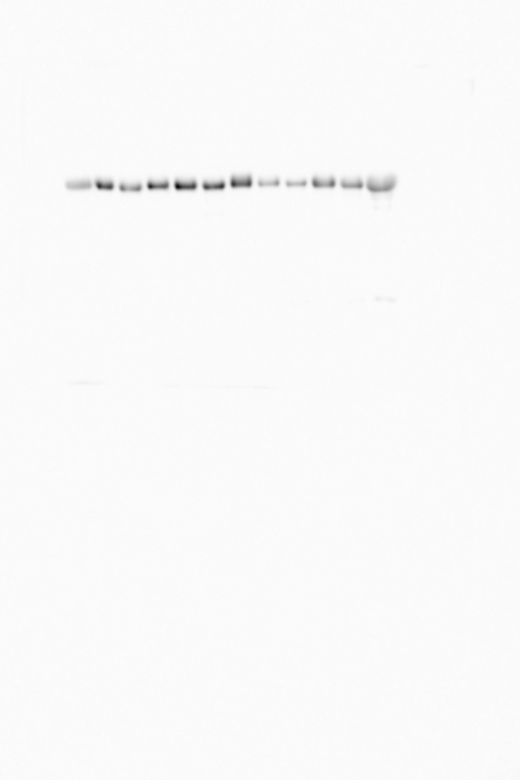 |

Fig. 6 C and D:

Lanes as described in Fig. 6C and D: Control | Pyr 0.35 | Pyr 0.5 | Thaps 50 | Thaps 50 + Pyr 0.35 | Thaps 50 + Pyr 0.5

GATA-3 and RORC2 are normalized to β−Actin. The according β−Actin is shown in Fig. 3D!

|  | GATA-3 (Fig. 6C) | |  | RORC2 (Fig. 6D) | |
| --- | --- | --- | --- | --- | --- |
|  | Original molecular weight ladder | Original chemiluminescent blot |  | Original molecular weight ladder | Original chemiluminecent blot |
| M 08.10.2021 |  |  |  |  |  |
| A 08.10.2021 |  |  |  |  |  |
| K 22.10.2021 |  |  |  |  |  |
| B 19.11.2021 |  |  |  |  |  |
| A 21.12.2021 |  |  |  |  |  |

Fig. 7 A:

Lanes as described in Fig. 7 A: Control | Pyr 0.35 | Pyr 0.5 | Thaps 50 | Thaps 50 + Pyr 0.35 | Thaps 50 + Pyr 0.5

|  | β−Actin | |  | FoxP3 | |
| --- | --- | --- | --- | --- | --- |
|  | Original molecular weight ladder | Original chemiluminescent blot |  | Original molecular weight ladder | Original chemiluminecent blot |
| M 08.10.2021 | Same blot + lanes as in Fig. 3D | Same blot + lanes as in Fig. 3D |  |  |  |
| A 08.10.2021 | Same blot + lanes as in Fig. 3D | Same blot + lanes as in Fig. 3D |  |  |  |
| K 22.10.2021 | Same blot + lanes as in Fig. 3D | Same blot + lanes as in Fig. 3D |  |  |  |
| B 19.11.2021 | Same blot + lanes as in Fig. 3D | Same blot + lanes as in Fig. 3D |  |  |  |
| A 21.12.2021 | Same blot + lanes as in Fig. 3D | Same blot + lanes as in Fig. 3D |  |  |  |
| A 10.01.2023 | Same blot + lanes as in Fig. 3D | Same blot + lanes as in Fig. 3D |  | Same blot as W 10.01.2023 but  different lanes | Same blot as W 10.01.2023 but  different lanes |
| W 10.01.2023 |  |  |  | Same blot as A 10.01.2023 but  different lanes | Same blot as A 10.01.2023 but  different lanes |
| H 15.02.2023 | Same blot + lanes as in Fig. 3D | Same blot + lanes as in Fig. 3D |  | Same blot as J 15.02.2023 |  |
| J 15.02.2023 | Same blot + lanes as in Fig. 3D | Same blot + lanes as in Fig. 3D |  | Same blot as H 15.02.2023 |  |

Fig. 7 B:

Lanes as described in Fig. 7 B: Control | Chelex |Thaps 50 | Chelex + Thaps 50 |

|  | β−Actin | |  | FoxP3 | |
| --- | --- | --- | --- | --- | --- |
|  | Original molecular weight ladder | Original chemiluminescent blot |  | Original molecular weight ladder | Original chemiluminecent blot |
| A 15.07.2022 | Same blot as J 15.07.2022 | Same blot as J 15.07.2022 |  | Same blot as J 15.07.2022 | Same blot as J 15.07.2022 |
| J 15.07.2022 | Same blot as A 15.07.2022 | Same blot as A 15.07.2022 |  | Same blot as A 15.07.2022 | Same blot as A 15.07.2022 |
| H 01.03.2023 | Same blot as J 01.03.2023 | Same blot as J 01.03.2023 |  | Same blot as J 01.03.2023 | Same blot as J 01.03.2023 |
| J 01.03.2023 | Same blot as H 01.03.2023 | Same blot as H 01.03.2023 |  | Same blot as H 01.03.2023 | Same blot as H 01.03.2023 |
| L 30.03.2023 | Same blot as F 30.03.2023 | Same blot as F 30.03.2023 |  | Same blot as F 30.03.2023 | Same blot as F 30.03.2023 |
| F 30.03.2023 | Same blot as H 30.03.2023 | Same blot as H 30.03.2023 |  | Same blot as H 30.03.2023 | Same blot as H 30.03.2023 |
